# Synthetic Inositol Phosphate Analogs Reveal that PPIP5K2 Has a Surface-Mounted Substrate Capture Site that Is a Target for Drug Discovery

**DOI:** 10.1016/j.chembiol.2014.03.009

**Published:** 2014-05-22

**Authors:** Huanchen Wang, Himali Y. Godage, Andrew M. Riley, Jeremy D. Weaver, Stephen B. Shears, Barry V.L. Potter

**Affiliations:** 1 Inositol Signaling Group, Laboratory of Signal Transduction, National Institute of Environmental Health Sciences, National Institutes of Health, Research Triangle Park, NC 27709, USA; 2 Wolfson Laboratory of Medicinal Chemistry, Department of Pharmacy and Pharmacology, University of Bath, Bath, Somerset BA2 7AY, UK

## Abstract

Diphosphoinositol pentakisphosphate kinase 2 (PPIP5K2) is one of the mammalian PPIP5K isoforms responsible for synthesis of diphosphoinositol polyphosphates (inositol pyrophosphates; PP-InsPs), regulatory molecules that function at the interface of cell signaling and organismic homeostasis. The development of drugs that inhibit PPIP5K2 could have both experimental and therapeutic applications. Here, we describe a synthetic strategy for producing naturally occurring 5-PP-InsP_4_, as well as several inositol polyphosphate analogs, and we study their interactions with PPIP5K2 using biochemical and structural approaches. These experiments uncover an additional ligand-binding site on the surface of PPIP5K2, adjacent to the catalytic pocket. This site facilitates substrate capture from the bulk phase, prior to transfer into the catalytic pocket. In addition to demonstrating a “catch-and-pass” reaction mechanism in a small molecule kinase, we demonstrate that binding of our analogs to the substrate capture site inhibits PPIP5K2. This work suggests that the substrate-binding site offers new opportunities for targeted drug design.

## Highlights

• Chemical synthesis of 5-PP-InsP_4_ and a diphosphorylated analog
• Chemical synthesis of inositol polyphosphate analogs with hydrophobic groups
• An inositol pyrophosphate kinase has a surface-mounted, substrate capture site
• Structural and biochemical characterization of a catch-and-pass catalytic cycle

## Graphical Abstract

**Figure:**
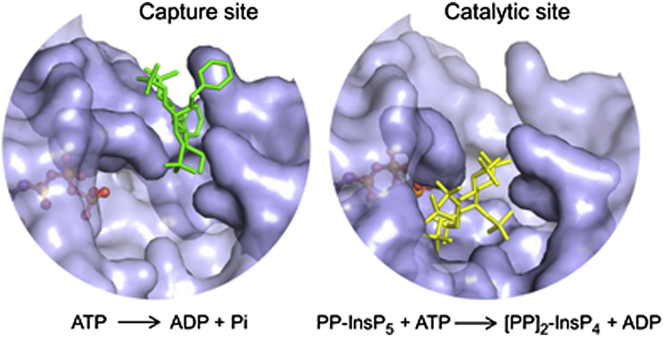

## Teaser

Wang et al show that a substrate-capture site optimizes synthesis of inositol pyrophosphates by PPIP5K. The site attracts substrate from the bulk phase then delivers it into the proximal catalytic pocket. The capture site was uncovered through its affinity for new inositol pyrophosphate analogs.

## Introduction

**Figure 1:**
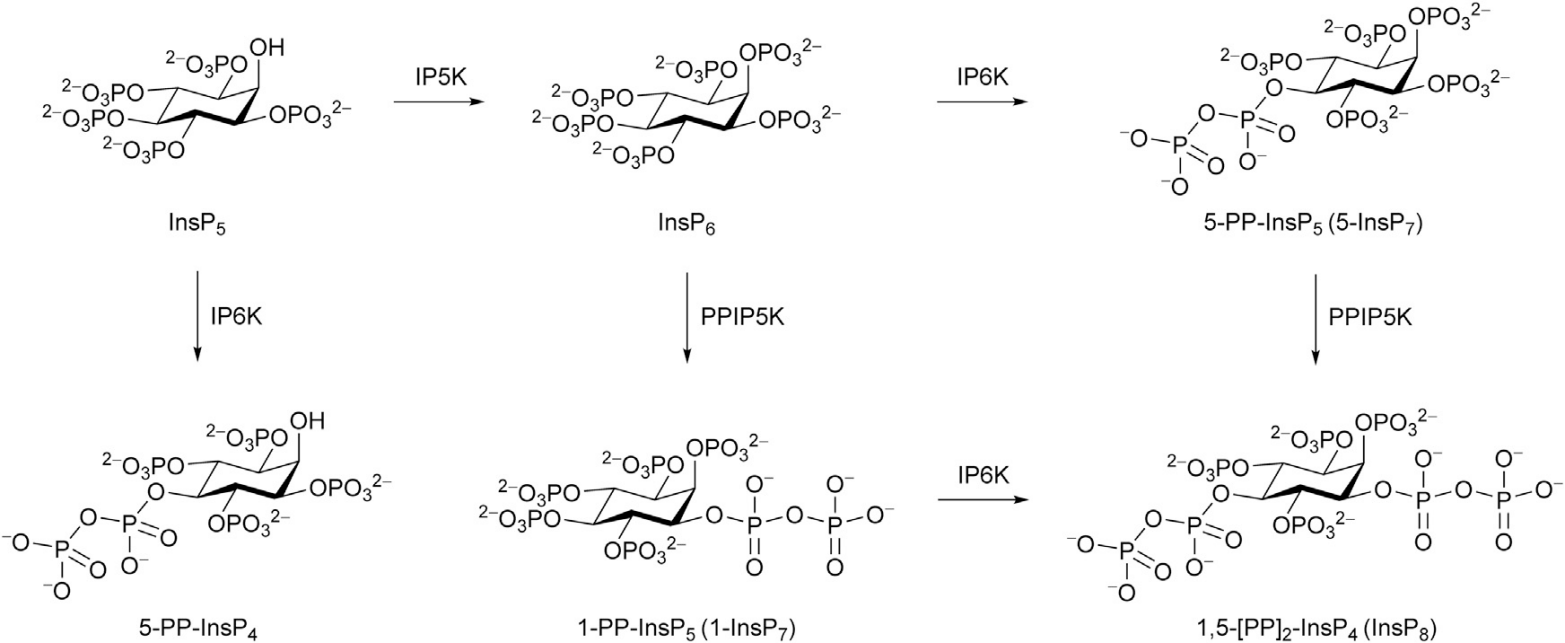
Biosynthesis of Diphosphoinositol Phosphates IP5K, inositol pentakisphosphate 2-kinase; IP6K, inositol hexakisphosphate 5-kinase; PPIP5K, diphosphoinositol pentakisphosphate 1-kinase.

The process of signal transduction that governs many cellular activities frequently relies upon evolutionarily conserved families of small, regulatory molecules. Among them are the diphosphoinositol polyphosphates (inositol pyrophosphates: 5-PP-InsP_4_, 1-PP-InsP_5_ [1-InsP_7_], 5-PP-InsP_5_ [5-InsP_7_], and 1,5-[PP]_2_-InsP_4_ [InsP_8_]; Figure 1), in which six to eight phosphate groups are crammed around the six-carbon inositol ring. These high-energy molecules are synthesized by two distinct classes of kinases, IP6Ks and PPIP5Ks. The IP6Ks add the 5-diphosphate group (Draskovic et al., 2008); mammals express three IP6K isoforms (Thomas and Potter, 2014). The PPIP5Ks synthesize the 1-diphosphate (Wang et al., 2012); there are two isoforms in mammals (Thomas and Potter, 2014). Interest in this field has recently been heightened by demonstrations that diphosphoinositol polyphosphates operate at the interface of cell signaling and organismic homeostasis (Choi et al., 2005; Szijgyarto et al., 2011; Shears, 2009; Illies et al., 2007; Chakraborty et al., 2010; Pulloor et al., 2014). Here, a dynamic balance between the activities of IP6Ks and PPIP5Ks is of particular significance. For example, the synthesis of 5-PP-InsP_5_ by IP6Ks inhibits the PtdIns(3,4,5)P_3_/PDK1/AKT/mechanistic target of rapamycin (mTOR) cascade (Chakraborty et al., 2010) that controls cell growth and metabolism in response to changes in levels of nutrients, growth factors, and bioenergetic status (Benjamin et al., 2011). This inhibitory action of 5-PP-InsP_5_ is reversed through its further phosphorylation by the PPIP5Ks (Gokhale et al., 2013). There may be therapeutic value in inhibiting PPIP5K activity to elevate 5-PP-InsP_5_ levels and attenuate the mTOR pathway, which is hyperactivated in 70% of human tumors, contributing to the derangement of cell growth and metabolism that accompanies cancer development and progression (Benjamin et al., 2011). We recently published proof-of-principle of the latter idea by demonstrating that AKT phosphorylation in myoblasts is inhibited when PPIP5K1 expression is “knocked-down” (Gokhale et al., 2013). It is just such therapeutic motives that frequently drive the development of drugs that can specifically target kinases such as PPIP5Ks. Candidate molecules may be rationally designed when information on protein structure is available. To this end, we recently solved the structure of the N-terminal kinase domain of PPIP5K2 (PPIP5K2^KD^) in complex with natural substrate within the catalytic site (Wang et al., 2012). However, the architecture of the active site exhibits substantial geometric and electrostatic constraints that raise challenges for the design of an effective yet specific inhibitor.

In the current study, we set out to prepare substrate analogs that might modify PPIP5K2 activity. The synthesis of analogs of diphosphoinositol polyphosphates presents particular technical challenges due to the reactive nature of the diphosphate group and the protected diphosphate intermediates (Best et al., 2010). The high negative charge density of these materials also presents purification problems (Capolicchio et al., 2013). Although several of the naturally occurring diphosphoinositol polyphosphates have been synthesized (Albert et al., 1997; Best et al., 2010; Wu et al., 2013; Capolicchio et al., 2013), the preparation of useful analogs has only recently been accomplished (Riley et al., 2012; Wu et al., 2013). In the latter studies, analogs of 5-PP-InsP_4_ and 5-PP-InsP_5_ were synthesized in which the diphosphate groups were replaced with metabolically stabilized phosphonoacetate (PA) or methylenebisphosphonate (PCP) groups. In the current study, we describe the synthesis of a series of diphosphoinositol polyphosphate analogs. We demonstrate how we used these reagents to gain insight into a previously described (Weaver et al., 2013) substrate-stimulated ATPase activity of PPIP5K2^KD^. These experiments also led us to uncover a second ligand-binding site in PPIP5K2^KD^ that performs an important aspect of the catalytic cycle by enhancing capture of substrate from the bulk phase.

## Results and Discussion

### Stimulation of the ATPase Activity of PPIP5K2^KD^ by 5-PA-InsP_5_ and 2-*O*-Bn-5-PA-InsP_4_

**Figure 2:**
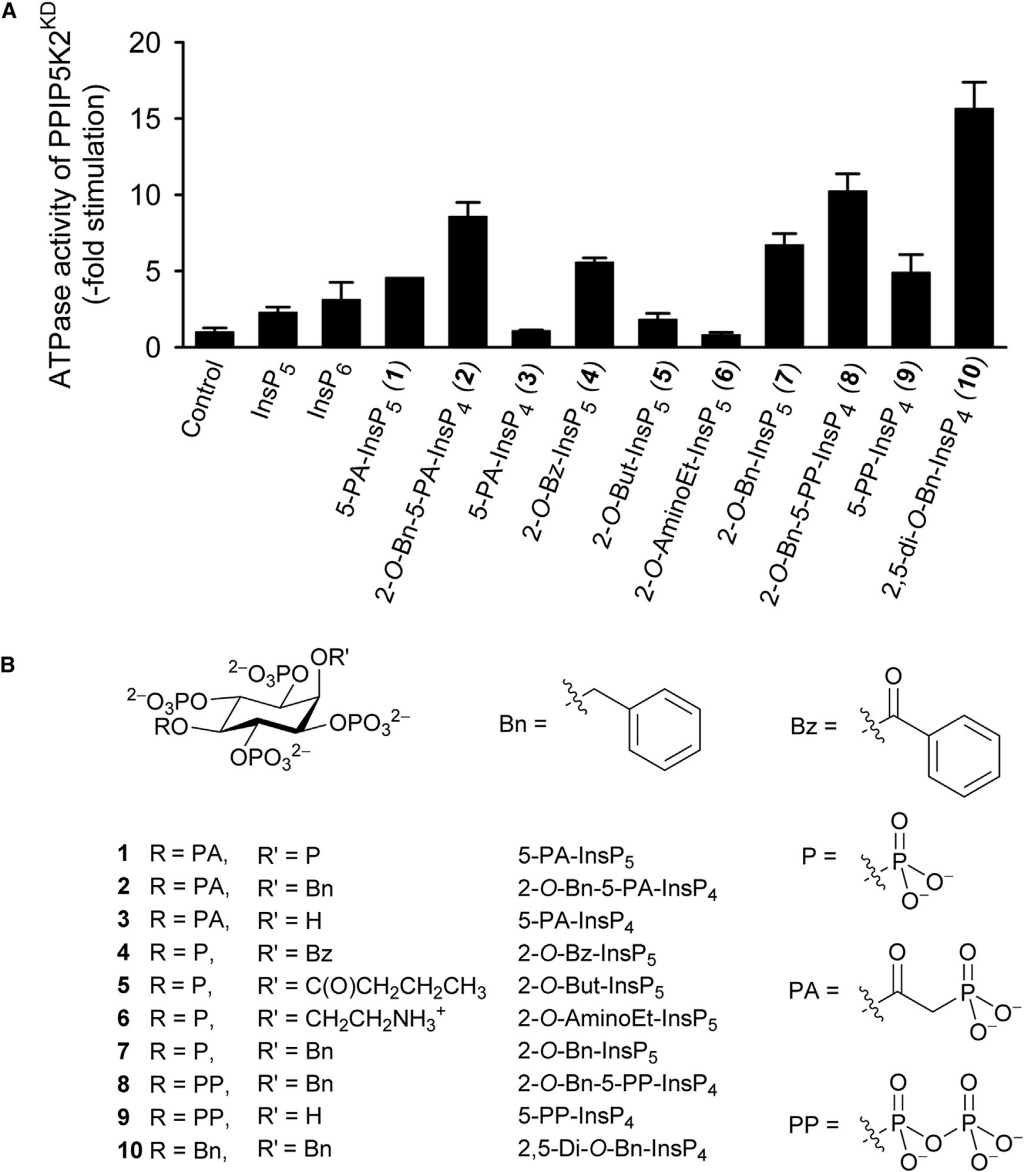
The Effects of Inositol Phosphates and Analogs upon ATPase Activity of PPIP5K2^KD^ (A) The effects of the indicated inositol phosphates and analogs (25 μM) upon ATPase activity of PPIP5K2^KD^. Data represent means and SEs from three experiments. Data for Ins(1,2,3,4,5)P_5_ (InsP_5_) and InsP_6_ are taken from Weaver et al., 2013. (B) The structures of the reagents that were used.

We recently reported that PPIP5K2^KD^ exhibits an unusual, non-productive, substrate-stimulated ATPase activity (e.g., we observed a 2- to 3-fold activation by 25 μM of either Ins(1,3,4,5,6)P_5_ or InsP_6_; Figure 2A; Weaver et al., 2013). We now report that 25 μM of either of two previously described analogs of diphosphoinositol polyphosphates (Riley et al., 2012) also stimulate ATP hydrolysis 5-fold by 5-*O*-α-phosphonoacetyl-*myo*-inositol 1,2,3,4,6-pentakisphosphate (5-PA-InsP_5_ [**1**]), and 9-fold by 2-*O*-benzyl-5-*O*-α-phosphonoacetyl*-myo*-inositol 1,3,4,6-tetrakisphosphate (2-*O*-Bn-5-PA-InsP_4_ [**2**]; Figures 2A and 2B). In view of the precise geometric and electrostatic specificity constraints within the active site (Wang et al., 2012), we did not anticipate that it could accommodate 2-*O*-Bn-5-PA-InsP_4_, which sports a bulky hydrophobic group. It was therefore unexpected that 2-*O*-Bn-5-PA-InsP_4_ should be a more effective activator of ATPase activity than the two natural substrates, InsP_6_ (Figure 2A) and PP-InsP_5_ (Weaver et al., 2013). We sought further information on this phenomenon.

### Design and Synthesis of Inositol Phosphate and Diphosphoinositol Phosphate Analogs

**Figure 3:**
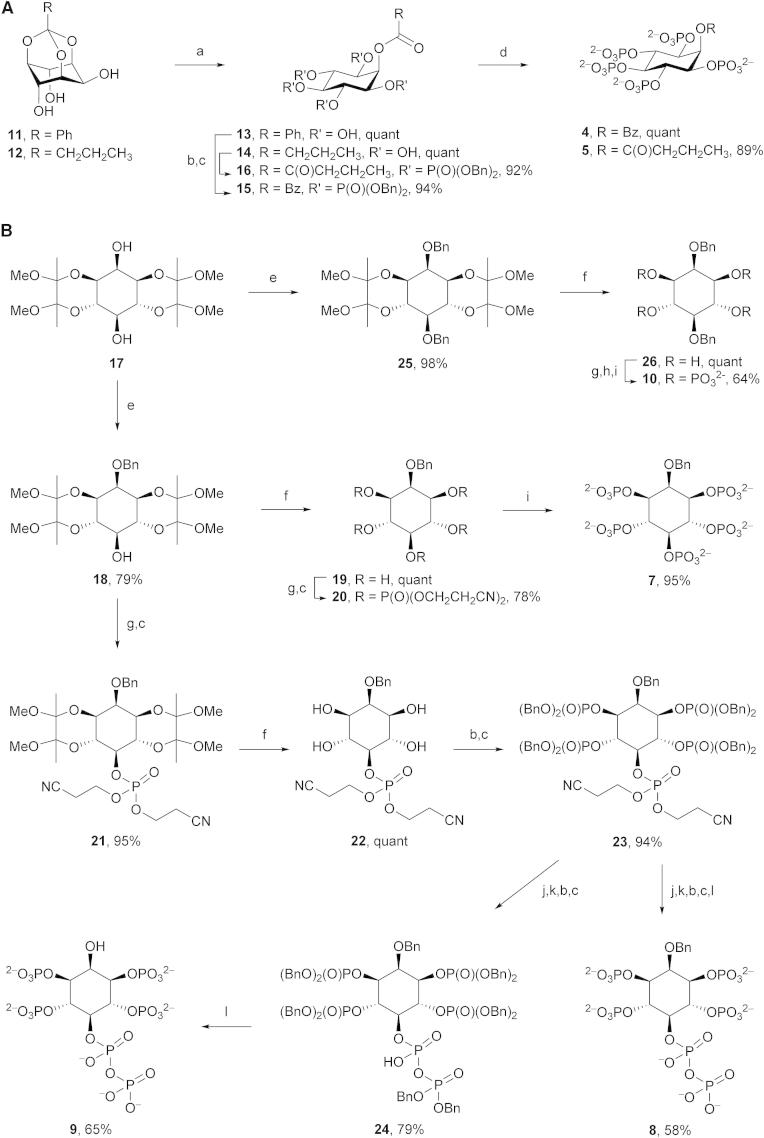
Chemical Syntheses (A) Syntheses of 2-*O*-Bz-InsP_5_ (**4**) and 2-*O*-But-InsP_5_ (**5**). (B) Syntheses of 2-*O*-Bn-InsP_5_(**7**), 2-*O*-Bn-5-PP-InsP_4_ (**8**), 5-PP-InsP_4_ (**9**), and 2,5-di-*O*-Bn-InsP_4_ (**10**). Reagents and conditions: (a) TFA:H_2_O, 10:1; (b) (BnO)_2_PN(^*i*^Pr)_2_, 5-phenyl-1-*H*-tetrazole, DCM; (c) *m*CPBA, −40°C to room temperature; (d) Pd(OH)_2_, H_2_, MeOH, H_2_O; (e) BnBr, NaH, DMF; (f) aq TFA (90%):DCM, 1:1; (g) (NCCH_2_CH_2_O)_2_PN(^*i*^Pr)_2_, 5-phenyl-1-*H*-tetrazole, DCM; (h) *t*BuOOH, −40°C to room temperature; (i) aq ammonia, 70°C; (j) DBU, BSTFA; (k) MeOH, TFA; (l) NaHCO_3_, Pd(OH)_2_, H_2_, ^t^BuOH, H_2_O.

To increase insight into the origin of ligand-stimulated ATPase activity of PPIP5K2^KD^, we explored the contributions of the benzyl (Bn) and α-phosphonoacetyl (PA) groups in 2-*O*-Bn-5-PA-InsP_4_ (**2**). Our approach was to synthesize a series of analogs (Figure 2B) that exchanged Bn for either OH, monophosphate, or alternative hydrophobes, whereas the PA group was replaced by either a monophosphate or a diphosphate. For analogs containing acyl groups at O-2, the appropriate synthetic precursors are orthoesters of *myo*-inositol, because acid hydrolysis of these compounds gives rise to 2-*O*-acyl esters with very high regioselectivity (Godage et al., 2006, 2013). Thus, 2-*O*-benzoyl-InsP_5_ (**4**) and 2-*O*-butanoyl-InsP_5_ (**5**; Figures 2B and 3A) were synthesized from *myo*-inositol orthobenzoate (**11**) and *myo*-inositol orthobutanoate (**12**), respectively. Acid hydrolysis of **11** and **12** (Godage et al., 2013) gave pentaols **13** and **14**, respectively, and subsequent phosphorylation and deprotection provided 2-*O*-benzoyl-InsP_5_ (**4**) and 2-*O*-butanoyl-InsP_5_ (**5**).

For analogs containing alkyl ethers or hydroxyl groups at C-2, synthetic routes proceeding from the symmetrical butane-2,3-diacetal (BDA) protected diol **17** are expedient, as in our recent synthesis of 2-*O*-(2-aminoethyl)-InsP_5_ (**6**; Riley et al., 2014). The remaining compounds **7**–**10** were also synthesized from diol **17** using a unified approach (Figures 2B and 3B). Thus, regioselective benzylation of the axial hydroxyl group of diol **17** gave 2-*O*-benzyl ether **18**, and removal of BDA protecting groups gave pentaol **19**. Phosphorylation of **19** afforded the symmetrical pentakisphosphate **20** and cleavage of phosphate protection gave 2-*O*-Bn-InsP_5_ (**7**) in 59% overall yield from **17**.

For the synthesis of 2-*O*-Bn-5-PP-InsP_4_ (**8**) and 5-PP-InsP_4_ (**9**), the C-5 hydroxyl group of **18** was first phosphitylated with bis(cyanoethyl)(*N*,*N*-diisopropylamino)phosphine, followed by oxidation to provide the protected monophosphate **21**. Removal of BDA groups followed by phosphorylation afforded the fully protected pentakisphosphate **23** in high yield (Figure 3B). We constructed the diphosphate group by modifying a recently described methodology (Capolicchio et al., 2013); compound **23** was treated with DBU (1,8-diazabicyclo[5.4.0]undec-7-ene) and BSTFA (*N*,*O*-bis(trimethylsilyl)trifluoroacetamide) to remove both cyanoethyl protecting groups on the 5-phosphate, followed by methanolysis of the temporary trimethylsilyl protection to give the phosphate monoester at the C-5 position. Phosphitylation then afforded a mixed P(III)-P(V) intermediate, which was oxidized to produce **24** (Figure 3B). We next prepared 2-*O*-Bn-5-PP-InsP_4_ (**8**) from crude **24** (which contained DBU), by hydrogenolysis over palladium hydroxide, which removed the benzyl esters from the phosphates; amines (either in DBU or in triethylamine; Riley et al., 2012) inhibit hydrogenolytic cleavage of the 2-*O*-benzyl ether. Note that 2-*O*-Bn-5-PP-InsP_4_ (**8**) is a synthetic analog of an inositol pyrophosphate that retains the actual diphosphate group.

Hydrogenolysis of purified **24** from which DBU was removed yielded 5-PP-InsP_4_ (**9**), in 51% overall yield from **23** (Figure 3B). This molecule is a naturally occurring diphosphoinositol polyphosphate that features an unphosphorylated 2-OH group (Figure 1); 5-PP-InsP_4_ has been implicated in telomere maintenance (York et al., 2005; Saiardi et al., 2005). Finally, for the synthesis of 2,5-di-*O*-Bn-InsP_4_ (**10**), diol **17** was benzylated followed by cleavage of BDA groups. Subsequent phosphorylation and deprotection of cyanoethyl phosphate esters gave (**10**) in 63% overall yield from **17** (Figure 3B).

### Stimulation of the ATPase Activity of PPIP5K2^KD^ by Synthetic Inositol Phosphate Analogs

As discussed above (see also Figure 2A), 2-*O*-Bn-5-PA-InsP_4_ (**2**) strongly activated nonproductive ATPase activity of PPIP5K2^KD^. The degree of stimulation was dramatically reduced when the latter’s benzyl group was replaced with a hydroxyl group to give 5-PA-InsP_4_ (**3**, the PA analog of 5-PP-InsP_4_; Riley et al., 2012; Figure 2). Furthermore, 5-PP-InsP_4_ (**9**) stimulated ATPase activity at about half the rate shown by 2-*O*-Bn-5-PP-InsP_4_ (**8**). Taken together, these data (Figure 2) lead to an unexpected conclusion that the ligand’s benzyl group makes an important contribution to its association with PPIP5K2^KD^.

In 2-*O*-Bn-5-PP-InsP_4_ (**8**), the natural and more negatively charged diphosphate (PP) group replaces the PA group at *O*-5 in 2-*O*-Bn-5-PA-InsP_4_ (**2**). We found that 2-*O*-Bn-5-PP-InsP_4_ (**8**) was slightly (20%) more effective at stimulating ATPase activity than was 2-*O*-Bn-5-PA-IP_4_ (**2**; Figure 2). Nevertheless, the PA group still contributes to efficacy more than a monophosphate group because 2-*O*-Bn-InsP_5_ (**7**) was less effective than was 2-*O*-Bn-5-PA-InsP_4_ (**2**; Figure 2).

Even in 2-*O*-Bn-InsP_5_ (**7**), the 2-*O*-benzyl ether has an enhancing effect, roughly doubling the rate of ATP hydrolysis that is induced by InsP_6_, which has a phosphate group at C-2 (Figure 2). To investigate the effect of hydrophobes other than benzyl at *O*-2, we compared 2-*O*-Bn-InsP_5_ (**7**) with 2-*O*-Bz-InsP_5_ (**4**) and 2-*O*-But-InsP_5_ (**5**). The change from the 2-*O*-benzyl ether to the structurally related 2-*O*-benzoyl ester led to only a minimal reduction in ATPase-stimulating activity, while substitution with a 2-*O*-butanoyl ester gave a sharp reduction in activity (Figure 2). Furthermore, the analog with a positively charged aminoethyl group at O-2 in (2-*O*-AminoEt-InsP_5_ [**6**]) did not stimulate ATPase activity (Figure 2).

We next tested one further analog in which the diphosphate group in 2-*O*-Bn-5-PP-InsP_4_ (**8**) was replaced with a second benzyl group (i.e., 2,5-di-*O*-Bn-InsP_4_; **10**). This compound elicited the highest degree of ATPase activity among all of the analogs that we have synthesized (Figure 2). This was initially a puzzling conclusion, as structural considerations indicate that 2,5-di-*O*-Bn-InsP_4_ **(10**) would at best be a poor ligand for the active site.

**Figure 4:**
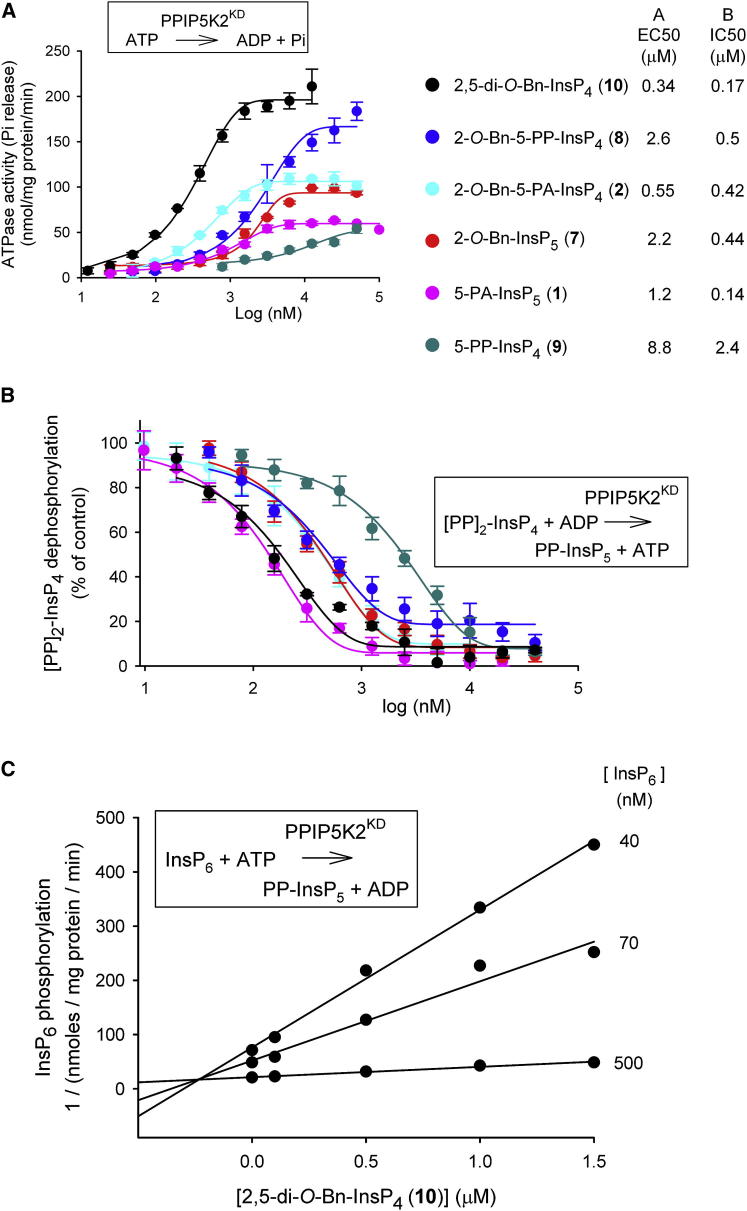
Effects of Inositol Phosphate Analogs upon PPIP5K2^KD^ Activities: ATPase, 1,5-[PP]_2_-InsP_4_ Dephosphorylation and InsP_6_ Phosphorylation (A and B) Dose-response curves are shown for the indicated inositol phosphate analogs and their stimulation of ATPase activities (A), and their inhibition of ADP-driven dephosphorylation of 100 nM [PP]_2_-InsP_4_ (B). Error bars represent SEs (n = 3). The EC_50_ and IC_50_ values for the molecules tested are given in the key to the right of (A) and (B). (C) A Dixon plot of the inhibition of InsP_6_ kinase activity of PPIP5K2^KD^ by 2,5-di-*O*-Bn-InsP_4_ (**10**); the data are from a representative experiment (one of three), performed in duplicate. Insets illustrate the reaction being assayed.

Several of the compounds described above were selected for detailed dose-response curves; we used the six compounds that yielded the greatest stimulation of ATPase activity, with the exception of 2-*O*-Bz-InsP_5_ (**4**), which in any case showed similar efficacy to 2-*O*-Bn-InsP_5_ (**7**; Figure 4A). The most potent of this series of compounds was 2,5-di-*O*-Bn-InsP_4_ (**10**, half-maximal effective concentration [EC_50_] = 340 nM; Figure 4A). In general, the rank order of the EC_50_ values for these compounds approximated the rank order of their maximal effects, with the notable exception of 2-*O*-Bn-5-PP-InsP_4_ (**8**; Figure 4A).

### Inhibition of PPIP5K2^KD^ Catalytic Activity by Inositol Phosphate Analogs

The six compounds that were selected for the dose-response curves in the assays of ATP hydrolysis (Figure 4A) were now investigated for their effects upon diphosphoinositol polyphosphate metabolism by PPIP5K2^KD^ (Figure 4B). We used a high-throughput reverse-kinase assay that records ATP generated from ADP during the dephosphorylation of 100 nM 1,5-[PP]_2_-InsP_4_ (Riley et al., 2012; Weaver et al., 2013). Each of the tested analogs inhibited [PP]_2_-InsP_4_ metabolism by PPIP5K2^KD^ (Figure 4B). The two most potent of these analogs were 5-PA-InsP_5_ (**1**) and 2,5-di-*O*-Bn-InsP_4_ (**10**) (Figure 4B). The inhibitory effect of 5-PA-InsP_5_ (**1**) was not in itself surprising becasue we have already demonstrated it to be a PPIP5K substrate (Riley et al., 2012). However, the inhibition by 2,5-di-*O*-Bn-InsP_4_ (**10**) was more unexpected because due to its bulk, and its less negative charge compared to physiological substrates, we predict it to be a poor ligand for the active site of the highly specific PPIP5K2^KD^ (Wang et al., 2012). Nevertheless, its ability to inhibit reverse-kinase activity was directly confirmed by high-performance liquid chromatography (HPLC) analysis of [PP]_2_-[^3^H]InsP_4_ dephosphorylation (Figures S1A–S1C available online). We confirmed that 2,5-di-*O*-Bn-InsP_4_ (**10**) also inhibited PPIP5K2^KD^ in the “forward,” kinase direction using InsP_6_, a physiologically relevant substrate (Figures S1D and S1E). A Dixon plot demonstrated that inhibition by 2,5-di-*O*-Bn-InsP_4_ (**10**) was competitive in nature (K_i_ = 240 nM; Figure 4C), arguing that its actions are not allosteric in nature.

The rank order of potencies (as half-maximal inhibitory concentration [IC_50_] values) with which this group of molecules inhibited [PP]_2_-InsP_4_ dephosphorylation by PPIP5K2^KD^ (Figure 4B) is different in two key respects from the rank-order of efficacy (EC_50_ values) for their separate stimulation of ATPase activity (Figures 4A and 4B). First, 2-*O*-Bn-InsP_5_ (**7**) is 4-fold less potent than 2-*O*-Bn-5-PA-InsP_4_ (**2**) at stimulating ATPase activity (Figure 4A), whereas the two analogs are equally efficient at inhibiting [PP]_2_-InsP_4_ metabolism (Figure 4B). Second, 5-PA-InsP_5_ (**1**), the weakest activator of ATPase (Figure 4A), is the most potent inhibitor of [PP]_2_-InsP_4_ dephosphorylation (Figure 4B). These are observations that suggest there is some uncoupling of inositol phosphate turnover from the ATPase activity.

### Structural Analysis Reveals that PPIP5K2^KD^ Has Two Adjacent Ligand-Binding Sites

**Figure 5:**
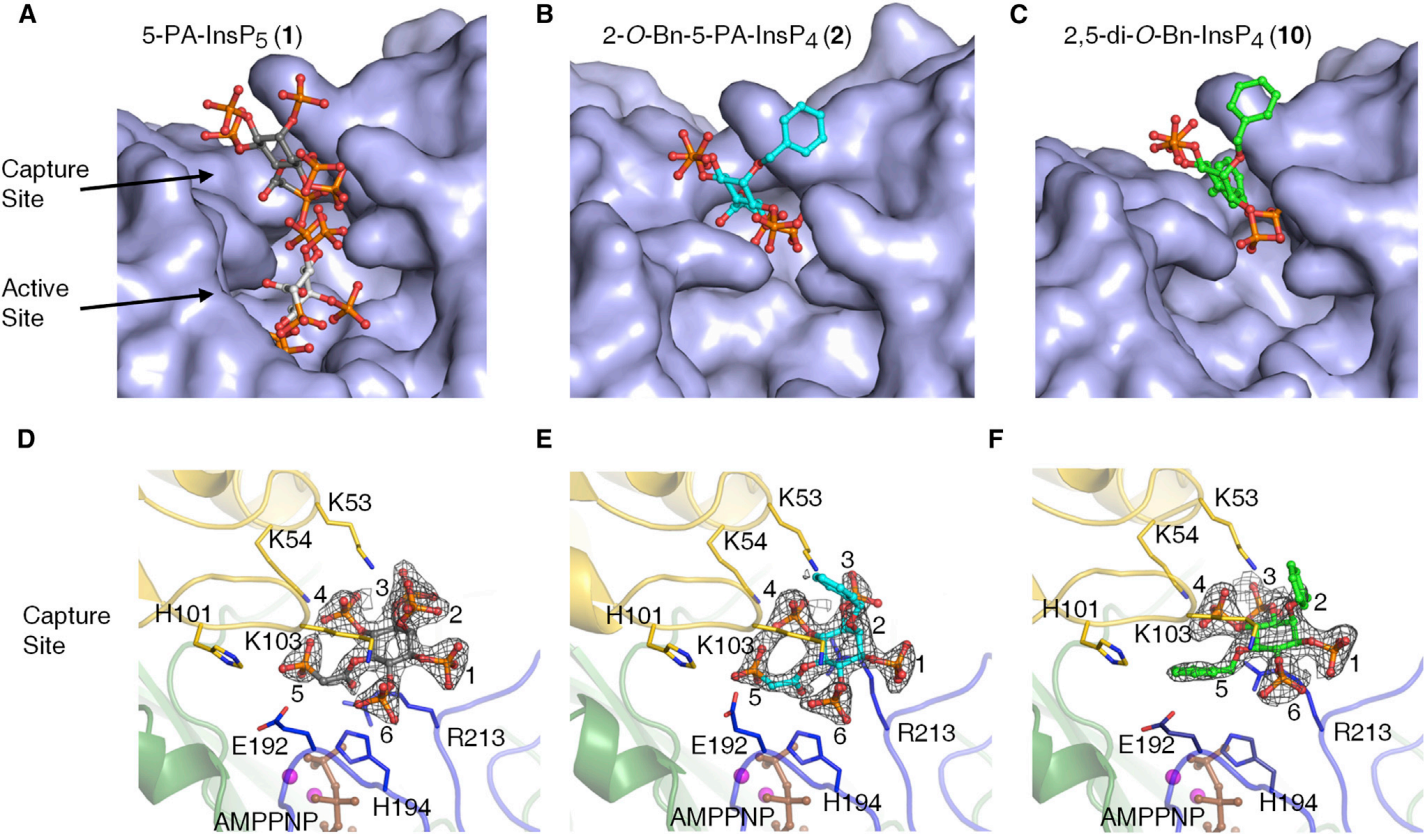
X-Ray Analysis of Inositol Phosphate Analogs Bound to PPIP5K2^KD^ Uncovers Two Ligand-Binding Sites X-ray analysis was performed after we soaked crystals of PPIP5K2^KD^ with either 10 mM of 5-PA-InsP_5_ (**1**), 5 mM of 2-*O*-Bn-5-PA-InsP_4_ (**2**), or 10 mM of 2,5-di-*O*-Bn-InsP_4_ (**10**). (A–C) Surface representations of ligand binding, with the analogs shown as stick-and-ball models. Atoms are blue for nitrogen; red for oxygen; orange for phosphorus; and gray, cyan or green for carbon. (D–F) The corresponding depictions of the protein as a ribbon diagram, with some key residues as stick models and numbered. For increased clarity, (D) only shows the 5-PA-InsP_5_ (**1**) that binds to the capture site (see the text for the functional rationalization of this site). The carbons around the inositol ring are numbered. AMPPNP is brown and magnesium atoms are magenta. Refined 2Fo-Fc maps are contoured at 1.0 σ and are shown in gray mesh.

We have previously published an atomic-level description of both 5-PP-InsP_5_ and InsP_6_ substrates bound into the catalytic pocket of PPIP5K2^KD^ (Wang et al., 2012). In a separate study, we (Riley et al., 2012) soaked 2 mM 5-PA-InsP_5_ (**1**) into crystals of PPIP5K2^KD^, and reported that this ligand occupies the active site in the same orientation as the natural substrate. At the time of that earlier study, we contoured the simulated annealing omit map at 2 σ, and observed some additional but uninterpretable electron density at the entrance of the active site (data not shown). For the current study, we increased the soaking concentration of 5-PA-InsP_5_ (**1**) to 10 mM, and have now unequivocally detected an additional ligand-binding site, at 1.7 Å resolution, that is located near the surface of PPIP5K2^KD^, at the entrance to the catalytic center (Figures 5A and 5D; Table S1). This surface-mounted ligand-binding site is too remote from ATP to permit ligand phosphorylation. It should be noted that both this site and the catalytic pocket cannot be occupied simultaneously due to steric clashing (Figures 5A and S2A). That is, these particular structural data arise from a mixture of crystal complexes in which 5-PA-InsP_5_ (**1**) is separately bound to either of the two sites.

The stimulation of ATPase activity of PPIP5K2^KD^ by either 2-*O*-Bn-5-PA-InsP_4_ (**2**) or 2,5-di-*O*-Bn-InsP_4_ (**10**) indicates that these compounds also interact with PPIP5K2^KD^, so we soaked 5–10 mM of either 2-*O*-Bn-5-PA-InsP_4_ (**2**) or 2,5-di-*O*-Bn-InsP_4_ (**10**) into the PPIP5K2^KD^ crystals. X-ray analysis revealed that both of these analogs were exclusively associated with the second ligand-binding site (Figures 5B, 5C, 5E, and 5F; Table S1). Neither of these analogs was found to occupy the catalytic site. These data suggest that the stimulation of ATPase activity by either natural substrates or their analogs is associated with their occupation of the second binding site, not the catalytic site. That explanation is in turn consistent with other data (Figures 4A and 4B, and see above) that suggest ligand-stimulated ATPase activity is uncoupled from the inositol phosphate kinase activity.

**Figure 6:**
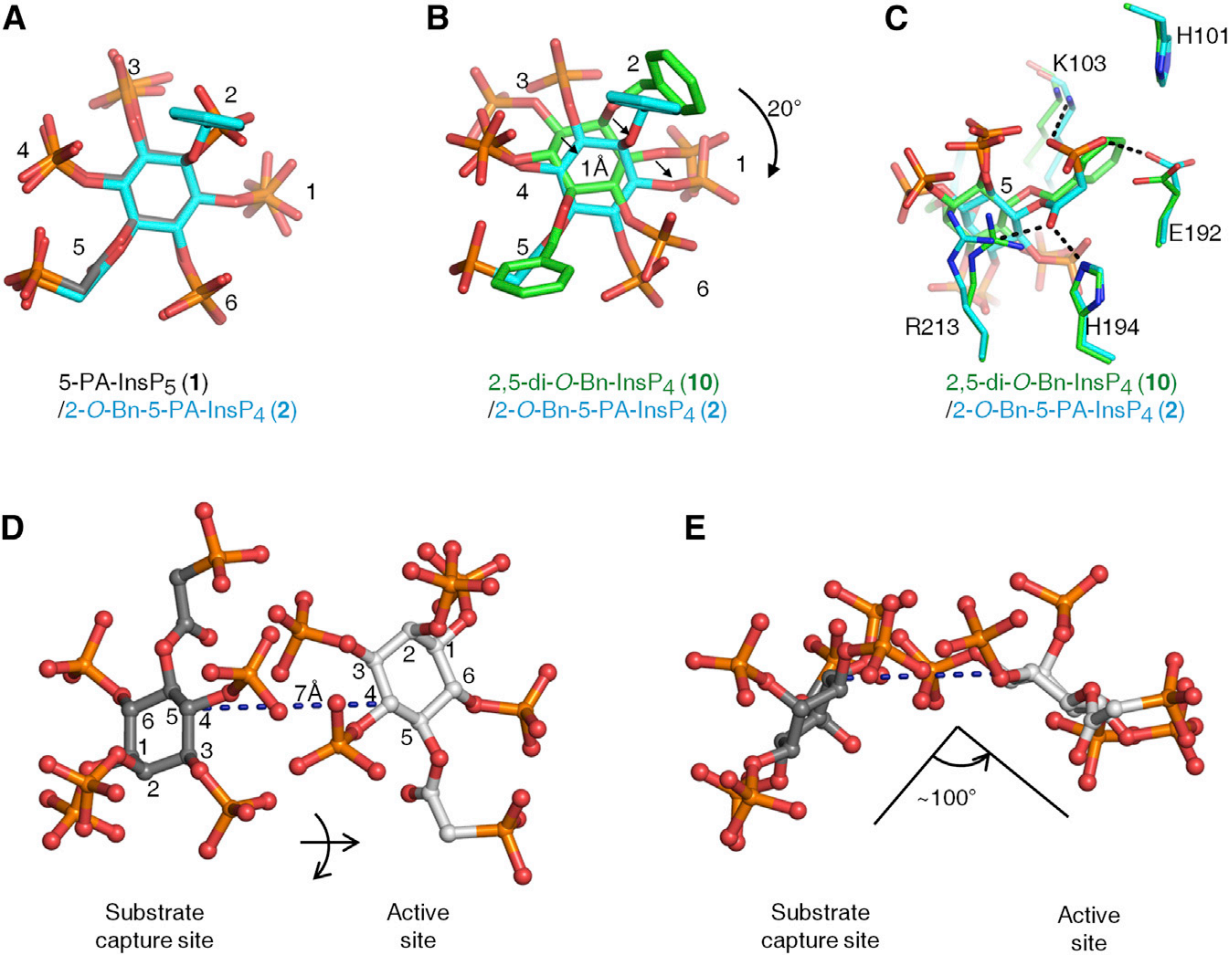
Relative Orientations of Inositol Phosphate Analogs in the Second Ligand-Binding Site of PPIP5K2^KD^ Data for ligand orientation in the second binding site were obtained as described in Figure 5. Analogs are depicted as stick models. Atoms are blue for nitrogen, red for oxygen, and orange for phosphorus. (A) Superimposition of 5-PA-InsP_5_ (**1**; carbons in gray) and 2-*O*-Bn-5-PA-InsP_4_ (**2**; carbons in cyan). (B and C) Superimposition of 2,5-di-*O*-Bn-InsP_4_ (**10**; carbons in green) and 2-*O*-Bn-5-PA-InsP_4_ (**2**; carbons in cyan). Arrows in (B) emphasize spatial separation, and (C) includes details of ligand interactions with neighboring amino acid residues. (D and E) illustrate that transfer of 5-PA-InsP_5_ (**1**) between the two sites involves a lateral migration of 7 Å, a ring flip, and a rotation of ∼100°. Atoms are blue for nitrogen, red for oxygen, orange for phosphorus, and gray for carbon. The inositol ring is numbered.

The architecture of this second ligand-binding site is represented by a deep cleft, which is walled on one side by K53, K54, and K103. The opposite face is formed from R213, and a loop created from residues E192 to H194 (Figure 5). In this binding site, the shared groups in 5-PA-InsP_5_ (**1**) and 2-*O*-Bn-5-PA-InsP_4_ (**2**) exhibit almost identical conformations (Figure 6A). As for 2,5-di-*O*-Bn-InsP_4_ (**10**) this molecule is rotated ∼20° clockwise (Figure 6B). For both 2-*O*-Bn-5-PA-InsP_4_ (**2**) and 2,5-di-*O*-Bn-InsP_4_ (**10**), their phosphate groups at C-3 and C-4 would clash with the positions that the C-4 and C-3 phosphates of InsP_6_/5-PP-InsP_5_ normally occupy in the active site (Figures S2A–S2C). This phenomenon presumably contributes to the potency of both 2-*O*-Bn-5-PA-InsP_4_ (**2**) and 2,5-di-*O*-Bn-InsP_4_ (**10**) as inhibitors of inositol phosphate turnover by PPIP5K2^KD^ (Figures 4B and 4C). These structural considerations explain how both compounds can inhibit inositol phosphate kinase activity without occupying the catalytic site.

The association of 2-*O*-Bn-5-PA-InsP_4_ (**2**) and 2,5-di-*O*-Bn-InsP_4_ (**10**) with the second binding site is facilitated by their phosphate groups at C-3 and C-4 making multiple electrostatic interactions with K53, K54, and R213 (Figures 5, S2E, and S2F). The terminal phosphonate of 2-*O*-Bn-5-PA-InsP_4_ (**2**) also forms polar contacts with the backbone of K103 and the H194 side chains (Figures 5 and S2E). Additionally, the 5-*O*-benzyl group in 2,5-di-*O*-Bn-InsP_4_ (**10**) has van der Waals interactions with the side chains of H101 and E192 (Figures 5 and S2F). The 2-*O*-benzyl group in this analog is disordered, indicative of its mobility. Nevertheless, our catalytic data (Figure 4) indicate that this benzyl group makes a significant contribution to ligand potency.

The data that we have obtained allow important conclusions to be drawn concerning the likely significance of the second binding site. For example, we can exclude the possibility that, as is the case with certain enzymes (Reed et al., 2010), the role of the second, noncatalytic substrate-binding site is to regulate enzyme activity by substrate inhibition; PPIP5K2^KD^ does not exhibit that property (Weaver et al., 2013). Other enzymes use noncatalytic ligand-binding sites to promote enzyme activation allosterically (Grant, 2012). That also seems unlikely to be the case for PPIP5K2^KD^, because our inositol phosphate analogs do not enhance kinase activity, but inhibit it instead (Figure 4B; Riley et al., 2012). The nature of the inhibition was competitive (Figure 4C), further arguing against an allosteric effect. Instead, the proximity to the active site of the second ligand-binding site (Figure 4) suggests that the latter plays a role in catalysis of natural substrates, by facilitating their capture from the bulk phase. A precedent for such a phenomenon has been reported for microbial anthranilate phosphoribosyltransferases (Castell et al., 2013; Marino et al., 2006); anthranilate substrate is first captured at a surface-mounted binding-site, before delivery to a proximal catalytic site (Castell et al., 2013). A similar substrate transfer is feasible in PPIP5K2^KD^, and our structural data provide some atomic-level insight into such a phenomenon. For example, we presume that delivery of natural substrate is efficient, so that substrate only occupies the capture site transiently; this may explain why we could not detect substrate in the capture site in our crystal structures. We found that such substrate transfer within PPIP5K2^KD^ requires that the inositol ring be flipped and rotated by ∼100° (Figures 6D and 6E). This motion is presumably facilitated by the flexible amino-acid side chains that comprise the active site (Wang et al., 2012). Such conformational dynamics can reinforce catalytic specificity (Herschlag, 1988), which is indeed a notable feature of PPIP5K2^KD^ (Wang et al., 2012).

### Site-Directed Mutagenesis of the Substrate Capture Site

**Figure 7:**
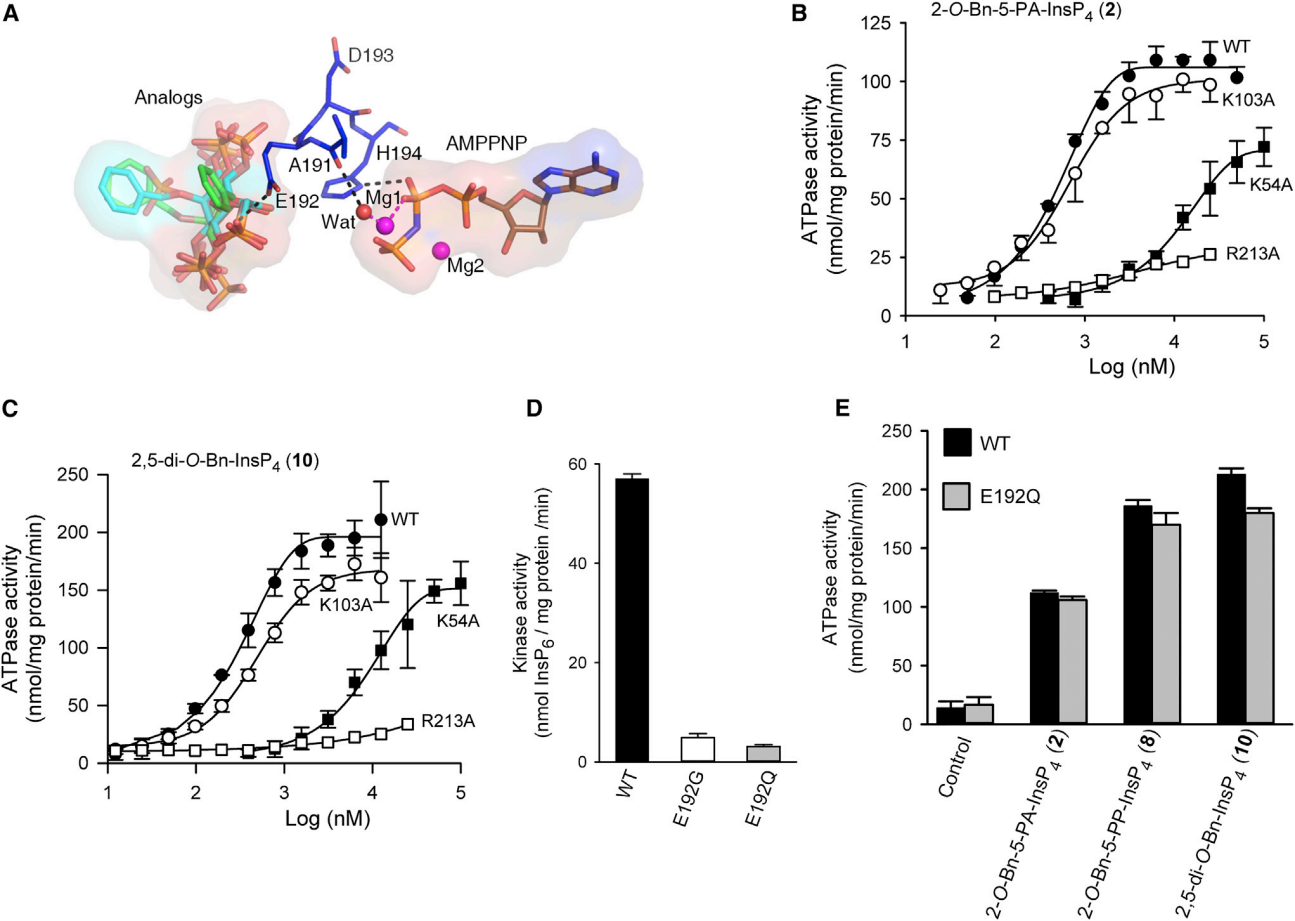
Impact on the Catalytic Properties of PPIP5K2^KD^ Following Mutation of K54, K103, E192, and K213 (A) The spatial separation of AMPPNP from the PPIP5K2^KD^ substrate capture site. Protein residues are shown as stick models. AMPPNP, and inositol phosphate analogs 5-PA-InsP_5_ (**1**), 2-*O*-Bn-5-PA-InsP_4_ (**2**), and 2,5-di-*O*-Bn-InsP_4_ (**10**) and all are shown as stick models. Atoms are blue for nitrogen; red for oxygen; orange for phosphorus; and gray, cyan, or green for carbon. Hydrogen bonds are shown as black dashed lines. (B and C) Dose-response curves for the effects of 2-*O*-Bn-5-PA-InsP_4_ (**2**) and 2,5-di-*O*-Bn-InsP_4_ (**10**) upon the ATPase activities of wild-type PPIP5K2^KD^ (closed circles; data are taken from Figure 2A) and the following mutants: K54A mutant (closed squares), K103A (open circles), and R213A (open squares). (D) The InsP_6_ kinase activities of wild-type, E192G, and E192Q mutants of PPIP5K2^KD^, determined with 5 μM substrate. (E) The ATPase activities of wild-type and E192Q mutants of PPIP5K2^KD^ obtained in the presence of the indicated analogs, at concentrations of 25 μM. Error bars represent SEs from three experiments (error bars are not shown when they are smaller than the symbol).

Mutagenesis offers a valuable means of pursuing conclusions drawn from structural analysis. The loop that is formed from residues A191 to H194 screens the catalytic site from ligands that are associated with the second binding site (Figures 5, 7A, and S3). The influence of the carbonyl oxygen of A191 may be indirect, through a hydrogen bond with a water molecule that in turn coordinates with an Mg^2+^ ion that interacts with the nucleotide’s β-phosphate moiety (Figure 7A). The role of H194 seems more direct; it can form a hydrogen bond with the oxygen atom of the nucleotide’s β-phosphate (Figures 7A and S3). This could stabilize the transition state, or have some other catalytic role. These interactions could be relevant to both kinase and ATPase activities of PPIP5K2^KD^. Consistent with these ideas, an H194A mutant did not exhibit any detectable ATPase activity, either in the presence or absence of 2-*O*-Bn-5-PA-InsP_4_ (**2**) or 2,5-di-*O*-Bn-InsP_4_ (**10**), and its inositol phosphate kinase activity was reduced 80-fold compared to that of the wild-type enzyme (data not shown).

As discussed above, our structural and biochemical data led us to hypothesize that the stimulation of ATPase activity by either natural substrates or their analogs is associated with their occupation of the substrate capture site, not the catalytic site. We attempted to consolidate this idea by selecting for mutation residues that might separate ATPase activity from inositol phosphate kinase activity. For example, our X-ray data (Figure 5) indicate that K54 and R213 interact with ligand at the substrate capture site. However, previous mutagenic work has shown that both K54 and R213 also contribute directly to inositol phosphate kinase activity (Wang et al., 2012). Nevertheless, we did observe that K54A and R213A mutants exhibited a substantially impaired degree of stimulation of ATPase activity by either 2-*O*-Bn-5-PA-InsP_4_ (**2**) or 2,5-di-*O*-Bn-InsP_4_ (**10**) (Figures 7B and 7C). These mutagenic data are consistent with the idea that K54 and R213 participate in substrate capture.

The side chain of K103 is also suggested by our structural data to interact with ligand that is bound to the capture site (Figures 5 and S2). Nevertheless, a K103A mutant showed only a slight reduction in 2-*O*-Bn-5-PA-InsP_4_ (**2**)-activated and 2,5-di-*O*-Bn-InsP_4_ (**10**)-activated ATPase activity, compared to wild-type enzyme (Figures 7B and 7C). Likewise, the InsP_6_ kinase activity of the K103A mutant (71 ± 4 nmol/mg protein/min, n = 3) was similar to that of wild-type enzyme (61 ± 4 nmol/mg protein/min).

We also mutated E192. This residue is present in the loop between the two ligand-binding sites (Figure 7A). It is too distant from ATP and the catalytic site to influence either directly, and electrostatic repulsion would prevent it from interacting with negatively charged groups on substrates located in the second binding site. Nevertheless, E192 is evolutionarily conserved in PPIP5Ks from mammals to yeast (not shown), suggestive of functional significance. We investigated the significance of E192 by preparing E192G and E192Q mutations that we posited would eliminate electrostatic repulsion between the amino acid side chain and phosphorylated ligands, enhancing ligand binding to the substrate capture site. We further hypothesized that such an effect might reduce the efficiency of transfer of substrate to the catalytic site, which in turn would lead to a reduction in inositol phosphate kinase activity. Indeed, we found that these E192G and E192Q mutations reduced the rate of InsP_6_ phosphorylation by 12- and 18-fold respectively, compared to wild-type PIP5K2^KD^ (Figure 7D). It was of further note that neither of these particular mutations altered the rate of nonproductive, ligand-stimulated ATPase activity elicited by either 2-*O*-Bn-5-PA-InsP_4_ (**2**), 2-*O*-Bn-5-PP-InsP_4_ (**8**), or 2,5-di-*O*-Bn-InsP_4_ (**10**; Figure 7E), confirming the uncoupling of this aspect of PPIP5K2^KD^ activity from its kinase activity.

We have shown that 2,5-di-*O*-Bn-InsP_4_ (**10**) binds to a substrate-capture site on the surface of PPIP5K2^KD^ (Figure 5), thereby inhibiting the enzyme’s catalytic activity (Figure 4). This particular ligand-binding site may be exploitable as a pharmacological target. We therefore examined the specificity of 2,5-di-*O*-Bn-InsP_4_ (**10**) by investigating if it interacted with IP6K2, a member of a different class of kinases that also phosphorylate InsP_6_ and synthesize diphosphoinositol polyphosphates (see Figure 1). Because the affinity of IP6K2 for InsP_6_ (430 nM; Saiardi et al., 2000) is very similar to that for InsP_6_ phosphorylation by PPIP5K2^KD^ (390 nM; Weaver et al., 2013), an identical substrate concentration ([InsP_6_] = 500 nM) was used to assay both enzymes. As shown in Figure 4C, 2,5-di-*O*-Bn-InsP_4_ (**10**) inhibits PPIP5K2^KD^ with an IC_50_ value of 1 μM (Figure 4C). This analog was found to be a much weaker inhibitor of IP6K2 activity (IC_50_ = 63 μM; data not shown). Thus, 2,5-di-*O*-Bn-InsP_4_ (**10**) may be a useful lead molecule for future development of a drug that can specifically inhibit PPIP5Ks and not IP6Ks, even though both groups of enzymes phosphorylate InsP_6_.

In conclusion, our studies have uncovered a “catch-and-pass” aspect to the catalytic cycle of PPIP5K2^KD^. Substrate (either InsP_6_ or 5-PP-InsP_5_) first associates with a ligand-binding site on the surface of the kinase. We propose that this phenomenon enhances kinase activity by improving substrate capture from the bulk phase; substrate is then delivered into the catalytic pocket. We are not aware of any other examples of a substrate-capture mechanism in a small-molecule kinase. Indeed, the only example that we have found in the literature for a dedicated substrate capture site on any enzyme is that observed for certain microbial anthranilate phosphoribosyl-transferases (Castell et al., 2013; Marino et al., 2006). Our structural, biochemical, and mutagenic data have also led us to conclude that the stimulation of ATPase activity of PPIP5K by inositol phosphate analogs is associated with their occupation of the substrate capture site, not the catalytic site. Moreover, the previously puzzling observation (Weaver et al., 2013) that a degree of nonproductive ATP hydrolysis is also stimulated by natural substrate, can now be rationalized as a consequence of its interaction with the capture site. Furthermore, the fact that InsP_6_-stimulated ATPase activity is abolished by an R213A mutation (Weaver et al., 2013) can now be viewed as resulting from an impairment to substrate occupation of the capture site.

Our atomic-level description of the substrate-capture site on PPIP5K2^KD^ indicates its structural determinants of specificity differ substantially from those of the catalytic site. We have further shown that synthesis of diphosphoinositol polyphosphates by PPIP5K2^KD^ is inhibited by ligands that bind to the capture site but not the catalytic site. These findings in turn raise the possibility that there may be cellular constituents that might inhibit catalytic activity by binding to this site. Finally, the substrate capture site offers a selective target for the purposes of rational drug design, including screening in silico (Kitchen et al., 2004), that is free from many of the usual specificity constraints within the catalytic site that typically complicate pharmacological targeting of small-molecule kinases. It may also be possible to design a ligand that occupies both sites.

## Significance

**PPIP5K1 and PPIP5K2 are small-molecule kinases that synthesize diphosphoinositol polyphosphates, which function at the interface of cell signaling and organismic homeostasis. Synthetic chemical modulators of cell-signaling enzymes such as the PPIP5Ks can provide valuable insight into catalytic mechanisms, they decipher the biological roles of the enzymes in situ, and they generate leads for therapeutic drug development. Substrate analogs offer one approach for the preparation of such chemical reagents. However, the architecture of the active site of PPIP5Ks, as revealed by recent X-ray analysis, has identified substantial geometric and electrostatic constraints that limit the options for designing a substrate analog that might be an effective modulator. In the current study, we describe the chemical synthesis of both the naturally occurring 5-PP-InsP**_**4**_ **and a family of inositol polyphosphate analogs that include molecules with hydrophobes at the 2- and/or 5-positions. Importantly, these molecules have led us to characterize a second, less constrained substrate-binding site on the surface of PPIP5K2, adjacent to the catalytic pocket. We provide an atomic-level description of this surface-mounted site and describe its role in the catalytic cycle in capturing substrate from the bulk phase. With the assistance of site-directed mutagenesis of this site, we show that its occupation is associated with an unusual, ligand-activated ATPase activity. This considerable amount of information had not been accessible with our experiments with natural substrates and represents a success for our chemical biology approach. In addition to adding to the repertoire of catalytic specializations of the PPIP5Ks, our structural and functional characterization of this ligand-binding site offers a promising target for drug development; to this end, 2,5-di-*O*-Bn-InsP**_**4**_ **is a significant lead compound.**

## Experimental Procedures

### Protein Expression, Purification, Crystallization, and Structure Determination

The kinase domain of human PPIP5K2 (PPIP5K2^KD^; residues 1–366) and the N-terminally truncated domain used for the crystallography studies (residues 41–366) were subcloned, expressed, and purified as before. The latter was crystallized by hanging drop vapor diffusion against a well buffer of 12% (w/v) PEG 3350, 20 mM MgCl_2_, 0.1 M HEPES, pH 7.0, 1 mM AMP-PNP, and 2 mM CdCl_2_ at 4°C. The crystals were then soaked with 5–10 mM compounds in a stabilizing buffer containing 22% (w/v) PEG 3350, 10 mM MgCl_2_, 0.1 M sodium acetate, pH 5.2 or 7.0, at 4°C for 3 days. Cryosolvent was prepared by adding 33% ethylene glycol into the soaking buffer. Diffraction data were collected using APS beamlines 22-BM and 22-ID. All data were processed with the program HKL2000. The structure was determined using rigid body and direct Fourier synthesis, and refined with the equivalent and expanded test sets. The structure was further manually rebuilt with COOT and refined with PHENIX and REFMAC from the CCP4 package. Ligand topology and parameter files were prepared using the PRODRG server. The molecular graphics representations were prepared with the program PyMol (Schrödinger). The 2D ligand-protein interaction diagrams were generated by LigPlot+.

### Enzyme Assays

HPLC was used to assay [^3^H]InsP_6_ phosphorylation by human PPIP5K2^KD^ or human IP6K2 in 100 μl buffer containing 50 mM KCl, 20 mM HEPES pH 7.0, 7 mM MgSO_4_, 5 mM ATP, and 1 mM EDTA (for IP6K2, [MgSO_4_] was 12 mM and [ATP] was 10 mM). The initial InsP_6_ concentration was either 500 nM, 70 nM, or 40 nM. Various concentrations of 2,5-di-*O*-Bn-InsP_4_ were present as indicated. Radioactivity was assessed using an in-line Flo1 detector. The ADP-driven dephosphorylation of 100 nM 1,5-[PP]_2_-InsP_4_ by PPIP5K2^KD^ was usually determined by a luminescence-based assay of ATP accumulation (Riley et al., 2012; Weaver et al., 2013). The IC_50_ values were determined using GraphPad Prism v6.02 (n ≥ 3). In some experiments, 1,5-[PP]_2_-[^3^H]InsP_4_ dephosphorylation by PPIP5K2^KD^ was assayed in 50 μl buffer containing 50 mM KCl, 20 mM HEPES pH 7.0, 7 mM MgSO_4_, 5 mM ADP, 1 mM EDTA. Reactions were then quenched, neutralized, and analyzed with HPLC using a Partisphere SAX column (Weaver et al., 2013); 1 ml fractions were collected for liquid scintillation spectrometry. The ATPase activity of PPIP5K2^KD^ (10–270 μg/ml) was assayed from Pi release following incubation at 37°C for 120–180 min in 20 μl reaction mixtures containing 20 mM Tris/HCl, pH 7.5, 10 mM ATP, 100 mM KCl, 0.1 mM EDTA, and 13 mM MgCl_2_.

### Statistical Analysis

In the figures, error bars represent SEs from three experiments.

## Author Contributions

H.W., H.Y.G., A.M.R., J.D.W., and S.B.S. carried out the experiments; S.B.S. and B.V.L.P. designed and coordinated the research; and all authors provided input during writing of the paper.

## Accession Numbers

The Protein Data Bank accession numbers for the protein/ligand structures reported in this paper are 4NZM, 4NZN, and 4NZO.

## Supplemental Information

Document S1. Supplemental Experimental Procedures, Figures S1–S3, and Table S1

Document S2. Article plus Supplemental Information

## References

[bib1] Albert C., Safrany S.T., Bembenek M.E., Reddy K.M., Reddy K.K., Falck J.R., Bröcker M., Shears S.B., Mayr G.W. (1997). Biological variability in the structures of diphosphoinositol polyphosphates in *Dictyostelium discoideum* and mammalian cells. Biochem. J..

[bib2] Benjamin D., Colombi M., Moroni C., Hall M.N. (2011). Rapamycin passes the torch: a new generation of mTOR inhibitors. Nat. Rev. Drug Discov..

[bib3] Best M.D., Zhang H., Prestwich G.D. (2010). Inositol polyphosphates, diphosphoinositol polyphosphates and phosphatidylinositol polyphosphate lipids: structure, synthesis, and development of probes for studying biological activity. Nat. Prod. Rep..

[bib4] Capolicchio S., Thakor D.T., Linden A., Jessen H.J. (2013). Synthesis of unsymmetric diphospho-inositol polyphosphates. Angew. Chem. Int. Ed. Engl..

[bib5] Castell A., Short F.L., Evans G.L., Cookson T.V., Bulloch E.M., Joseph D.D., Lee C.E., Parker E.J., Baker E.N., Lott J.S. (2013). The substrate capture mechanism of Mycobacterium tuberculosis anthranilate phosphoribosyltransferase provides a mode for inhibition. Biochemistry.

[bib6] Chakraborty A., Koldobskiy M.A., Bello N.T., Maxwell M., Potter J.J., Juluri K.R., Maag D., Kim S., Huang A.S., Dailey M.J. (2010). Inositol pyrophosphates inhibit Akt signaling, thereby regulating insulin sensitivity and weight gain. Cell.

[bib7] Choi K., Mollapour E., Shears S.B. (2005). Signal transduction during environmental stress: InsP_8_ operates within highly restricted contexts. Cell. Signal..

[bib8] Draskovic P., Saiardi A., Bhandari R., Burton A., Ilc G., Kovacevic M., Snyder S.H., Podobnik M. (2008). Inositol hexakisphosphate kinase products contain diphosphate and triphosphate groups. Chem. Biol..

[bib9] Godage H.Y., Riley A.M., Woodman T.J., Potter B.V.L. (2006). Regioselective hydrolysis of *myo*-inositol 1,3,5-orthobenzoate via a 1,2-bridged 2′-phenyl-1′,3′-dioxolan-2′-ylium ion provides a rapid route to the anticancer agent Ins(1,3,4,5,6)P5. Chem. Commun. (Camb.).

[bib10] Godage H.Y., Riley A.M., Woodman T.J., Thomas M.P., Mahon M.F., Potter B.V.L. (2013). Regioselective opening of *myo*-inositol orthoesters: mechanism and synthetic utility. J. Org. Chem..

[bib11] Gokhale N.A., Zaremba A., Janoshazi A.K., Weaver J.D., Shears S.B. (2013). PPIP5K1 modulates ligand competition between diphosphoinositol polyphosphates and PtdIns(3,4,5)P_3_ for polyphosphoinositide-binding domains. Biochem. J..

[bib12] Grant G.A. (2012). Kinetic evidence of a noncatalytic substrate binding site that regulates activity in *Legionella pneumophila* L-serine dehydratase. Biochemistry.

[bib13] Herschlag D. (1988). The role of induced fit and conformational changes in specificity and catalysis. Bioorg. Chem..

[bib14] Illies C., Gromada J., Fiume R., Leibiger B., Yu J., Juhl K., Yang S.-N., Barma D.K., Falck J.R., Saiardi A. (2007). Inositol pyrophosphates determine exocytic capacity. Science.

[bib15] Kitchen D.B., Decornez H., Furr J.R., Bajorath J. (2004). Docking and scoring in virtual screening for drug discovery: methods and applications. Nat. Rev. Drug Discov..

[bib16] Marino M., Deuss M., Svergun D.I., Konarev P.V., Sterner R., Mayans O. (2006). Structural and mutational analysis of substrate complexation by anthranilate phosphoribosyltransferase from *Sulfolobus solfataricus*. J. Biol. Chem..

[bib17] Pulloor N.K., Nair S., Kostic A.D., Bist P., Weaver J.D., Riley A.M., Tyagi R., Uchil P.D., York J.D., Snyder S.H. (2014). Human genome-wide RNAi screen identifies an essential role for inositol pyrophosphates in Type-I interferon response. PLoS Pathog..

[bib18] Reed M.C., Lieb A., Nijhout H.F. (2010). The biological significance of substrate inhibition: a mechanism with diverse functions. Bioessays.

[bib19] Riley A.M., Wang H., Weaver J.D., Shears S.B., Potter B.V.L. (2012). First synthetic analogues of diphosphoinositol polyphosphates: interaction with PP-InsP5 kinase. Chem. Commun. (Camb.).

[bib20] Riley A.M., Windhorst S., Lin H.-Y., Potter B.V.L. (2014). Cellular internalisation of an inositol phosphate visualised by using fluorescent InsP5. ChemBioChem.

[bib21] Saiardi A., Caffrey J.J., Snyder S.H., Shears S.B. (2000). The inositol hexakisphosphate kinase family. Catalytic flexibility and function in yeast vacuole biogenesis. J. Biol. Chem..

[bib22] Saiardi A., Resnick A.C., Snowman A.M., Wendland B., Snyder S.H. (2005). Inositol pyrophosphates regulate cell death and telomere length via PI3K-related protein kinases. Proc. Natl. Acad. Sci. USA.

[bib23] Shears S.B. (2009). Diphosphoinositol polyphosphates: metabolic messengers?. Mol. Pharmacol..

[bib24] Szijgyarto Z., Garedew A., Azevedo C., Saiardi A. (2011). Influence of inositol pyrophosphates on cellular energy dynamics. Science.

[bib25] Thomas M.P., Potter B.V.L. (2014). The enzymes of human diphosphoinositol polyphosphate metabolism. FEBS J..

[bib26] Wang H., Falck J.R., Hall T.M., Shears S.B. (2012). Structural basis for an inositol pyrophosphate kinase surmounting phosphate crowding. Nat. Chem. Biol..

[bib27] Weaver J.D., Wang H., Shears S.B. (2013). The kinetic properties of a human PPIP5K reveal that its kinase activities are protected against the consequences of a deteriorating cellular bioenergetic environment. Biosci. Rep..

[bib28] Wu M., Dul B.E., Trevisan A.J., Fiedler D. (2013). Synthesis and characterization of non-hydrolysable diphosphoinositol polyphosphate second messengers. Chem Sci.

[bib29] York S.J., Armbruster B.N., Greenwell P., Petes T.D., York J.D. (2005). Inositol diphosphate signaling regulates telomere length. J. Biol. Chem..

