# Supplementary material for: Synthetic Inositol Phosphate Analogs Reveal that PPIP5K2 Has a Surface-Mounted Substrate Capture Site that Is a Target for Drug Discovery
Source: Chem Biol. 2014 May 22;21(5):689–99. doi: 10.1016/j.chembiol.2014.03.009 (PMC4085797; doi:10.1016/j.chembiol.2014.03.009)

Chemistry & Biology, Volume *21*

Supplemental Information

Synthetic Inositol Phosphate Analogs Reveal that PPIP5K2 Has a Surface-Mounted Substrate Capture Site that Is a Target for Drug Discovery

Huanchen Wang, Himali Y. Godage, Andrew M. Riley, Jeremy D. Weaver, Stephen B. Shears, and Barry V.L. Potter

Supplemental Information

Synthetic inositol phosphate analogues in complex with PPIP5K2 uncover a substrate capture site as a target for drug discovery.

Huanchen Wang [1, 3], Himali Y. Godage [2, 3], Andrew M. Riley [2], Jeremy D. Weaver [1], Stephen B. Shears [1] and Barry V. L. Potter [2].

[1] Inositol Signaling Group, Laboratory of Signal Transduction, National Institute of Environmental Health Sciences, National Institutes of Health, Research Triangle Park, North Carolina, NC27709, USA.

[2] Wolfson Laboratory of Medicinal Chemistry, Department of Pharmacy and Pharmacology, University of Bath, BA2 7AY, UK.

[3] These authors contributed equally to this work.

Correspondence:

shears@niehs.nih.gov (SBS); b.v.l.potter@bath.ac.uk (BVLP)

Table of Contents

Chemical Synthesis: General Chemistry Methods	3
Experimental details for Compounds **16**, **5**, **18**, **19**, **20**, **7**, **21**, **22**, **23**, **8**, **24**, **9**, **25**, **26** and **10**	4-15
Protein Expression, Purification, Crystallization and Structure Determination	16
Mutagenesis	16
References	17
Table.S1, related to Figure 5: Data collection and structure refinement statistics	18
Figure Legends:	19-20
Figure S1, related to Figure 4: 2,5-Di-*O*-Bn-$InsP_4$ (10) inhibits both ADP-driven $[PP]_2$-[^{3}H]$InsP_4$ dephosphorylation and ATP-driven [^{3}H]$InsP_6$ phosphorylation by PPIP5K2^{KD}: HPLC analysis	21
Figure S2, related to Figure 6: Ligand occupation of the substrate capture site	22
Figure S3, related to Figure 7: Cross-eye stereo view of the spatial separation of AMP-PNP from the substrate capture site	23
^{1}H, ^{31}P & ^{13}C NMR Spectra of Compound **16**	24-26
^{1}H, ^{31}P & ^{13}C NMR Spectra of Compound **5**	27-29
^{1}H & ^{13}C NMR Spectra of Compound **19**	30-31
^{1}H, ^{31}P & ^{13}C NMR Spectra of Compound **20**	32-34
^{1}H, ^{31}P & ^{13}C NMR Spectra of Compound**7**	35-37
^{1}H, ^{31}P & ^{13}C NMR Spectra of Compound **21**	38-40
^{1}H, ^{31}P & ^{13}C NMR Spectra of Compound **22**	41-43
^{1}H, ^{31}P & ^{13}C NMR Spectra of Compound **23**	44-46
^{31}P NMR Spectrum of reaction progression in the formation of Compound **23a**	47
^{31}P NMR Spectrum of Compound **23a**	48
^{31}P NMR Spectrum of Compound **23b**	49
^{31}P NMR Spectrum of reaction mixture in the formation of Compound **23c**	50
^{31}P NMR Spectrum of reaction mixture in the formation of Compound **24**	51
^{1}H, ^{31}P & ^{13}C NMR Spectra of Compound **24**	52-54
^{1}H, ^{31}P & ^{13}C NMR Spectra of Compound **8**	55-57
^{1}H, ^{31}P & ^{13}C NMR Spectra of Compound **9**	58-60
^{1}H & ^{13}C NMR Spectra of Compound **25**	61-62
^{1}H & ^{13}C NMR Spectra of Compound **26**	63-64
^{1}H, ^{31}P & ^{13}C NMR Spectra of Compound **10**	65-67

Chemical Syntheses:

General Chemistry Methods

Chemistry: All reagents and solvents were of either commercial quality obtained from Sigma-Aldrich (Gillingham, Dorset, U.K.) or Acros-Fisher Scientific (Loughborough, U.K.) or GOSS Scientific Instruments Ltd (Great Baddow, Essex, U.K.) or synthesized and purified in the laboratory using standard procedures. Some solvents were redistilled and dried where necessary using standard procedures or purchased in anhydrous form. Petroleum ether (40-60 °C) is abbreviated as pet. ether. NMR spectra were recorded with a JOEL EX-270 or a Varian Mercury VX 400 or Bruker Avance III (400 MHz and 500 MHz) spectrometer. ^{1}H NMR and ^{13}C NMR chemical shifts are measured in ppm (δ) relative to internal tetramethylsilane (TMS) and ^{31}P NMR chemical shifts are measured in ppm (δ) relative to phosphoric acid as an external standard. Signals are expressed and abbreviated as, s (singlet), d (doublet), t (triplet), q (quartet), m (multiplet), br (broad) and ap (apparent). All ^{1}H NMR and ^{13}C NMR assingments are based on gCOSY, gHMQC, gHMBC and DEPT experiments. Coupling constants (J) are given in Hz. HRMS mass spectra were recorded at the University of Bath on a Bruker MicroTof 131 instrument or at the EPSRC National Mass Spectrometry Service Centre, University of Wales, Swansea. Microanalysis was carried out by the Microanalysis Service, University of Bath. Melting points (m.p.) were determined using a Reichert-Jung hot stage microscope apparatus or a Stanford Research Systems Optimelt MPA100 automated melting point system and are uncorrected. Thin-layer chromatography (TLC) was performed on precoated plates (Merck TLC aluminium sheets, silica gel 60 F_{254}) with detection by UV light or with phosphomolybdic acid in ethanol followed by heating. Flash chromatography was performed on silica gel (partical size 40-63 μm) using glass columns or on an ISCO CombiFlash Rf automated flash chromatography system using RediSep Rf disposable flash columns. Ion-exchange chromatography was performed on an LKB-Pharmacia medium-pressure ion exchange chromatograph with Q-Sepharose Fast Flow resin using a Pharmacia Biotech Gradifrac system with a P-1 pump; eluting with gradients of triethylammonium bicarbonate (TEAB) buffer and using H_2O of MilliQ quality. 2 M TEAB (pH 7.8) was prepared by bubbling carbon dioxide gas into 2 M triethylamine solution. Phosphate containing fractions were identified using a modification of the Briggs phosphate test (Lampe et al., 1994) and the target polyphosphates were accurately quantified using the Ames phosphate assay (Ames and Dubin, 1960).

2-*O*-Butanoyl 1,3,4,5,6-pentakis-*O*-[bis(benzyloxy)phosphoryl] *myo*-inositol (16)

To a solution of **14** (Godage et al., 2013) (323 mg, 1.29 mmol) and 5-phenyl-1*H*-tetrazole (1.89 g, 12.91 mmol) in dry dichloromethane (5 mL) under an atmosphere of argon, was added bis(benzyloxy)(*N*,*N*-diisopropylamino)phosphine (2.60 mL, 7.74 mmol). Stirring was continued for 1 h at room temperature, after which time TLC (1:1, ethyl acetate:pet. ether) confirmed the complete consumption of starting material (R_f 0.0) to product (R_f 0.9). The reaction mixture was cooled to −40 °C and 57% *m*CPBA (3.90 g, 12.91 mmol) was added portionwise while stirring. The cooling bath was removed and the mixture was allowed to reach room temperature. After 15 min, TLC (1:1, ethyl acetate:pet. ether) showed complete oxidation of pentakisphosphite to pentakisphosphate (R_f 0.2) and the reaction mixture was diluted with dichloromethane (150 mL), washed with 10% sodium sulphite solution (2 × 150 mL), dried and solvent evaporated *in vacuo*. The residue was purified by column chromatography (chloroform:acetone, 6:1) to afford **16** (1.83 g, 92%) as a colorless oil, ^{31}P NMR (161.9 MHz, H-decoupled, $CDCl_3$) δ −1.71 (2 P, s), −1.27 (2 P, s), −1.01 (1 P, s, phosphate at C-5); ^{1}H NMR (400 MHz, $CDCl_3$) δ 0.97 (3 H, t, *J* 7.4 Hz, $CO_2(CH_2)_2CH_3$), 1.67 (2 H, sextet, *J* 7.4 Hz, $CO_2CH_2CH_2CH_3$), 2.35 (2 H, t, *J* 7.4 Hz, $CO_2CH_2CH_2CH_3$), 4.49-4.58, 5.00-5.15 (3 H : 22 H, m, C-1-H, C-3-H, C-4-H, C-5-H, C-6-H and 10 × CH_2Ar), 6.15 (1 H, br s, C-2-H), 7.22-7.32 (50 H, m, Ar-H); ^{13}C NMR (100.6 MHz, $CDCl_3$) δ_C 13.4 ($CO_2(CH_2)_2CH_3$), 18.3 ($CO_2CH_2CH_2CH_3$), 35.7 ($CO_2CH_2CH_2CH_3$), 69.4 (C-2), 69.5, 69.5, 69.5, 69.6, 69.7, 69.7 (10 × CH_2Ar), 72.9, 74.7, 74.7 (C-1 & C-3, C-4 & C-6 and C-5), 127.8, 127.9, 128.0, 128.0, 128.0, 128.1, 128.1, 128.2, 128.2, 128.2, 128.3, 128.3, 128.4 (50 × Ar-C), 135.5, 135.6, 135.7, 135.8 (10 × Ar-C_{ipso}), 171.5 (s, $CO_2(CH_2)_2CH_3$); HRMS (ESI-TOF) *m/z*: $[M + H]^+$ Calcd for $C_{80}H_{84}O_{22}P_5$ 1551.4137; Found 1551.4106.

2-*O*-Butanoyl *myo*-inositol 1,3,4,5,6-pentakisphosphate (5)

Compound **16** (1.50 g, 0.97 mmol) was dissolved in methanol (30 mL) and water (5 mL) and 10% palladium hydroxide on activated charcoal (150 mg) were added. The resulting suspension was shaken in a Parr hydrogenator under H_2 for 18 h at room temperature. The catalyst was filtered through a PTFE syringe filter and the filtrate was evaporated under reduced pressure. The hygroscopic white foam was then purified by RP-18 silica gel column chromatography (0.05 M TEAB: Acetonitrile, 0 to 75%) to afford **5** (964 mg, 89%) as the triethylammonium salt, ^{31}P NMR (109.4 MHz, H-decoupled, D_2O) δ 0.17 (2 P, s), 1.24 (2 P, s), 1.59 (1 P, s, phosphate at C-5); ^{1}H NMR (400 MHz, D_2O) δ 0.76 (3 H, t, *J* 7.4 Hz, $CO_2(CH_2)_2CH_3$), 1.07 (~42 H, t, *J* 7.4

Hz, CH_3 of TEA^+), 1.47 (2 H, sextet, *J* 7.4 Hz, $CO_2CH_2CH_2CH_3$), 2.29 (2 H, t, *J* 7.4 Hz, $CO_2CH_2CH_2CH_3$), 2.99 (~29 H, q, *J* 7.4 Hz, CH_2 of TEA^+), 4.04-4.15 (3 H, m, *J* 2.3, 9.4, 9.8 Hz, C-1-H, C-3-H and C-5-H), 4.31 (2 H, ap. quartet, ddd, C-4-H and C-6-H), 5.53 (1 H, br s, C-2-H); ^{13}C NMR (100.6 MHz, D_2O) δ_C 8.1 (CH_3 of TEA^+), 12.9 ($CO_2(CH_2)_2CH_3$), 18.0 ($CO_2CH_2CH_2CH_3$), 35.8 ($CO_2CH_2CH_2CH_3$), 46.4 (CH_2 of TEA^+), 71.6 (C-2), 72.2 (C-1 and C-3), 76.3 (C-4 and C-6), 77.2 (C-5), 175.2 ($CO_2(CH_2)_2CH_3$); HRMS (ESI-TOF) *m/z*: $[M + Na]^+$ Calcd for $C_{10}H_{23}O_{22}P_5Na$ 672.9261; Found 672.9259.

2-*O*-Benzyl-1,6:3,4-bis-[*O*-(2,3-dimethoxybutane-2,3-diyl)]-*myo*-inositol (18)

Sodium hydride (60%, 235 mg, 5.88 mmol) was added portionwise to a suspension of 1,6:3,4-bis-[*O*-(2,3-dimethoxybutane-2,3-diyl)]-*myo*-inositol **17** (2.0 g, 4.90 mmol) in anhydrous DMF (120 mL). The resulting suspension was stirred for 1 h and benzyl bromide (0.64 mL, 5.39 mmol) was added dropwise over 30 min. Stirring was continued for a further 20 h, after which time TLC (hexane:ethyl acetate, 1:1) showed the complete conversion of starting material (R_f 0.0) to a product (R_f 0.4) and the excess sodium hydride was destroyed by the dropwise addition of methanol. The solvents were removed under reduced pressure and the residue was dissolved in dichloromethane (200 mL), washed with water (200 mL), brine (200 mL), dried ($MgSO_4$) and evaporated *in vacuo*. The resulting compound was purified by flash column chromatography (hexane:ethyl acetate, 2:1 to 1:1) to afford 2-*O*-benzyl ether **18** (1.92 g, 79%) as a white solid, m.p. 214–216 °C (ethanol), (Riley et al., 2012) m.p. 214-216 °C (ethanol); ^1H NMR (400 MHz, $CDCl_3$) δ 1.31 (12 H, s, 4 × CH_3), 2.40 (1 H, d, *J* 2.0 Hz, C-5-OH), 3.23 (6 H, s, 2 × OCH_3), 3.28 (6 H, s, 2 × OCH_3), 3.54 (2 H, dd, *J* 2.4, 10.1 Hz, C-1-H and C-3-H), 3.66 (1 H, dt, *J* 2.0, 9.6 Hz, C-5-H), 3.81 (1 H, t, *J* 2.4 Hz, C-2-H), 4.08 (2 H, dd, *J* 9.6, 10.1 Hz, C-4-H and C-6-H), 4.86 (2 H, s, OCH_2Ph), 7.22–7.26 (1 H, m, Ar-H), 7.29–7.34 (2 H, m, Ar-H), 7.50–7.53 (H, m, Ar-H); ^{13}C NMR ($CDCl_3$, 101 MHz) δ 17.7 (2 × CH_3), 17.8 (2 × CH_3), 47.9 (2 × OCH_3), 48.0 (2 × OCH_3), 69.1 (C-1 and C-3), 69.4 (C-4 and C-6), 70.6 (C-5), 73.8 ($PhCH_2O$), 76.2 (C-2), 99.1 (2 × $C(CH_3)OCH_3$), 99.6 (2 × $C(CH_3)OCH_3$), 126.9 (Ar-C_{para}), 127.6, 127.8 (Ar-C_{ortho} and Ar-C_{meta}), 139.6 (Ar-C_{ipso}); HRMS (ESI-TOF) *m/z*: $[M + Na]^+$ Calcd for $C_{25}H_{38}NaO_{10}$ 521.2357; Found 521.2337; Anal. Calcd for $C_{25}H_{38}O_{10}$ (498.57); C 60.23, H, 7.68; Found C 60.3, H, 7.44.

2-*O*-Benzyl-*myo*-inositol (19)

Aqueous TFA (90%, 5 mL) was added to a solution of 2-*O*-benzyl ether **18** (500 mg, 1.00 mmol) in DCM (5 mL). The reaction mixture was stirred for 1h at room temperature, after which time TLC (hexane:ethyl acetate, 1:1) indicated the complete conversion of starting material (R_f 0.4) to a product (R_f 0.0). The solvents were then removed by evaporation *in vacuo* followed by coevaporation with methanol a few times until all the traces of butanedione (yellow in colour) was removed to give the pure tetraol **19** (271 mg, quantitative) as a white solid, m.p. 248–250 °C (water), (Riley et al., 2006) m.p. 248–251 °C (water); ^{1}H NMR (400 MHz, DMF-d7) δ 3.38 (1 H, dt, *J* 4.2, 9.2 Hz, C-5-H), 3.69 (2 H, ddd, *J* 2.6, 5.1, 9.6 Hz, C-1-H and C-3-H), 3.88 (2 H, ddd, *J* 4.2, 9.2, 9.6 Hz, C-4-H and C-6-H), 4.13 (1 H, t, *J* 2.6 Hz, C-2-H), 4.94 (2 H, d, *J* 5.1 Hz, C-1-OH and C-3-OH), 4.98 (3 H, d, *J* 4.2 Hz, C-4-OH, C-5-OH and C-6-OH), 5.12 (2 H, s, OCH_2Ph), 7.47-7.51 (1 H, m, Ar-H_{para}), 7.55-7.59 (2 H, m, Ar-H_{meta}), 7.68-7.70 (2 H, m, Ar-H_{ortho}); ^{13}C NMR (101 MHz, DMF-d7) δ_C 73.2 (C-1 and C-3), 74.2 (C-4 and C-6), 74.9 (CH_2Ph), 76.3 (C-5), 82.4 (C-2), 127.1 (Ar-C_{para}), 127.4, 128.2 (Ar-C_{ortho} and Ar-C_{meta}), 140.5 (Ar-C_{ipso}); Anal. Calcd for $C_{13}H_{18}O_6$ (270.28), C 57.77; H 6.71; Found C 57.50, H 6.65.

2-*O*-Benzyl 1,3,4,5,6-pentakis-*O*-[bis(cyanoethyloxy)phosphoryl] *myo*-inositol (20)

To a solution of 2-*O*-benzyl *myo*-inositol **19** (200 mg, 0.74 mmol) and 1*H*-tetrazole (518 mg, 7.40 mmol) in dry dichloromethane (3 mL) under an atmosphere of argon, was added bis(cyanoethyl)(*N*,*N*-diisopropylamino)phosphine (1.48 g, 5.55 mmol). Stirring was continued for 2 h at room temperature, after which time TLC (ethyl acetate:ethanol, 4:1) confirmed the complete consumption of starting material (R_f 0.0) to a product (R_f 0.6) of which ^{31}P NMR (109 MHz, H-decoupled, $CDCl_3$) showed signals at δ 139.16 (1 P, m, phosphite at C-5), 140.49, 142.81 (2 × 2 P, 2 × m, phosphites at C-1 and C-3 & phosphites at C-4 and C-6). The reaction mixture was cooled to −40 °C and *m*CPBA (77%, 1.66 g, 7.40 mmol) was added portionwise while stirring. The cooling bath was removed and the mixture was allowed to reach room temperature. After 15 min, TLC (ethyl acetate:ethanol, 4:1) showed complete oxidation of pentakisphosphite to pentakisphosphate (R_f 0.2) and the reaction mixture was diluted with ethyl acetate (100 mL), washed with 10% sodium sulphite solution (2 × 200 mL), dried and solvent evaporated *in vacuo*. The resulting compound was purified by flash column chromatography (methanol in ethyl acetate 0 to 20%) to afford the protected pentakisphosphate

20 (690 mg, 78%) as a colorless oil, ^{31}P NMR (162 MHz, H-decoupled, $CDCl_3$) δ −3.64, −2.52 (2 × 2 P, 2 × s, phosphates at C-1 and C-3 & phosphates at C-4 and C-6), −2.44 (1 P, s, phosphate at C-5); ^{1}H NMR (400 MHz, $CDCl_3$) δ 2.73-2.92 (20 H, m, 10 × OCH_2CH_2CN), 4.29-4.54 (23 H, m, C-1-H, C-3-H, C-5-H and 10 × OCH_2CH_2CN), 4.70 (1 H, br s, C-2-H), 4.85 (2 H, ap. quartet, ddd, *J* 9.5, 9.6, 9.6 Hz, C-4-H and C-6-H), 4.92 (2 H, s, OCH_2Ph), 7.30–7.33 (1 H, m, Ar-H_{para}), 7.36-7.43 (4 H, m, Ar-H_{meta} and Ar-H_{ortho}); ^{13}C NMR (101 MHz, ACETONE-d6) δ 19.9, 20.0, 20.0, 20.0, 20.1, 20.1 (10 × OCH_2CH_2CN), 64.2, 64.3, 64.4, 64.4, 64.4, 64.4, 64.5 (10 × OCH_2CH_2CN), 75.9 (m, C-1 and C-3), 76.3 (m, C-5), 76.6 (m, C-4 and C-6), 77.2 (OCH_2Ph), 77.9 (C-2), 118.5, 118.5, 118.5, 118.5, 118.6 (10 × OCH_2CH_2CN), 128.6 (Ar-C_{para}), 128.7, 129.3 (Ar-C_{ortho} and Ar-C_{meta}), 139.2 (Ar-C_{ipso}); HRMS (ESI-TOF) *m/z*: $[M + Na]^+$ Calcd for $C_{43}H_{53}N_{10}NaO_{21}P_5$ 1223.1967; Found 1223.1951.

2-*O*-Benzyl-*myo*-inositol 1,3,4,5,6-pentakisphosphate (7)

Compound **20** (600 mg, 0.50 mmol) was then dissolved in concentrated aqueous ammonia solution (30 mL) and heated at 60 °C overnight in a Pyrex pressure tube. After evaporation of solution under vacuum, the residue was purified by ion exchange chromatography on Q Sepharose Fast Flow resin eluting with a gradient of aqueous TEAB (0 to 2.0 $moldm^{-3}$) to afford the pure triethylammonium salt of 2-*O*-benzyl *myo* inositol 1,3,4,5,6-pentakisphosphate **7** (549 mg, 95%) as a hygroscopic white solid, ^{31}P NMR (162 MHz, H-decoupled, D_2O) δ 0.32, 1.20 (2 × 2 P, 2 × s, phosphates at C-1 and C-3 and phosphates at C-4 and C-6), 1.70 (1 P, s, phosphate at C-5); ^{1}H NMR (400 MHz, D_2O) δ 1.05 (~47 H, t, *J* 7.2 Hz, CH_3 of TEA^+), 2.96 (~31 H, q, *J* 7.2 Hz, CH_2 of TEA^+), 3.95-4.05 (3 H, m, *J* 2.2, 9.4, 9.8 Hz, C-1-H, C-3-H and C-5-H), 4.25 (1 H, t, *J* 2.2 Hz, C-2-H), 4.30 (2 H, ap. quartet, ddd, *J* 9.4, 9.8 Hz, C-4-H and C-6-H), 4.74 (2 H, s, OCH_2Ph), 7.16-7.20 (1 H, m, Ar-H_{para}), 7.22-7.26 (2 H, m, Ar-H_{meta}), 7.37-7.39 (2 H, m, Ar-H_{ortho}); ^{13}C NMR (101 MHz, D_2O) δ_C 8.1 (CH_3 of TEA^+), 46.3 (CH_2 of TEA^+), 74.1 (m, C-1 and C-3), 75.8 (CH_2Ph), 76.2 (m, C-4 and C-6), 77.5 (m, C-5), 78.9 (C-2), 127.9 (Ar-C_{para}), 128.3 (Ar-C), 128.4 (Ar-C), 138.3 (Ar-C_{ipso}); HRMS (ESI-TOF) *m/z*: $[M - H]^-$ Calcd for $C_{13}H_{22}O_{21}P_5$ 668.9347; Found 668.9338.

2-*O*-Benzyl 5-*O*-[bis(cyanoethyloxy)phosphoryl] 1,6:3,4-bis-[*O*-(2,3-dimethoxybutane-2,3-diyl)]-*myo*-inositol (21)

To a solution of 2-*O*-benzyl 2,3-butanedione derivative **18** (950 mg, 1.91 mmol) and 5-phenyl-1*H*-tetrazole (418 mg, 2.86 mmol) in dry dichloromethane (10 mL) under an atmosphere of argon, was added bis(cyanoethyl)(*N*,*N*-diisopropylamino)phosphine (763 mg, 2.86 mmol). Stirring was continued for 1 h at room temperature, after which time ^{31}P NMR (161.9 MHz, H-decoupled, $CDCl_3$) showed a signal at δ 138.90 (1 P, s, phosphite at C-5) confirming the complete consumption of starting material. The reaction mixture was cooled to −40 °C and *m*CPBA (70%, 705 mg, 2.86 mmol) was added portionwise while stirring. The cooling bath was removed and the mixture was allowed to reach room temperature after which time ^{31}P NMR (161.9 MHz, H-decoupled, $CDCl_3$) showed a signal at δ −2.95 (1 P, s, phosphate at C-5) confirming the complete oxidation of product. The reaction mixture was diluted with dichloromethane (200 mL), washed with 10% sodium sulphite solution (2 × 200 mL), dried and solvent evaporated *in vacuo*. The resulting compound was purified by flash column chromatography (hexane:ethyl acetate, 1:4) to afford phosphate triester **21** (1.24 g, 95%) as a white foam, ^{31}P NMR (161.9 MHz, H-decoupled, $CDCl_3$) δ −2.82 (1 P, s, phosphate at C-5); ^{1}H NMR (400 MHz, $CDCl_3$) δ 1.27 (6 H, s, 2 × OCH_3), 1.30 (6 H, s, 2 × OCH_3), 2.77 (4 H, dt, *J* 3.1, 6.4 Hz, OCH_2CH_2CN), 3.21 (6 H, s, 2 × OCH_3), 3.27 (6 H, s, 2 × OCH_3), 3.55 (2 H, dd, *J* 2.4, 10.2 Hz, C-1-H and C-3-H), 3.80 (1 H, t, *J* 2.4 Hz, C-2-H), 4.18 (2 H, t, *J* 10.2 Hz, C-4-H and C-6-H), 4.27-4.42 (5 H, m, C-5-H and OCH_2CH_2CN), 4.84 (2 H, s, OCH_2Ph), 7.23–7.27 (1 H, m, Ar-H), 7.30–7.34 (2 H, m, Ar-H), 7.48–7.50 (2 H, m, Ar-H); ^{13}C NMR (100.6 MHz, $CDCl_3$) δ 17.5 (2 × CH_3), 17.6 (2 × CH_3), 19.5, 19.6 (2 × OCH_2CH_2CN), 48.0 (2 × OCH_3), 48.0 (2 × OCH_3), 62.0, 62.1 (2 × OCH_2CH_2CN), 67.8 (d, $^{3}J_{C,P}$ 3.5 Hz, C-4 and C-6), 68.6 (C-1 and C-3), 73.9 (OCH_2Ph), 75.7 (C-2), 77.9 (d, $^{2}J_{C,P}$ 6.5 Hz, C-5), 99.3 (2 × $C(CH_3)OCH_3$), 99.6 (2 × $C(CH_3)OCH_3$), 116.4 (2 × OCH_2CH_2CN), 127.1 (Ar-C_{para}), 127.7, 127.9 (Ar-C_{ortho} and Ar-C_{meta}), 139.2 (s, Ar-C_{ipso}); HRMS (ESI-TOF) *m/z*: $[M + Na]^+$ Calcd for $C_{31}H_{45}N_2NaO_{13}P$ 707.2551; Found 707.2585.

2-*O*-Benzyl 5-*O*-[bis(cyanoethyloxy)phosphoryl]-*myo*-inositol (22)

Aqueous TFA (90%, 2 mL) was added to a solution of phosphate triester **21** (250 mg, 0.37 mmol) in DCM (2 mL). The reaction mixture was stirred for 25 min at room temperature,

after which time TLC (ethyl acetate:methanol, 4:1) indicated the complete conversion of starting material (R_f 0.7) to a product (R_f 0.4). The solvents were then removed by evaporation *in vacuo* followed by coevaporation with methanol a few times until all the traces of butanedione (yellow colour) was removed to give the pure tetraol **22** (167 mg, quantitative) as a white solid, m.p. 156-158 °C (methanol); ^{31}P NMR (161.9 MHz, H-decoupled, $CDCl_3$) δ –2.88 (1 P, s, phosphate at C-5); ^{1}H NMR (400 MHz, $CDCl_3$) δ 2.96 (4 H, t, J 6.0 Hz, OCH_2CH_2CN), 3.55 (2 H, dd, J 2.4, 9.8 Hz, C-1-H and C-3-H), 3.90 (2 H, t, J 9.7 Hz, C-4-H and C-6-H), 3.99 (1 H, t, J 2.4 Hz, C-2-H), 4.16 (1 H, ap. quartet, ddd, J 9.3 Hz, C-5-H), 4.37-4.48 (4 H, m, OCH_2CH_2CN), 4.94 (2 H, s, OCH_2Ph), 7.29-7.33 (1 H, m, Ar-H), 7.36-7.40 (2 H, m, Ar-H), 7.48-7.50 (2 H, m, Ar-H); ^{13}C NMR (100.6 MHz, $CDCl_3$) δ 20.0, 20.0 (2 × OCH_2CH_2CN), 64.1, 64.2 (2 × OCH_2CH_2CN), 73.3 (d, $^3J_{C,P}$ 3.2 Hz, C-4 and C-6), 73.6 (C-1 and C-3), 76.4 (OCH_2Ph), 82.1 (C-2), 85.3 (d, $^2J_{C,P}$ 7.2 Hz, C-5), 118.6 (2 × OCH_2CH_2CN), 128.4 (Ar-C_{para}), 128.9, 129.2 (Ar-C_{ortho} and Ar-C_{meta}), 140.6 (Ar-C_{ipso}); HRMS (ESI-TOF) *m/z*: $[M + Na]^+$ Calcd for $C_{19}H_{25}N_2NaO_9P$ 479.1190; Found 479.1217.

2-*O*-Benzyl 5-*O*-[bis(cyanoethyloxy)phosphoryl] 1,3,4,6-pentakis-*O*-[bis(benzyloxy)phosphoryl] *myo*-inositol (23)

To a solution of tetraol **22** (167 mg, 0.37 mmol) and 5-phenyl-1*H*-tetrazole (320 mg, 2.19 mmol) in dry dichloromethane (5 mL) under an atmosphere of argon, was added bis(benzyloxy)(*N*,*N*-diisopropylamino)phosphine (0.74 mL, 2.19 mmol). Stirring was continued for 1.5 h at room temperature, after which time TLC (ethyl acetate:methanol, 9:1) confirmed the complete consumption of starting material (R_f 0.2) to a product (R_f 0.8) of which ^{31}P NMR (161.9 MHz, H-decoupled, $CDCl_3$) showed signals at δ –2.88 (1 P, s, phosphate at C-5), 140.93 (2 P, d, J 4.9 Hz), 143.52 (2 P, d, J 4.9 Hz). The reaction mixture was cooled to –40 °C and *m*CPBA (70%, 540 mg, 2.19 mmol) was added portionwise while stirring. The cooling bath was removed and the mixture was allowed to reach room temperature. After 30 min, TLC (ethyl acetate:methanol, 9:1) showed complete oxidation of tetrakisphosphite to pentakisphosphate (R_f 0.6) of which ^{31}P NMR (161.9 MHz, H-decoupled, $CDCl_3$) showed signals at δ –2.95 (1 P, s, phosphate at C-5), –2.10 (2 P, s), –1.75 (2 P, s). The reaction mixture was diluted with ethyl acetate (100 mL), washed with 10% sodium sulphite solution (2 × 100 mL), dried and solvent evaporated *in vacuo*. The resulting compound was purified by flash column chromatography (ethyl acetate in pet. ether, 60 to 100%) to afford 2-

O-benzyl pentakisphosphate **23** (514 mg, 94%) as a colorless oil, ^{31}P NMR (161.9 MHz, H-decoupled, $CDCl_3$) δ −2.87 (1 P, s, phosphate at C-5), −1.91 and −1.62 (2 × 2 P, 2 × s, phosphates at C-1 and C-3 & phosphates at C-4 and C-6); ^{1}H NMR (400 MHz, $CDCl_3$) δ 2.48 (4 H, t, *J* 6.3 Hz, OCH_2CH_2CN), 4.09-4.20 (4H, m, OCH_2CH_2CN), 4.26 (2 H, ddd, *J* 2.3, 10.0 Hz, C-1-H and C-3-H), 4.38 (1 H, ap. quartet, ddd, *J* 9.5, 9.8 Hz, C-5-H), 4.75 (3 H, br s, C-2-H and OCH_2Ph), 4.87-5.08 (18 H, m, 8 × OCH_2Ph, C-4-H and C-6-H), 7.19–7.28 (45 H, m, Ar-H); ^{13}C NMR (100.6 MHz, $CDCl_3$) δ 19.0, 19.1 (2 × OCH_2CH_2CN), 62.6, 62.7 (2 × OCH_2CH_2CN), 69.6, 69.7, 69.7, 69.8, 69.9 (8 × OCH_2Ph), 74.9 (m, C-1 and C-3), 75.1 (m, C-4 and C-6), 76.1 (OCH_2Ph), 76.3 (m, C-5), 76.7 (C-2), 116.8 (2 × OCH_2CH_2CN), 127.4, 127.6, 128.0, 128.1, 128.1, 128.2, 128.3, 128.5, 128.5, 128.6, 128.6 (Ar-C_{para}, Ar-C_{ortho} and Ar-C_{meta}), 135.3, 135.4, 135.4, 135.5, 135.6, 135.7, 135.8, 137.8 (Ar-C_{ipso}), HRMS (ESI-TOF) *m/z*: $[M + Na]^+$ Calcd for $C_{75}H_{77}N_2NaO_{21}P_5$ 1519.3599; Found 1519.3588.

2-*O*-Benzyl 5-diphosphoinositol 1,3,4,6-tetrakisphosphate (8)

To a solution of 2-*O*-Bn pentakisphosphate **23** (42 mg, 0.03 mmol) in dry $CDCl_3$ (1 mL) under an atmosphere of argon, was added DBU (16.76 µL, 0.11 mmol) followed by BSTFA (29.77 µL, 0.11 mmol) and the reaction mixture was monitored by ^{31}P NMR. After stirring the reaction mixture for 1 h at room temperature, ^{31}P NMR (161.9 MHz, H-decoupled, $CDCl_3$) showed signals at δ −17.52 (1 P, s, phosphate at C-5), −2.28, −1.68 (2 × 2 P, 2 × s, phosphates at C-1 and C-3 & phosphates at C-4 and C-6) confirming the complete removal of cyanoethyl protection on C-5 phosphate of starting material to the TMS protected phosphate at the C-5 position (**23a**, see the attached spectra). MeOH (28.38 µL, 0.70 mmol) followed by TFA (8.59 µL, 0.11 mmol) was then added to the reaction mixture and stirred for 15 min after which time ^{31}P NMR (161.9 MHz, H-decoupled, $CDCl_3$) showed signals at δ −3.04, −2.25 (2 × 2 P, 2 × s, phosphates at C-1 and C-3 & phosphates at C-4 and C-6) and −0.51 (1 P, s, phosphate at C-5) confirming the complete removal of TMS protection on C-5 phosphate to provide the phosphate monoester at the C-5 position (**23b**, see the attached spectrum). The solvents were then evaporated and the residue was dried under vacuum. The residue was redissolved in $CDCl_3$ (2 mL) under an atmosphere of argon, was added 5-phenyl-1*H*-tetrazole (6.15 mg, 0.04 mmol), followed by bis(benzyloxy)(*N*,*N*-diisopropylamino)phosphine (14.14 µL, 0.04 mmol). Stirring was continued for 30 min at room temperature, after which time ^{31}P NMR (161.9 MHz, H-decoupled, $CDCl_3$) showed signals at δ −10.94 (1 P, s, C-5-O-

P(O)(OH)OP(OBn)$_2$), −2.28, −2.11 (2 × 2 P, 2 × s, phosphates at C-1 and C-3 & phosphates at C-4 and C-6), 127.82 (1 P, s, C-5-O-P(O)(OH)O*P*(OBn)$_2$) confirming the completion of the phosphitylation of C-5 phosphate (**23c**, see the attached spectrum). The reaction mixture was cooled to −40 °C and 70% *m*CPBA (10.37 mg, 0.04 mmol) was added portionwise while stirring. The cooling bath was removed and the mixture was allowed to reach room temperature. After 15 min, ^{31}P NMR (161.9 MHz, H-decoupled, $CDCl_3$) showed signals at δ 13.44 (1 P, d, *J* 14.6 Hz, C-5-O-*P*(O)(OH)OP(O)(OBn)$_2$), 11.23 (1 P, d, *J* 14.6 Hz, C-5-O-P(O)(OH)O*P*(O)(OBn)$_2$), −2.28, −1.99 (2 × 2 P, 2 × s, phosphates at C-1 and C-3 & phosphates at C-4 and C-6) confirming the completion of the oxidation (see the attached spectrum of the reaction mixture). The reaction mixture was diluted with dichloromethane (100 mL), washed with 10% sodium sulphite solution (2 × 100 mL), dried ($MgSO_4$) and solvent evaporated *in vacuo*. Without further purification, the crude product was redissolved in *t*-BuOH (2 mL) and deionised water (0.5 mL); $NaHCO_3$ (25.92 mg, 0.31 mmol) and 10% Pd(OH)$_2$ on activated charcoal (15 mg) were then added and stirred under an atmosphere of H_2 (using a balloon) at room temperature. After 1 h, more deionised water (1.5 mL) was added and the hydrogenolysis continued for a further 30 min. The catalyst was removed by filtration through a PTFE syringe filter and the filtrate was concentrated under reduced pressure. The residue was purified by ion-exchange chromatography on Q Sepharose Fast Flow resin eluting with a gradient of aqueous TEAB (0 to 2.0 moldm^{-3}) to give the triethylammonium salt of 2-O-Bn-5PP-IP$_4$ **8** (21 mg, 58%) as a white foam, ^{31}P NMR (161.9 MHz, H-decoupled, D_2O) δ −11.24 (1 P, d, *J* 21.1 Hz, C-5-OP), −10.76 (1 P, d, *J* 21.1 Hz, POPO_3^{2-}), 0.20, 0.55 (2 × 2 P, 2 × s, C-1-P and C-3-P & C-4-P and C-6-P); ^{1}H NMR (500 MHz, D_2O) δ 1.16 (~48 H, t, *J* 7.3 Hz, CH_3 of TEA$^+$), 3.08 (~31 H, q, *J* 7.3 Hz, CH_2 of TEA$^+$), 4.16 (2 H, ddd, *J* 2.6, 9.9 Hz, C-1-H and C-3-H), 4.20 (1 H, ap. quartet, ddd, *J* 8.6 Hz, C-5-H), 4.25 (1 H, t, *J* 2.6 Hz, C-2-H), 4.45 (2 H, ap. quartet, ddd, *J* 9.4 Hz, C-4-H and C-6-H), 4.84 (2 H, s, OC*H*$_2$Ph), 7.26-7.29 (1 H, m, Ar-H$_{para}$), 7.32-7.35 (2 H, m, Ar-H$_{meta}$), 7.48-7.49 (2 H, m, Ar-H$_{ortho}$); ^{13}C NMR (125.8 MHz, D_2O) δ_C 8.2 (CH_3 of TEA$^+$), 46.6 (CH_2 of TEA$^+$), 74.3 (d, *J* 5.4 Hz, C-1 and C-3), 76.1 (*C*H$_2$Ph), 76.3 (m, C-4 and C-6), 77.8 (broad, C-5), 79.1 (C-2), 128.0 (Ar-C$_{para}$), 128.5, 128.6 (Ar-C$_{ortho}$ and Ar-C$_{meta}$), 138.2 (Ar-C$_{ipso}$); HRMS (ESI-TOF) *m/z*: [M − H]$^-$ Calcd for $C_{13}H_{23}O_{24}P_6$ 748.9010; Found 748.9020.

2-*O*-Benzyl 5-*O*-[[Bis(benzyloxy)phosphoryloxy]benzyloxyphosphoryl]1,3,4,6-tetrakis-*O*-[bis(benzyloxy)phosphoryl] *myo*-inositol (24)

To a solution of pentakisphosphate **23** (150 mg, 0.10 mmol) in dry $CDCl_3$ (2 mL) under an atmosphere of argon, was added DBU (59.87 µL, 0.40 mmol) followed by BSTFA (106.33 µL, 0.40 mmol) and the reaction mixture was monitored by ^{31}P NMR. After stirring the reaction mixture for 1 h at room temperature, ^{31}P NMR (161.9 MHz, H-decoupled, $CDCl_3$) showed signals at δ –17.52 (1 P, s, phosphate at C-5), – 2.28, – 1.68 (2 × 2 P, 2 × s, phosphates at C-1 and C-3 & phosphates at C-4 and C-6) confirming the complete removal of cyanoethyl protection on C-5 phosphate of starting material to the TMS protected phosphate at the C-5 position (**23a**, see the attached spectra). MeOH (101.34 µL, 2.50 mmol) followed by TFA (30.66 µL, 0.40 mmol) was then added to the reaction mixture and stirred for 15 min after which time ^{31}P NMR (161.9 MHz, H-decoupled, $CDCl_3$) showed signals at δ –3.04, –2.25 (2 × 2 P, 2 × s, phosphates at C-1 and C-3 & phosphates at C-4 and C-6) and –0.51 (1 P, s, phosphate at C-5) confirming the complete removal of TMS protection on C-5 phosphate to provide the phosphate monoester (**23b**, see the attached spectrum). The solvents were then evaporated and the residue was dried under vacuum. The residue was redissolved in $CDCl_3$ (3 mL) under an atmosphere of argon, was added 5-phenyl-1*H*-tetrazole (21.96 mg, 0.15 mmol), followed by bis(benzyloxy)(*N*,*N*-diisopropylamino)phosphine (50.49 µL, 0.15 mmol). Stirring was continued for 30 min at room temperature, after which time ^{31}P NMR (161.9 MHz, H-decoupled, $CDCl_3$) showed signals at δ –10.94 (1 P, s, C-5-O-*P*(O)(OH)OP(OBn)$_2$), –2.28, –2.11 (2 × 2 P, 2 × s, phosphates at C-1 and C-3 & phosphates at C-4 and C-6), 127.82 (1 P, s, C-5-O-P(O)(OH)O*P*(OBn)$_2$) confirming the completion of the phosphitylation of C-5 phosphate (**23c**, see the attached spectrum). The reaction mixture was cooled to –40 °C and 70% *m*CPBA (37.05 mg, 0.15 mmol) was added portionwise while stirring. The cooling bath was removed and the mixture was allowed to reach room temperature. After 30 min, ^{31}P NMR (161.9 MHz, H-decoupled, $CDCl_3$) showed signals at δ 13.44 (1 P, d, *J* 14.6 Hz, C-5-O-*P*(O)(OH)OP(O)(OBn)$_2$), 11.23 (1 P, d, *J* 14.6 Hz, C-5-O-P(O)(OH)O*P*(O)(OBn)$_2$), –2.28, –1.99 (2 × 2 P, 2 × s, phosphates at C-1 and C-3 & phosphates at C-4 and C-6) confirming the completion of the oxidation (see the attached spectrum of the reaction mixture). The reaction mixture was diluted with dichloromethane (200 mL), washed with 10% sodium sulphite solution (2 × 200 mL), dried ($MgSO_4$) and solvent evaporated *in vacuo*. The resulting compound was purified by flash column chromatography (ethyl acetate in pet.ether, 0 to 100% followed by MeOH in ethyl acetate, 0 to 20%) to obtain hexakisphosphate **24** (130 mg, 79%) as a colorless

oil, ^{31}P NMR (161.9 MHz, H-decoupled, $CDCl_3$) δ −12.77 (1 P, broad), −10.74 (1 P, d, *J* 14.6 Hz), −2.87 and −2.31 (2 × 2 P, 2 × s, phosphates at C-1 and C-3 & phosphates at C-4 and C-6); ^{1}H NMR (400 MHz, $CDCl_3$) δ 3.86-5.14 (28 H, br peaks, m, C-1-H, C-2-H, C-3-H, C-4-H, C-5-H, C-6-H and 22 × OCH_2Ph), 7.04-7.25 (55H, m, Ar-H); ^{13}C NMR (100.6 MHz, $CDCl_3$) δ 69.5, 69.5, 69.7, 69.7, 70.0, 70.2 (br peaks, O*C*H_2Ph), 75.1 (br m, Inositol-*C*), 76.7(Inositol-*C*), 127.2, 127.3, 127.8, 128.0, 128.1, 128.1, 128.2, 128.2, 128.3, 128.4, 128.5, 128.5 (Ar-C_{para}, Ar-C_{ortho} and Ar-C_{meta}), 135.5, 135.5, 135.6, 135.7, 135.9, 138.0 (Ar-C_{ipso}); HRMS (ESI-TOF) *m/z*: $[M + Na]^+$ Calcd for $C_{83}H_{84}NaO_{24}P_6$ 1673.3670; Found 1673.3623.

5-diphosphoinositol 1,3,4,6-tetrakisphosphate (9)

Compound **24** (130 mg, 0.08 mmol) was dissolved in *t*-BuOH (2 mL) and deionised water (0.5 mL). $NaHCO_3$ (72.74 mg, 0.87 mmol) and 10% $Pd(OH)_2$ on activated charcoal (46 mg) were then added and stirred under an atmosphere of H_2 (using a balloon) at room temperature. After 1 h, more deionised water (1.5 mL) was added and the hydrogenolysis continued for a further 30 min. The catalyst was removed by filtration through a PTFE syringe filter and the filtrate was concentrated under reduced pressure. The residue was purified by ion-exchange chromatography on Q Sepharose Fast Flow resin eluting with a gradient of aqueous TEAB (0 to 2.0 moldm^{-3}) to give the triethylammonium salt of 5-diphosphoinositol tetrakisphosphate **9** (61 mg, 65%) as a white foam; ^{31}P NMR (161.9 MHz, H-decoupled, D_2O) δ −11.38 (1 P, d, *J* 21.1 Hz, C-5-OP), −10.80 (1 P, d, *J* 21.1 Hz, POPO_3^{2-}), 0.31, 0.60 (2 × 2 P, 2 × s, C-1-P and C-3-P & C-4-P and C-6-P); ^{1}H NMR (400 MHz, D_2O) δ 1.17 (~48 H, t, *J* 7.3 Hz, CH_3 of TEA^+), 3.10 (~31 H, q, *J* 7.3 Hz, CH_2 of TEA^+), 4.06 (2 H, ddd, *J* 2.7, 9.9 Hz, C-1-H and C-3-H), 4.17 (1 H, ap. quartet, ddd, *J* 9.0 Hz, C-5-H), 4.20 (1H, t, *J* 2.6 Hz, C-2-H), 4.17 (2H, ap. quartet, ddd, *J* 9.4 Hz, C-4-H and C-6-H); ^{13}C NMR (100.6 MHz, D_2O) δ_C 8.2 (CH_3 of TEA^+), 46.5 (CH_2 of TEA^+), 70.7 (C-2), 74.1 (d, *J* 5.1 Hz, C-1 and C-3), 75.9 (m, C-4 and C-6), 77.7 (m, C-5); HRMS (ESI-TOF) *m/z*: $[M - H]^-$ Calcd for $C_6H_{17}O_{24}P_6$ 658.8541; Found 658.8555.

2,5-Di-*O*-benzyl-1,6:3,4-bis-[*O*-(2,3-dimethoxybutane-2,3-diyl)]-*myo*-inositol (25)

Sodium hydride (60%, 343 mg, 8.57 mmol) was added portionwise to a suspension of diol **17** (1.0 g, 2.45 mmol) in anhydrous DMF (40 mL). The resulting suspension was stirred for 30 min and benzyl bromide (0.82 mL, 6.86 mmol) was added dropwise over 5 min. Stirring was continued for a further 18 h, after which time TLC (hexane:ethyl acetate, 1:1) showed the

complete conversion of starting material (R_f 0.0) to a product (R_f 0.7) and the excess sodium hydride was destroyed by the dropwise addition of methanol. The solvents were removed under reduced pressure and the residue was dissolved in dichloromethane (100 mL), washed with water (100 mL), brine (100 mL), dried ($MgSO_4$) and evaporated *in vacuo*. The resulting compound was purified by flash column chromatography (hexane:ethyl acetate, 2:1 to 1:1) to afford 2,5-di-*O*-benzyl ether **25** (1.41 g, 98%) as a white solid, m.p. 164-166 °C (ethanol); ^{1}H NMR (400 MHz, $CDCl_3$) δ 1.26 (6 H, s, 2 × CH_3), 1.28 (6 H, s, 2 × CH_3), 3.19 (6 H, s, 2 × OCH_3), 3.21 (6 H, s, 2 × OCH_3), 3.47-3.54 (3 H, m, C-1-H, C-3-H and C-5-H), 3.75 (1 H, br s, C-2-H), 4.14 (2 H, t, *J* 9.4, 10.2 Hz, C-4-H and C-6-H), 4.80 (2 H, d, *J* 2.0 Hz, OCH_2Ph), 4.81 (2 H, d, *J* 2.0 Hz, OCH_2Ph), 7.16-7.20 (2 H, m, Ar-H), 7.22-7.27 (4 H, m, Ar-H), 7.34-7.37 (2 H, m, Ar-H), 7.44-7.46 (2 H, m, Ar-H); ^{13}C NMR (101 MHz, $CDCl_3$) δ_C 17.6 (2 × CH_3), 17.9 (2 × CH_3), 47.8 (2 × OCH_3), 47.9 (2 × OCH_3), 69.3 (C-1 and C-3), 69.9 (C-4 and C-6), 73.7 (OCH$_2$Ph), 75.0 (OCH$_2$Ph), 76.0 (C-2), 78.8 (C-5), 99.0 (2 × C(CH_3)OCH_3), 99.5 (2 × C(CH_3)OCH_3), 126.9, 127.2 (2 × Ar-C_{para}), 127.5, 127.7, 127.8, 128.0 (2 × Ar-C_{ortho} and Ar-C_{meta}), 139.6 (2 × Ar-C_{ipso}); Anal. Calcd for $C_{32}H_{44}O_{10}$; C 65.29, H, 7.53; found C 65.20, H, 7.63.

2,5-Di-*O*-benzyl-*myo*-inositol (26)

A mixture of TFA (1.8 mL) and water (0.2 mL) was added to a solution of 2,5-di-*O*-benzyl ether **25** (300 mg, 0.51 mmol) in DCM (2 mL). The reaction mixture was stirred for 30 min at room temperature, after which time TLC (hexane:ethyl acetate, 1:1) indicated the complete conversion of starting material (R_f 0.7) to a product (R_f 0.0). The solvents were then removed by evaporation *in vacuo* followed by coevaporation with methanol a few times until all the traces of butanedione (yellow in colour) was removed to give the pure tetraol **26** (184 mg, quantitative) as a white solid, m.p. 267-269 °C (methanol), (Mills and Potter, 2003) m.p. 271-273 °C (DMF-ethanol); ^{1}H NMR (400 MHz, DMSO-d6) δ 3.09 (1 H, t, *J* 9.1 Hz, C-5-H), 3.38 (2 H, dd, *J* 2.2, 9.7 Hz, C-1-H and C-3-H), 3.65 (2 H, t, *J* 9.4 Hz, C-4-H and C-6-H), 3.79 (1 H, br s, C-2-H), 4.84 (8 H, br s, C-1-OH, C-3-OH, C-4-OH, C-6-OH and 2 × OCH_2Ph), 7.27-7.48 (10 H, m, Ar-H); ^{13}C NMR (101 MHz, DMSO-d6) δ_C 72.1 (C-1 and C-3), 73.0 (C-4 and C-6), 73.7, 74.1 (2 × CH$_2$Ph), 81.8 (C-2), 84.2 (C-5), 126.9, 126.9 (2 × Ar-C_{para}), 127.0, 127.5, 127.9, 127.9 (2 × Ar-C_{ortho} and Ar-C_{meta}), 139.9, 139.9 (2 × Ar-C_{ipso}); HRMS (ESI-TOF) *m/z*: $[M + Na]^+$ Calcd for $C_{20}H_{24}NaO_6$ 383.1465; Found 383.1466.

***Myo* inositol 2,5-di-*O*-benzyl 1,3,4,6-tetrakisphosphate (10)**

To a solution of 2,5-di-*O*-benzyl *myo*-inositol **26** (160 mg, 0.44 mmol) and 5-phenyl-1*H*-tetrazole (389 mg, 2.66 mmol) in dry dichloromethane (5 mL) under an atmosphere of argon, was added bis(cyanoethyl)(*N*,*N*-diisopropylamino)phosphine (711 mg, 2.66 mmol). Stirring was continued for 1 h at room temperature, after which time TLC (ethyl acetate:methanol, 9:1) confirmed the complete consumption of starting material (R_f 0.2) to a product (R_f 0.6) of which ^{31}P NMR (109.4 MHz, H-decoupled, $CDCl_3$) showed signals at δ 140.40 (2 P, s) and 142.51 (2 P, s). The reaction mixture was cooled to −40 °C and *t*-BuOOH (0.38 mL, 2.66 mmol) was added portionwise while stirring. The cooling bath was removed and the mixture was allowed to reach room temperature. After 30 min, TLC (ethyl acetate:methanol, 9:1) showed complete oxidation of tetrakisphosphite to tetrakisphosphate (R_f 0.3) of which ^{31}P NMR (109.4 MHz, H-decoupled, $CDCl_3$) showed signals at δ −2.81 (2 P, s) and −2.08 (2 P, s). The reaction mixture was diluted with ethyl acetate (100 mL), washed with 10% sodium sulphite solution (2 × 100 mL), dried and solvent evaporated *in vacuo* to afford the crude 2,5-di-*O*-benzyl 1,3,4,6-tetrakis-*O*-[bis(cyanoethyloxy)phosphoryl] *myo* inositol. Without further purification crude product was then dissolved in concentrated aqueous ammonia solution (30 mL) and heated at 70 °C overnight in a Pyrex pressure tube. After evaporation of solution under vacuum, the residue was purified by ion exchange chromatography on Q Sepharose Fast Flow resin eluting with a gradient of aqueous TEAB (0 to 2.0 moldm^{-3}) to afford the pure triethylammonium salt of 2,5-di-*O*-benzyl tetrakisphosphate **10** (294 mg, 64%) as a hygroscopic white solid, ^{31}P NMR (161.9 MHz, H-decoupled, D_2O) δ −0.77 and 0.09 (2 × 2 P, 2 × s, phosphates at C-1 and C-3 & phosphates at C-4 and C-6); ^{1}H NMR (400 MHz, D_2O) δ 1.12 (~32 H, t, *J* 7.4 Hz, CH_3 of TEA^+), 3.03 (~22 H, q, *J* 7.4 Hz, CH_2 of TEA^+), 3.59 (1 H, t, *J* 9.4 Hz, C-5-H), 4.16 (1 H, ddd, *J* 2.3, 9.8 Hz, C-1-H and C-3-H), 4.24 (1 H, t, *J* 2.3 Hz, C-2-H), 4.44 (2 H, ap. quartet, ddd, *J* 9.4, 9.8 Hz, C-4-H and C-6-H), 4.76 (2 H, s, OCH_2Ph), 4.81 (2 H, s, OCH_2Ph), 7.23-7.33 (6 H, m, Ar-H), 7.44-7.50 (4 H, m, Ar-H); ^{13}C NMR (100.6 MHz, D_2O) δ_C 8.1 (CH_3 of TEA^+), 46.5 (CH_2 of TEA^+), 74.6 (CH_2Ph), 74.7 (m, C-1 and C-3), 76.0 (CH_2Ph), 77.0 (m, C-4 and C-6), 79.1 (C-2), 79.8 (C-5), 128.0, 128.1 (2 × Ar-C_{para}), 128.3, 128.4, 128.5, 129.3 (2 × Ar-C_{ortho} and Ar-C_{meta}), 137.4, 138.1 (2 × Ar-C_{ipso}); HRMS (ESI-TOF) *m/z*: $[M + Na]^+$ Calcd for $C_{20}H_{28}NaO_{18}P_4$ 703.0118; Found 703.0103.

Protein Expression, Purification, Crystallization and Structure Determination

Full-length human IP6K2, the kinase domain of human PPIP5K2 (PPIP5K2^{KD}; residues 1-366), and the N-terminally truncated domain used for the crystallography studies (residues 41-366) were all sub-cloned, expressed and purified as before (Wang et al., 2012; Weaver et al., 2013). The PPIP5K2^{KD} was crystallized by hanging drop vapor diffusion against a well buffer of 12% (w/v) PEG 3350, 20 mM $MgCl_2$, 0.1 M HEPES, pH 7.0, 1 mM AMP-PNP and 2 mM $CdCl_2$ at 4 °C. The crystals were then soaked with 5-10 mM compounds in a stabilizing buffer containing 22% (w/v) PEG 3350, 10 mM $MgCl_2$, 0.1 M sodium acetate, pH 5.2 or 7.0 at 4 °C for 3 days. Cryosolvent was prepared by adding 33% ethylene glycol into the soaking buffer. Diffraction data were collected using APS beamlines 22-BM and 22-ID. All data were processed with the program HKL2000. The structure was determined using rigid body and direct Fourier synthesis, and refined with the equivalent and expanded test sets. The structure was further manually rebuilt with COOT and refined with PHENIX and REFMAC from the CCP4 package. Ligand topology and parameter files were prepared using the PRODRG server. The molecular graphics representations were prepared with the program PyMol (Schrödinger, LLC). The 2D ligand-protein interaction diagrams were generated by LigPlot+. Atomic coordinates and structure factors have been deposited with the Protein Data Bank with accession codes: 4NZM, 4NZN, 4NZO.

Mutagenesis

Mutants were generated using a site-directed mutagenesis kit (Stratagene). Pairs of complementary primers were designed as shown below, with mutagenic primers underlined (only 5' primers are given):

K54A: GGAATATGTTCCATGGCAAAG**<u>GCA</u>**TCCAAATCCAAACCAATGAAG

K103A:
CCTTTATGTGATTGTCTTATTTCTTTCCATTCT**<u>GCA</u>**GGATTTCCACTGGACAAAGCGG

E192G:
TTTGTAGAAAAGCCAGTCAGTGCA**<u>GGA</u>**GATCACAATGTTTACATTTATTACCCAACTTC

E192Q:
TTTGTAGAAAAGCCAGTCAGTGCA**<u>CAA</u>**GATCACAATGTTTACATTTATTACCCAACTTC

All mutated constructs were sequenced. Mutant proteins were expressed and purified as for wild-type protein.

References

Ames, B.N., and Dubin, D.T. (1960). The role of polyamines in the neutralization of bacteriophage deoxyribonucleic acid. J. Biol. Chem. *235*, 769-775.

Godage, H.Y., Riley, A.M., Woodman, T.J., Thomas, M.P., Mahon, M.F., and Potter, B.V.L. (2013). Regioselective opening of *myo*-inositol orthoesters: mechanism and synthetic utility. J. Org. Chem. *78*, 2275-2288.

Lampe, D., Liu, C.S., and Potter, B.V.L. (1994). Synthesis of selective non-Ca^{2+}-mobilizing inhibitors of D-*myo*-inositol 1,4,5-trisphosphate 5-phosphatase. J. Med. Chem. *37*, 907-912.

Mills, S.J., and Potter, B.V.L. (2003). Synthesis of potent Ins(1,4,5)P_3 5-phosphatase inhibitors by modification of *myo*-inositol 1,3,4,6-tetrakisphosphate. Bioorg. Med. Chem. *11*, 19, 4245-4253.

Riley, A.M., Trusselle, M., Kuad, P., Borkovec, M., Cho, J., Choi, J.H., Qian, X., Shears, S.B., Spiess, B., and Potter, B.V.L. (2006). *Scyllo*-inositol pentakis-phosphate as an analog of *myo*-inositol 1,3,4,5,6-pentakis-phosphate: chemical synthesis, physico-chemistry and biological applications. ChemBioChem. *7*, 1114-1122.

Riley, A. M., Wang, H., Weaver, J. D., Shears, S. B., and Potter, B. V. L. (2012). First synthetic analogues of diphosphoinositol polyphosphates: interaction with PPIP5 kinase. Chem. Commun. *48*, 11292-11294.

Wang, H., Falck, J.R., Hall, T.M., and Shears, S.B. (2012). Structural basis for an inositol pyrophosphate kinase surmounting phosphate crowding. Nat. Chem. Biol. *8*, 111-116.

Weaver, J. D., Wang, H. and Shears, S. B. (2013). The kinetic properties of a human PPIP5K reveal that its kinase activities are protected against the consequences of a deteriorating cellular bioenergetic environment. Biosci. Rep. *33*, 228-241.

Table.S1, related to Figure 5. Data collection and structure refinement statistics

**PDB Accession Codes**	4NZM	4NZN	4NZO
**Data collection**	10 mM 5-PA-InsP5 at pH 5.2	5 mM 2-*O*-Bn-5-PA-InsP$_4$ at pH 5.2	10 mM 2,5-di-*O*-Bn-InsP$_4$ at pH7.0
Cell dimensions (a,b, c (Å))	111.2 41.4 89.6	110.7 41.3 89.3	111.1, 41.3, 89.1
Resolution (Å)*	50-2.0 (2.03)	50-1.75 (1.79)	50-1.9 (1.93)
Rsym *	0.114 (0.511)	0.071 (0.531)	0.073 (0.460)
I/σI*	17.4 (3.2)	28.0 (2.5)	29.3 (3.7)
Completeness (%)*	97.4 (91.3)	99.8 (97.9)	99.9 (99.6)
Redundancy *	4.9 (3.0)	5.7 (4.8)	7.2 (6.4)
**Refinement**			
Resolution(Å)*	34.28-2.0 (2.05)	33.2-1.75 (2.05)	35.53-1.9 (1.95)
No. reflections	26130	39720	31228
R_{work} *	19.8 (20.8)	14.1(22.7)	17.9 (22.2)
R_{free} *	23.1 (24.5)	18.0(27.6)	21.5 (26.8)
**No. atoms**			
Protein	2700	2639	2658
Analog	78	42	42
AMP-PNP	31	31	31
Mg	4	3	3
Solvent	348	348	342
**B-factors (Å2)**			
Protein	22.5	22.5	26.2
Analog	28.7	62.4	65.5
AMP-PNP	20.7	15.6	22.0
Mg	35.7	17.5	23.7
Solvent	35.6	37.9	36.8
**R.m.s. deviations**			
Bond length(Å)	0.01	0.006	0.01
Bond Angle (˚)	1.30	1.38	1.54

* The numbers in parentheses are for the highest resolution shell

Figure Legends

S1, related to Figure 4. 2,5-Di-*O*-Bn-InsP$_4$ (10) inhibits both ADP-driven [PP]$_2$-[^{3}H]InsP$_4$ dephosphorylation and ATP-driven [^{3}H]InsP$_6$ phosphorylation by PPIP5K2^{KD}: HPLC analysis.

A, **B**, **C**: Incubation buffers (see Methods) contained 200 nM 1,5-[PP]$_2$-[^{3}H]InsP$_4$ and either no enzyme (panel **A**), or 1 ng PPIP5K2^{KD} (panel **B**) or 1 ng PPIP5K2^{KD} plus 2 μM 2,5-di-*O*-Bn-InsP$_4$ (**10**) (panel **C**). After 20 min reactions were quenched and analyzed by HPLC (Weaver et al., 2013). After accounting for the small (11%) contamination of the [PP]$_2$-[^{3}H]InsP$_4$ with PP-[^{3}H]InsP$_5$ (panel A), reactions rates in the absence and presence of 2,5-di-*O*-Bn-InsP$_4$ (**10**) were calculated to be 192 and 95 nmol/mg protein/min, respectively. **D**, **E**:Incubation buffers (see Methods) contained 500 nM [^{3}H]InsP$_6$ plus either 12.5 ng PPIP5K2^{KD} (panel **D**) or 30 ng PPIP5K2^{KD} plus 1.5 μM 2,5-di-*O*-Bn-InsP$_4$ (**10**) (panel **E**). Reactions rates in the absence and presence of 2,5-di-*O*-Bn-InsP$_4$ (**10**) were calculated to be 52 and 21 nmol/mg protein/min, respectively.

S2, related to Figure 6. Ligand occupation of the substrate capture site.

Panel **A** illustrates the steric clashing that prevents 5-PA-InsP$_5$ (**1**) from simultaneously binding to both the active site and the substrate capture site. Panels **B** and **C** illustrate how occupation of the substrate capture site by 2-*O*-Bn-5-PA-InsP$_4$ (**2**) and 2,5-di-*O*-Bn-InsP$_4$ (**10**) respectively imposes steric constraints that prevents InsP$_6$ from binding to the catalytic site. Analogs are depicted as stick models. Atoms are colored red for oxygen and orange for phosphorus, carbons colored gray in 5-PA-InsP$_4$ (**1**), cyan in 2-*O*-Bn-5-PA-InsP$_4$ (**2**), green in 2,5-di-*O*-Bn-InsP$_4$ (**10**), and magenta in InsP$_6$. Clashing phosphate groups at C-3 and C-4 on the inositol rings are highlighted. Close contacts are depicted in dashed line with distances. Panels **D**, **E**, and **F** describe Ligplots for 5-PA-InsP$_5$ (**1**), 2-*O*-Bn-5-PA-InsP$_4$ (**2**), and 2,5-di-*O*-Bn-InsP$_4$ (**10**) respectively.

S3, related to Figure 7. Cross-eye stereo view of the spatial separation of AMP-PNP from the substrate capture site.

Protein residues are shown as stick models. AMPPNP, and inositol phosphate analogs 5-PA-InsP5 (**1**), 2-*O*-Bn-5-PA-InsP4 (**2**), and 2,5-di-*O*-Bn-InsP4 (**10**) all are shown as thicker stick models. Magnesium atoms and water molecules are shown as spheres. Atoms are colored magenta for magnesium, blue for nitrogen, red for oxygen, orange for phosphorus, and carbon for grey, cyan or green. Hydrogen bonds are shown as black dashed lines.

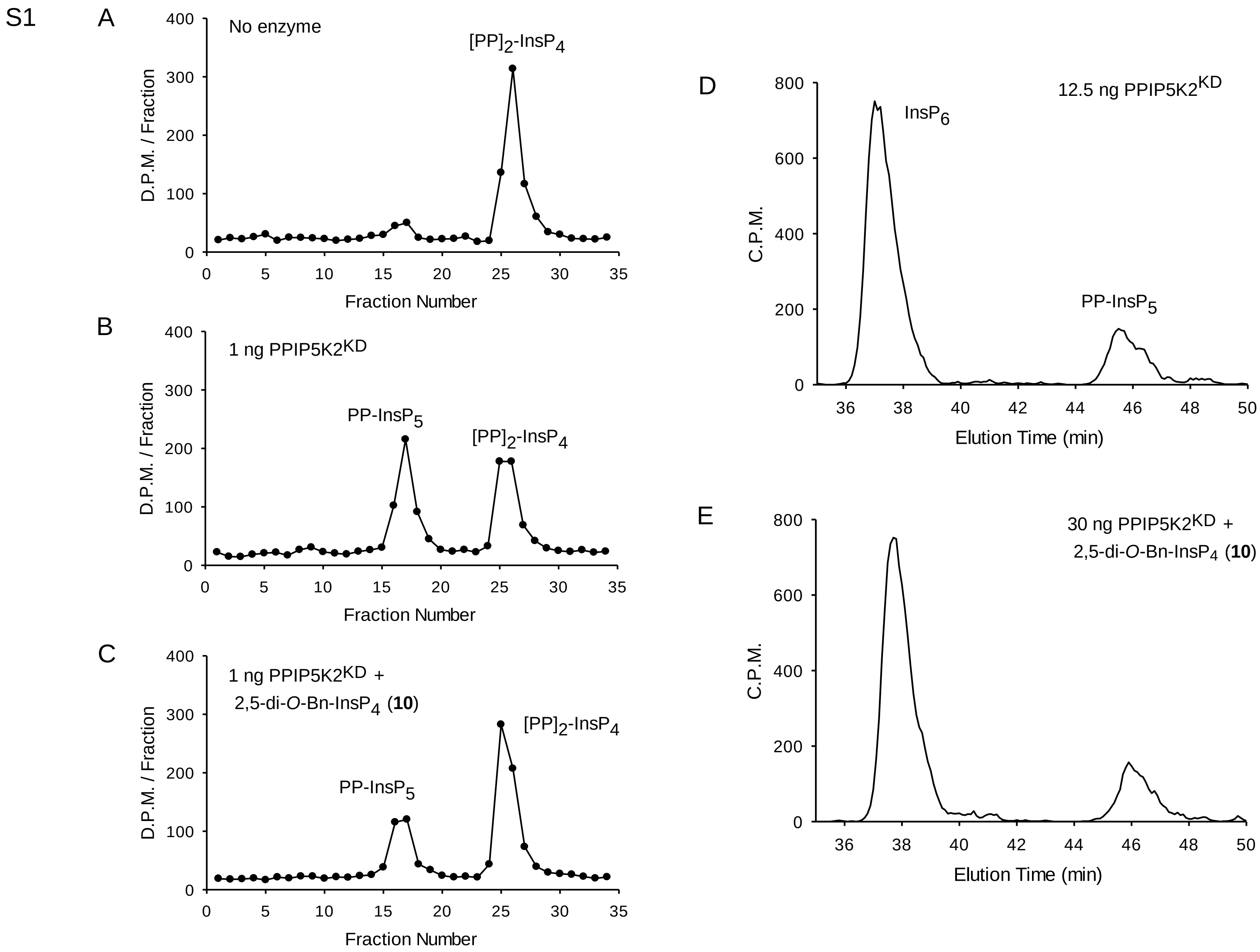
S1
A
No enzyme
[PP]2-InsP4
D.P.M. / Fraction
Fraction Number
B
1 ng PPIP5K2KD
PP-InsP5
[PP]2-InsP4
D.P.M. / Fraction
Fraction Number
C
1 ng PPIP5K2KD +
2,5-di-O-Bn-InsP4 (10)
[PP]2-InsP4
PP-InsP5
D.P.M. / Fraction
Fraction Number
D
12.5 ng PPIP5K2KD
InsP6
PP-InsP5
C.P.M.
Elution Time (min)
E
30 ng PPIP5K2KD +
2,5-di-O-Bn-InsP4 (10)
C.P.M.
Elution Time (min)

S2

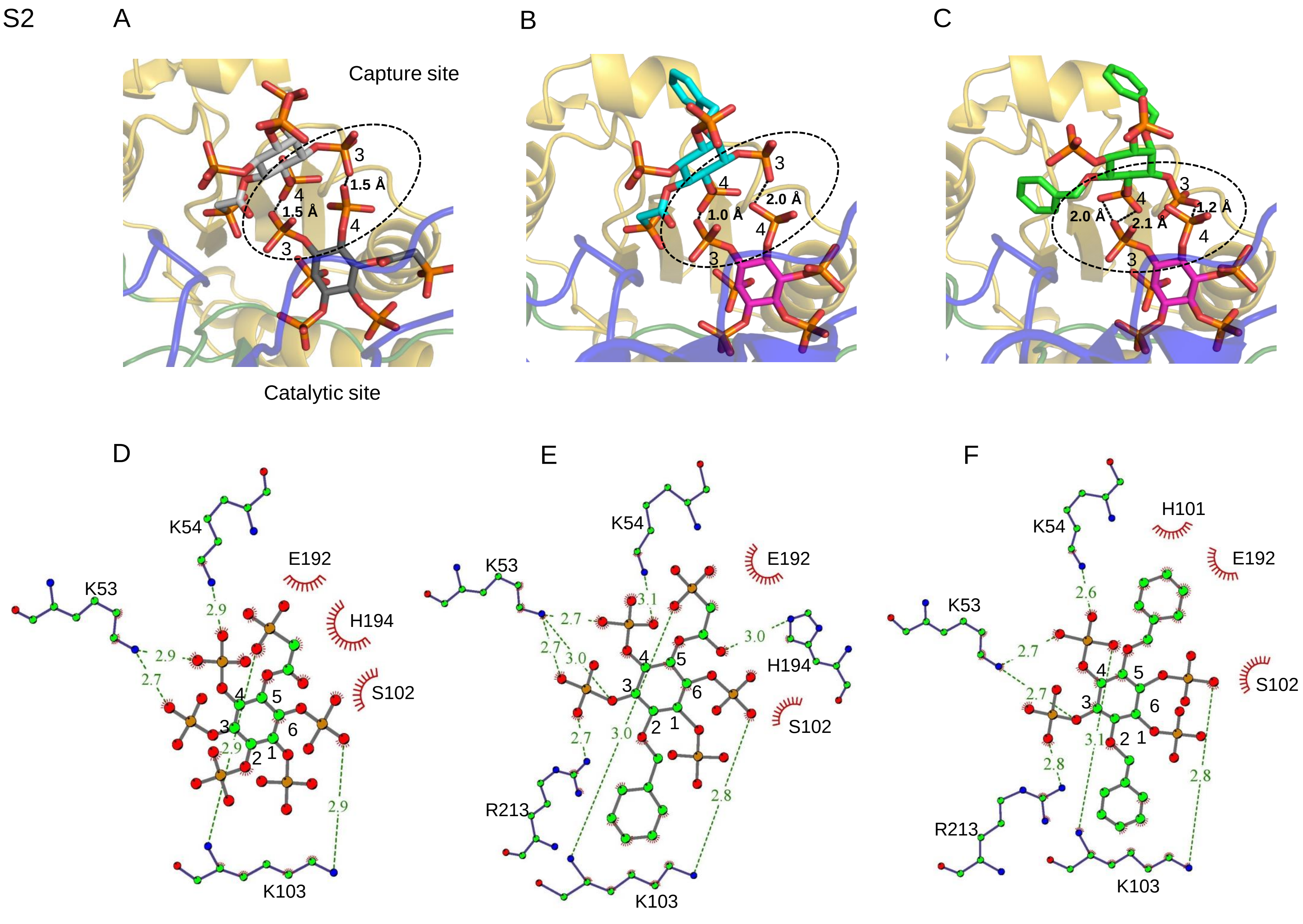

5-PA-InsP$_5$ (**1**)

2-*O*-Bn-5-PA-InsP$_4$ (**2**)

2,5-di-*O*-Bn-InsP$_4$ (**10**)

S3

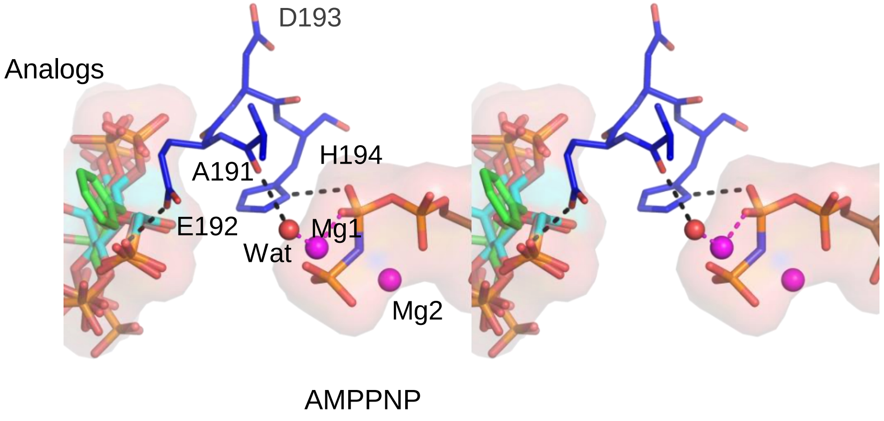

^{1}H NMR of Compound **16** (400 MHz, $CDCl_3$)

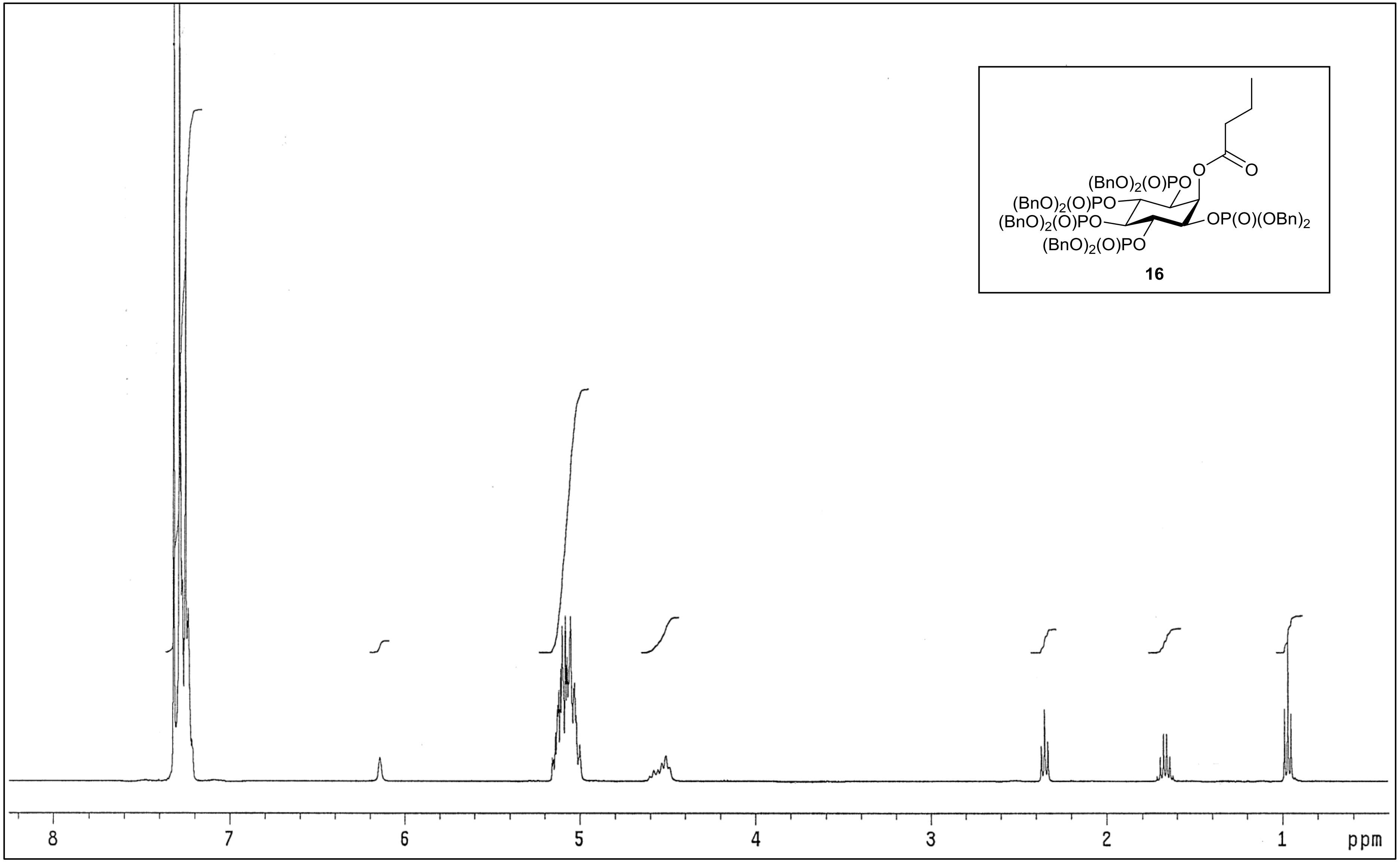

^{31}P NMR of Compound **16** (162 MHz, $CDCl_3$)

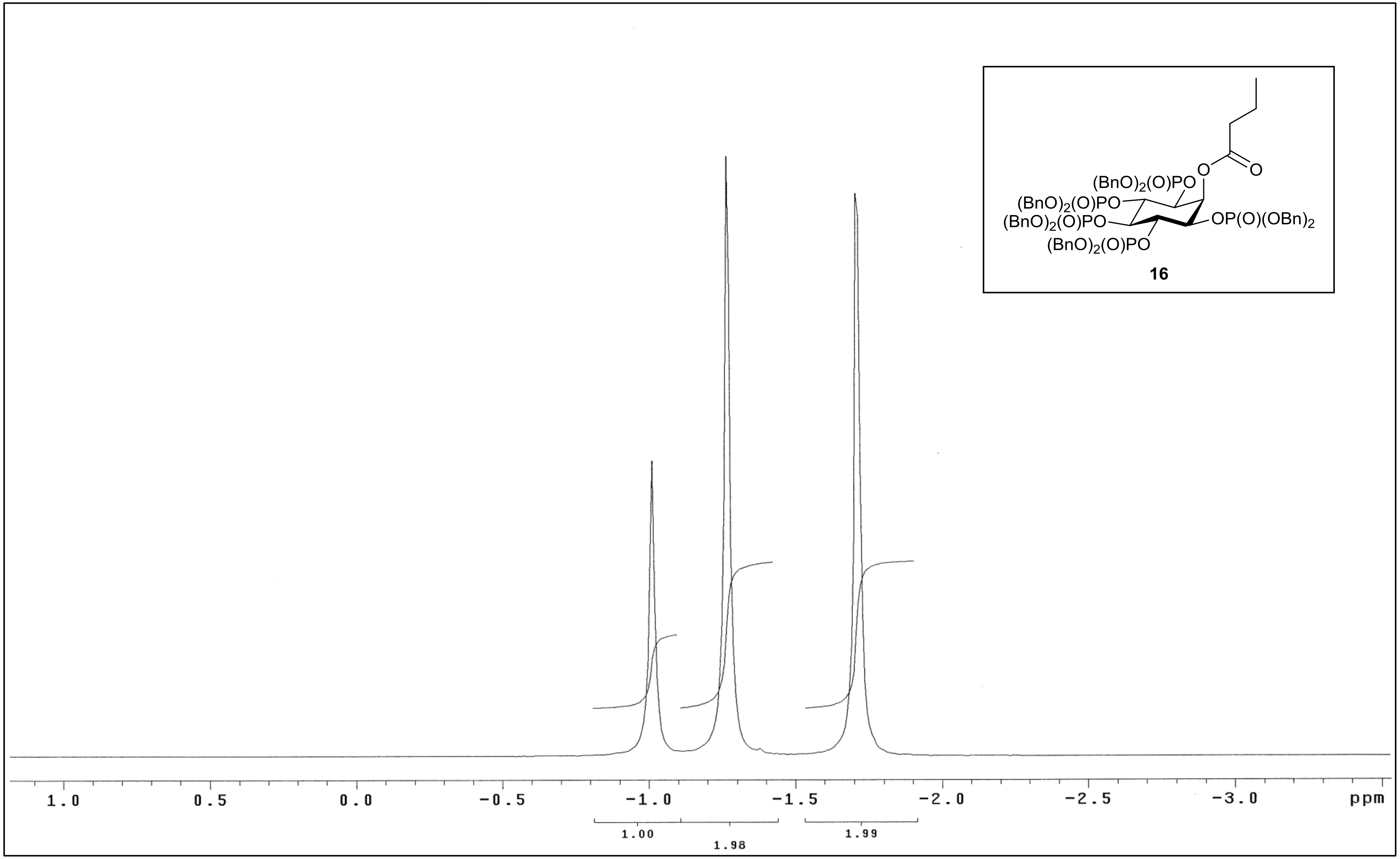

^{13}C NMR of Compound **16** (101 MHz, $CDCl_3$)

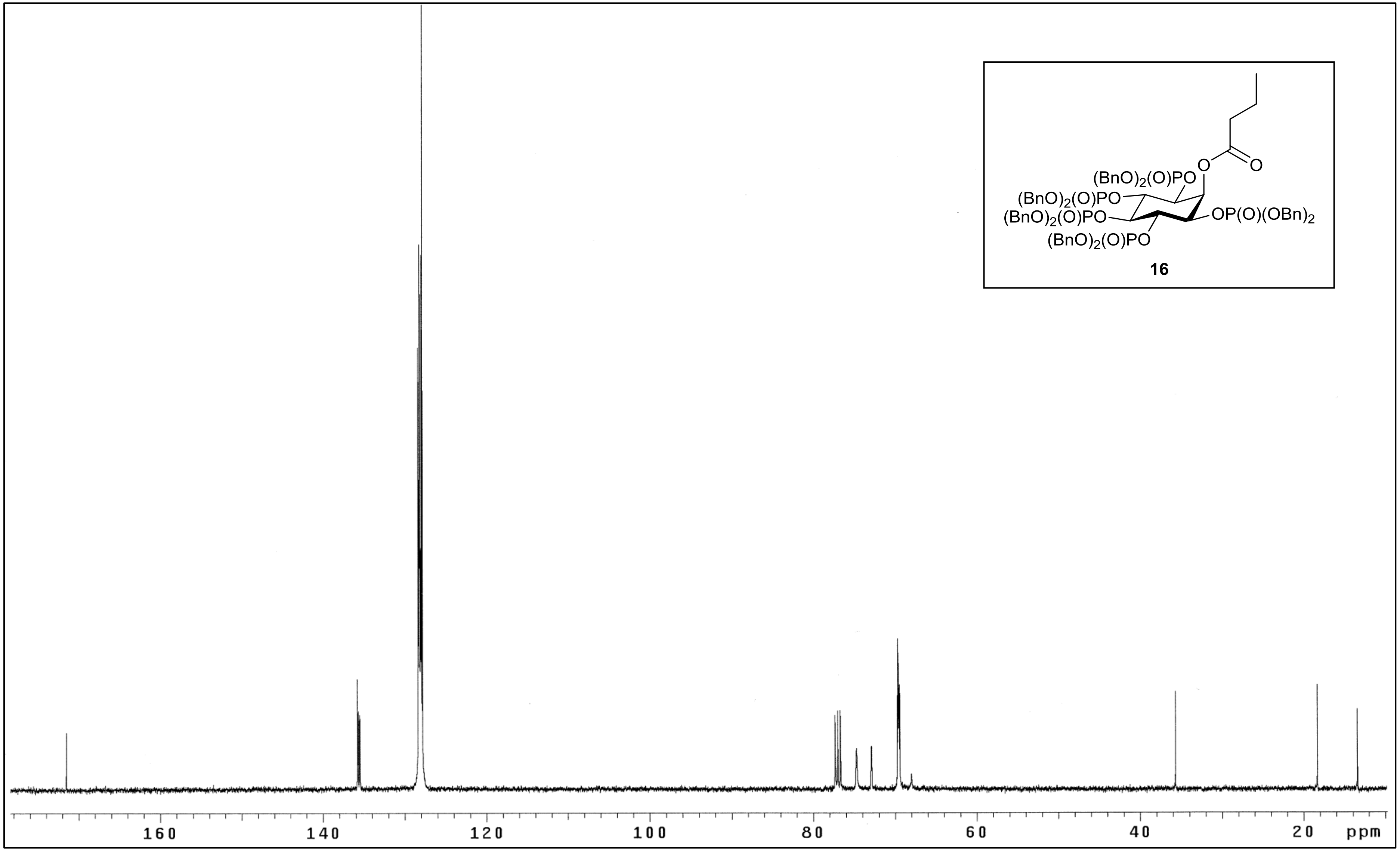

^{1}H NMR of Compound **5** (400 MHz, D_2O)

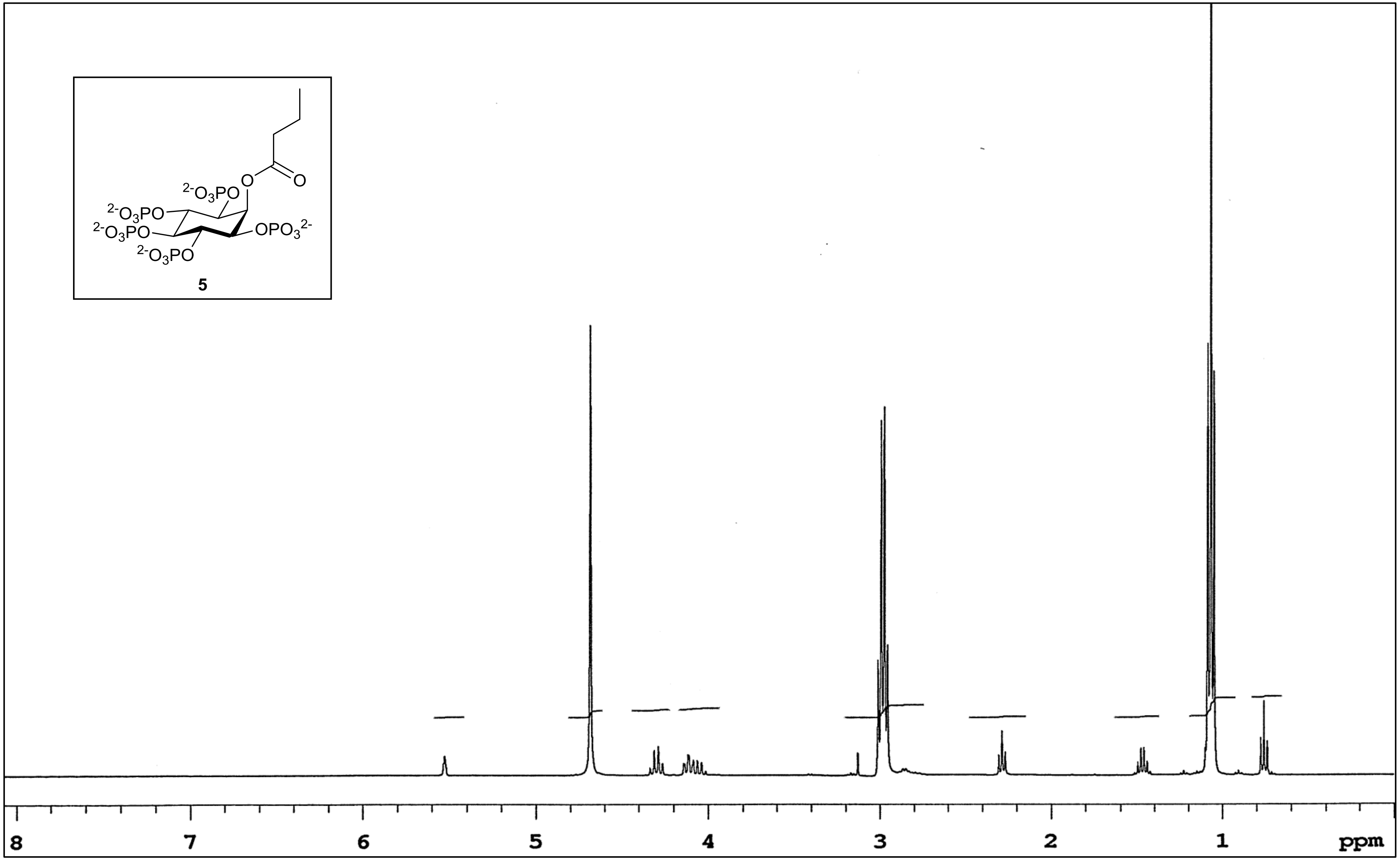

^{31}P NMR of Compound **5** (109 MHz, D_2O)

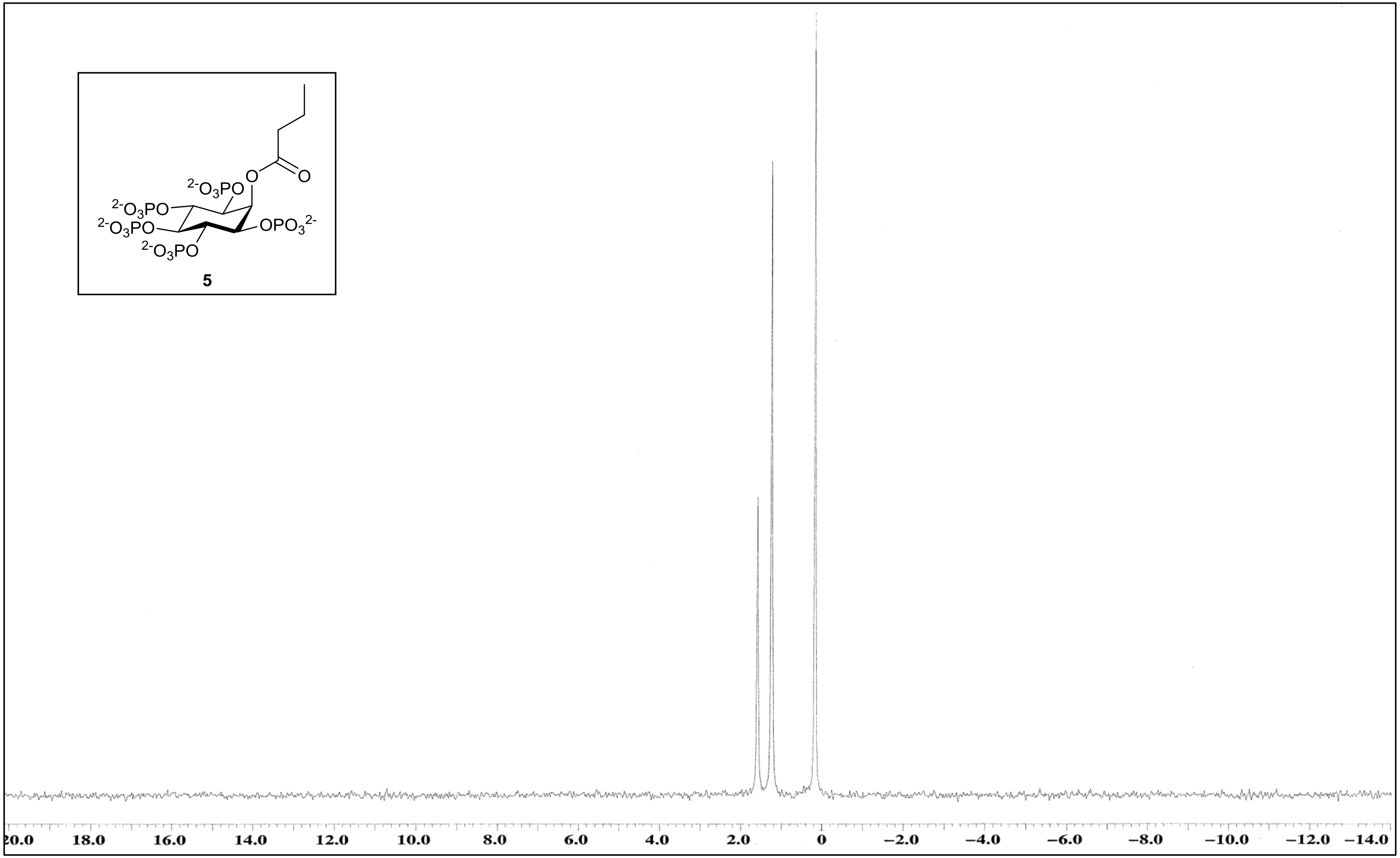

^{13}C NMR of Compound **5** (101 MHz, D_2O)

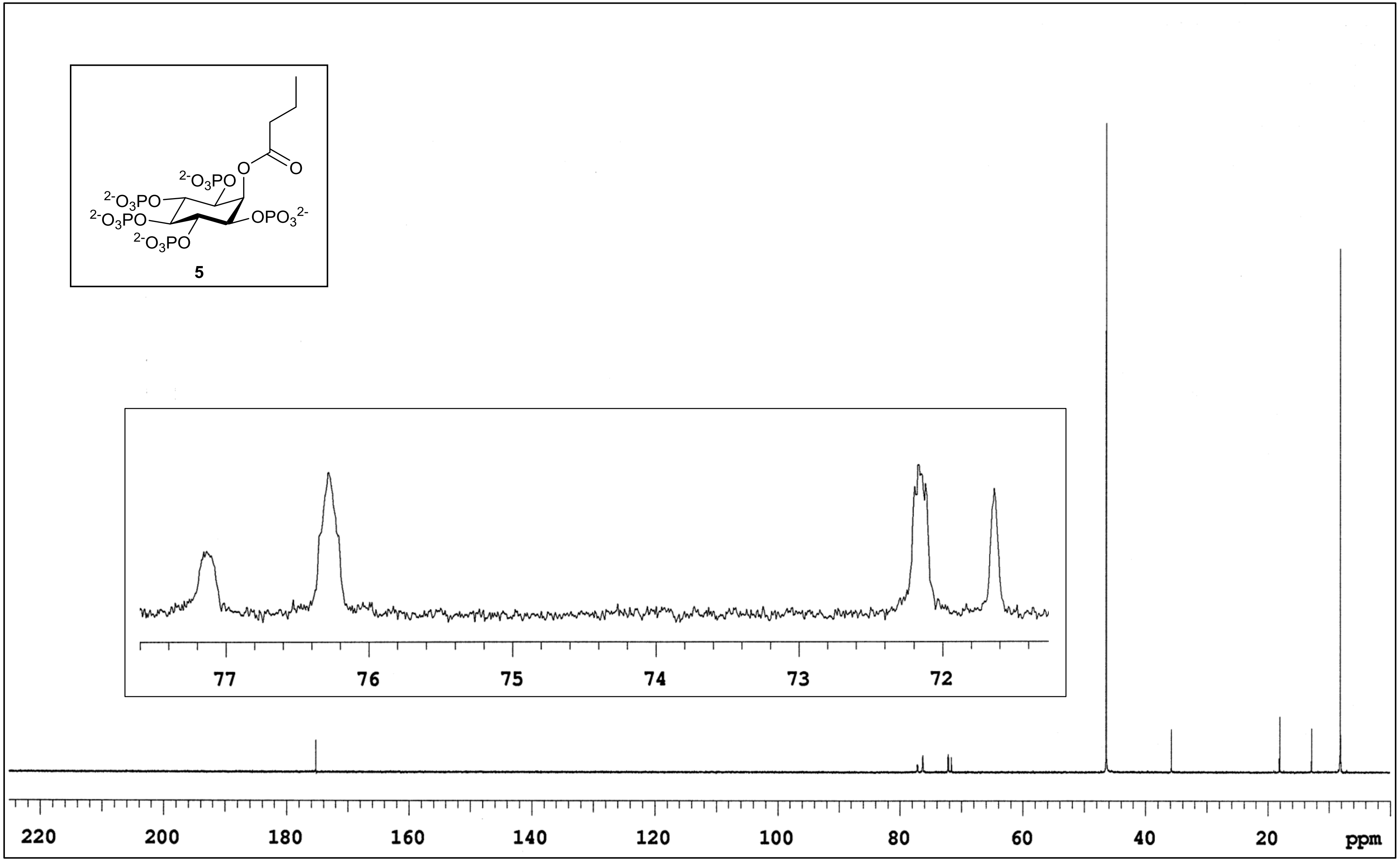

^{1}H NMR of Compound **19** (400 MHz, DMF-d_7)

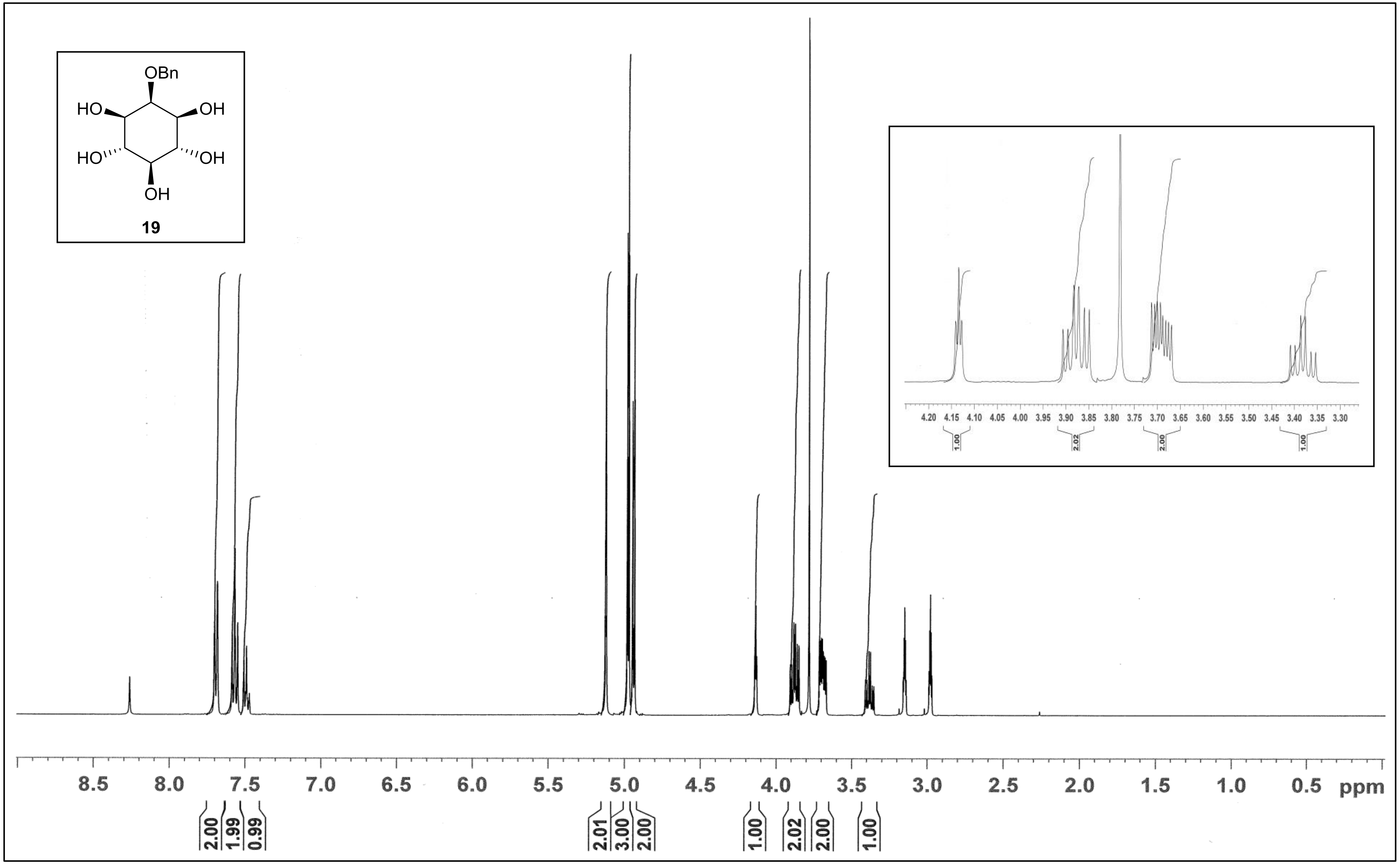

^{13}C NMR of Compound **19** (101 MHz, DMF-d_7)

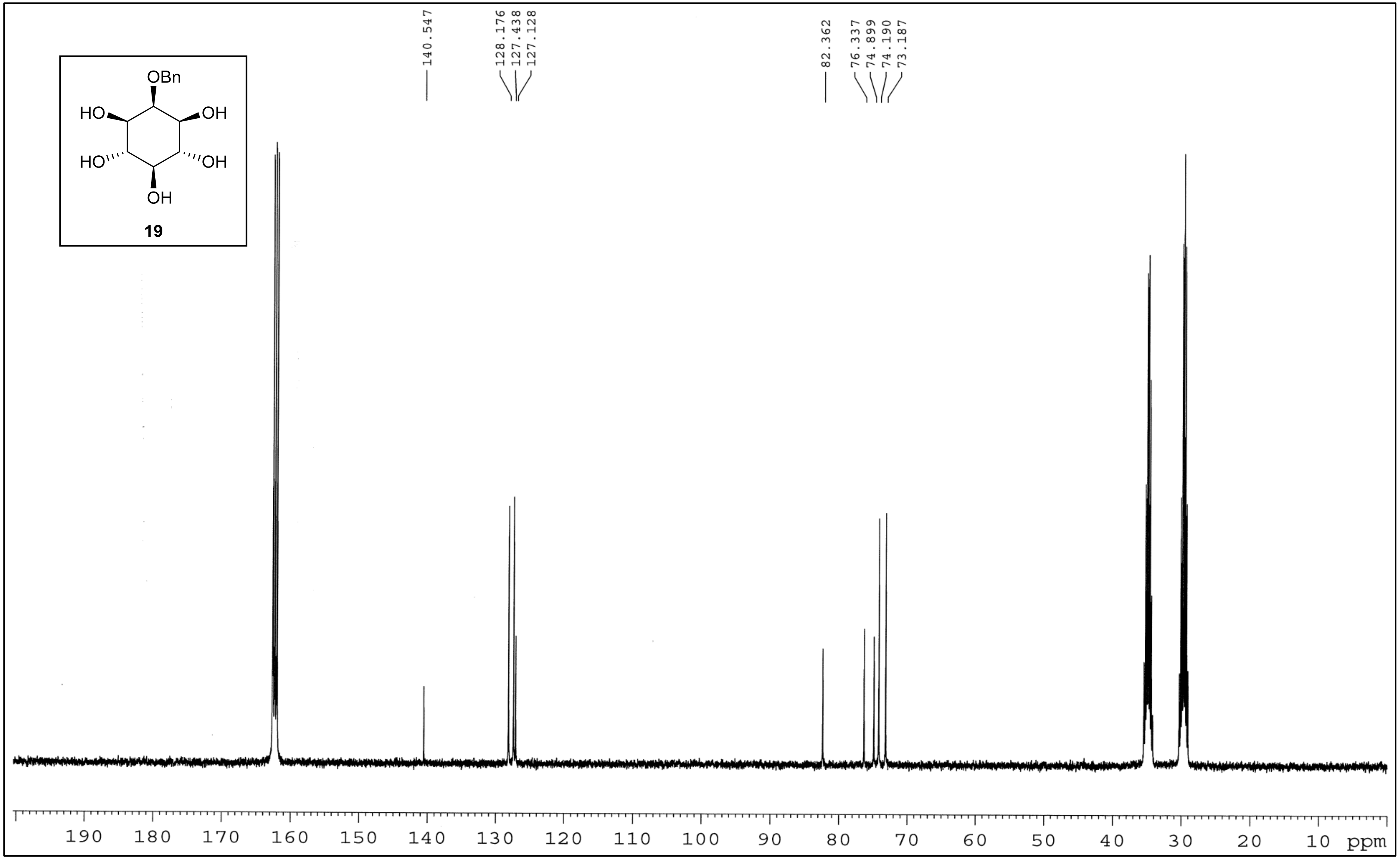

^{1}H NMR of Compound **20** (400 MHz, $CDCl_3$)

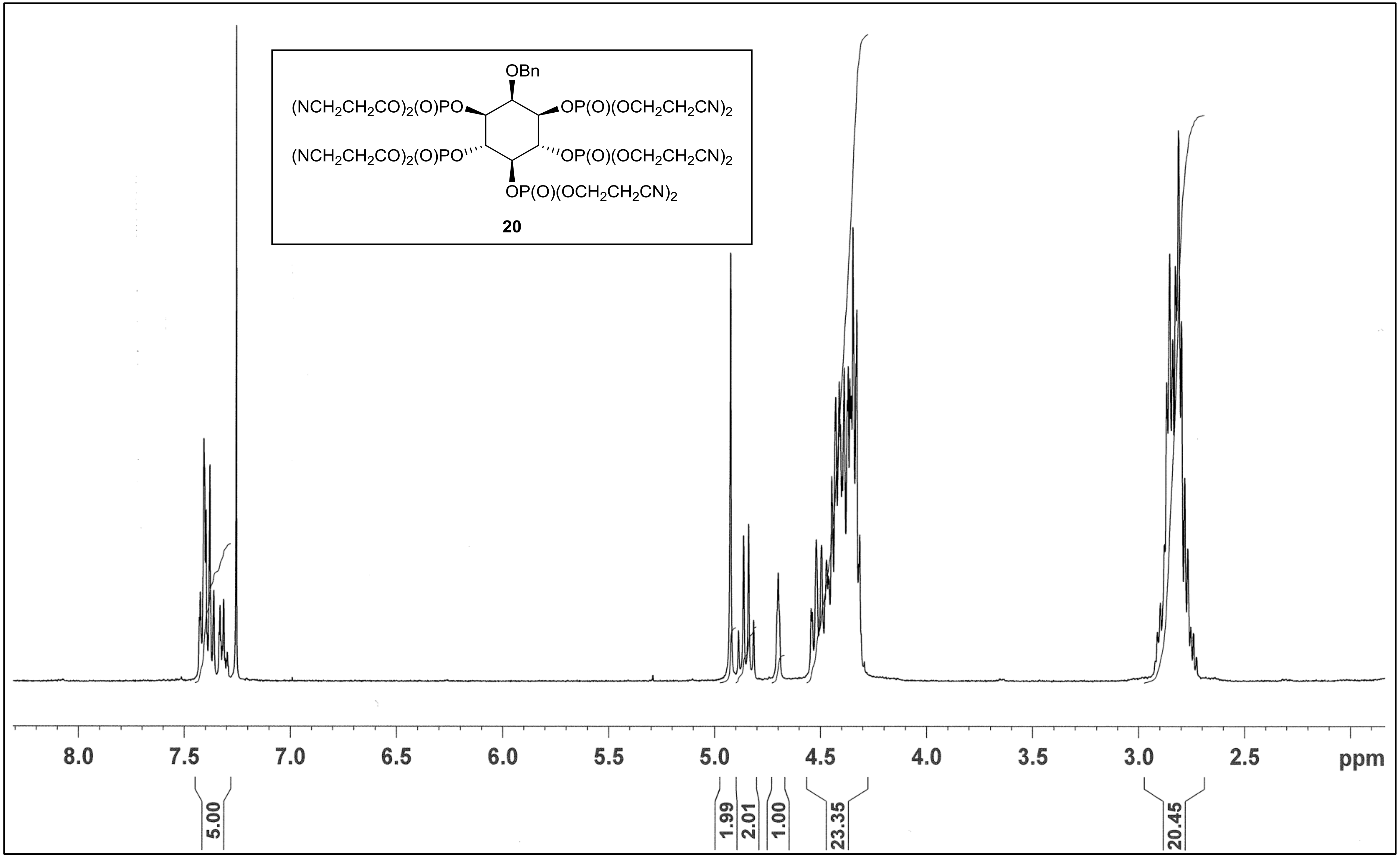

^{31}P NMR of Compound **20** (162 MHz, $CDCl_3$)

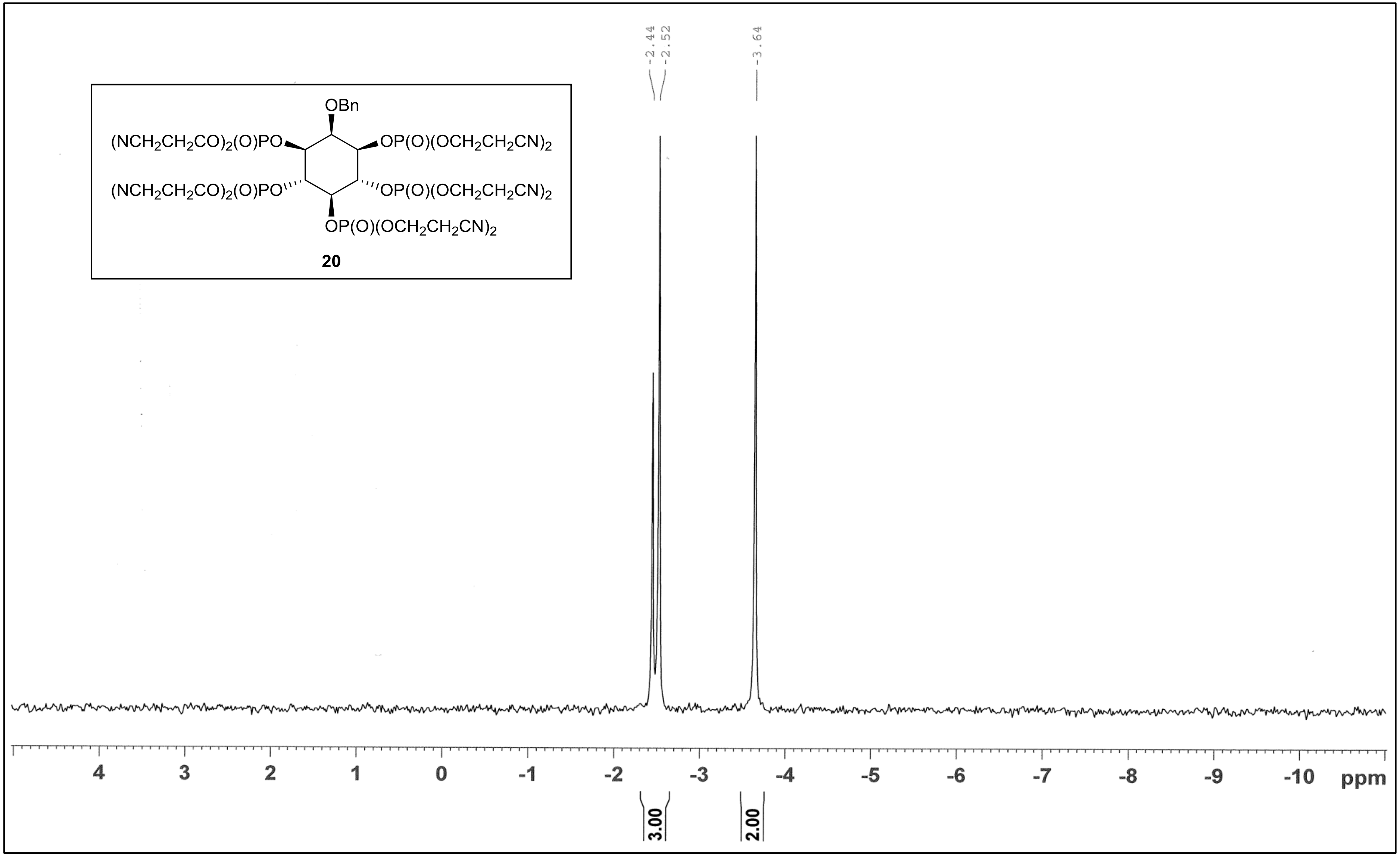

^{13}C NMR of Compound **20** (101 MHz, ACETONE-d_6)

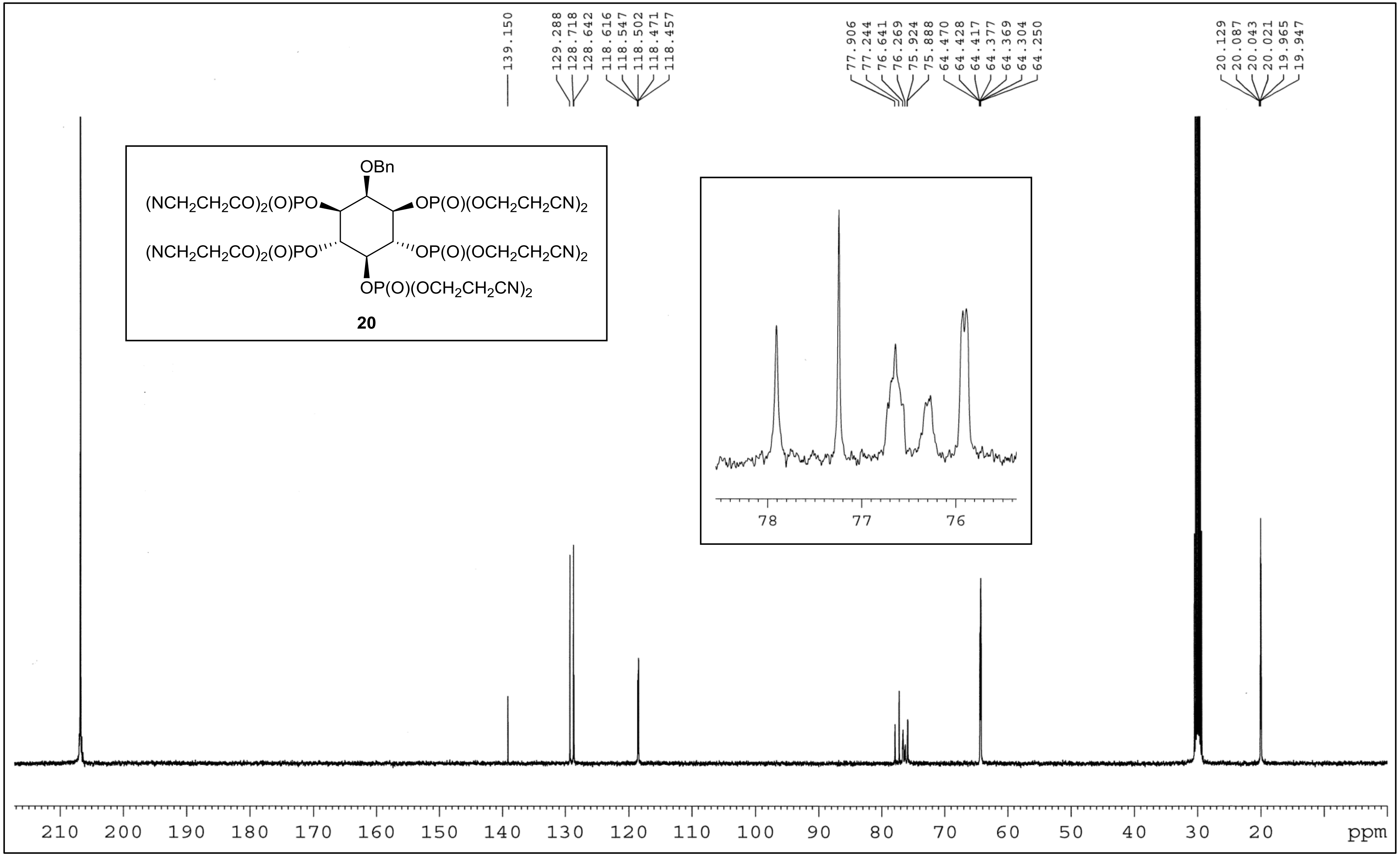

^{1}H NMR of Compound **7** (400 MHz, D_2O)

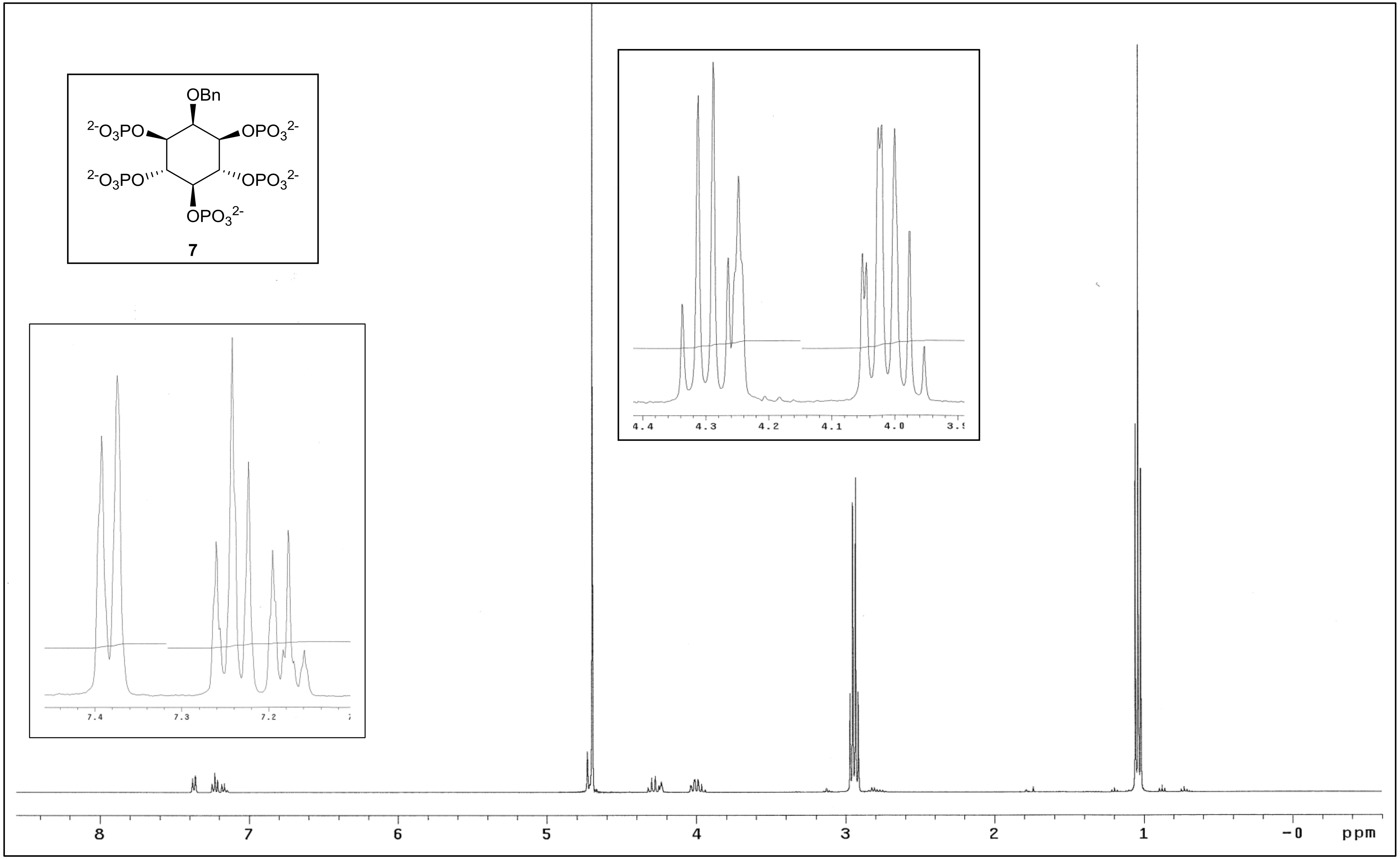

^{31}P NMR for Compound **7** (162 MHz, D_2O)

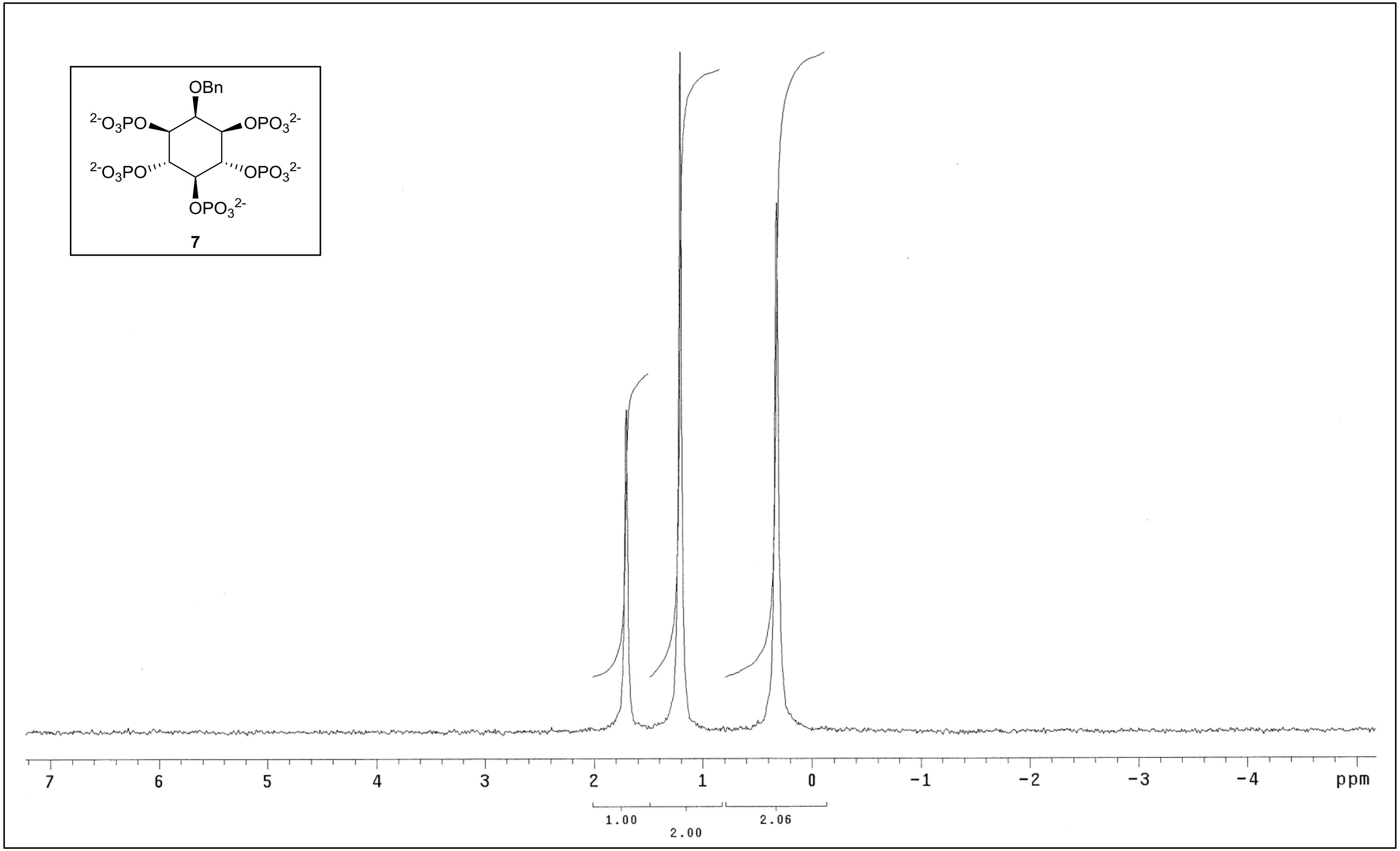

^{13}C NMR of Compound **7** (101 MHz, D_2O)

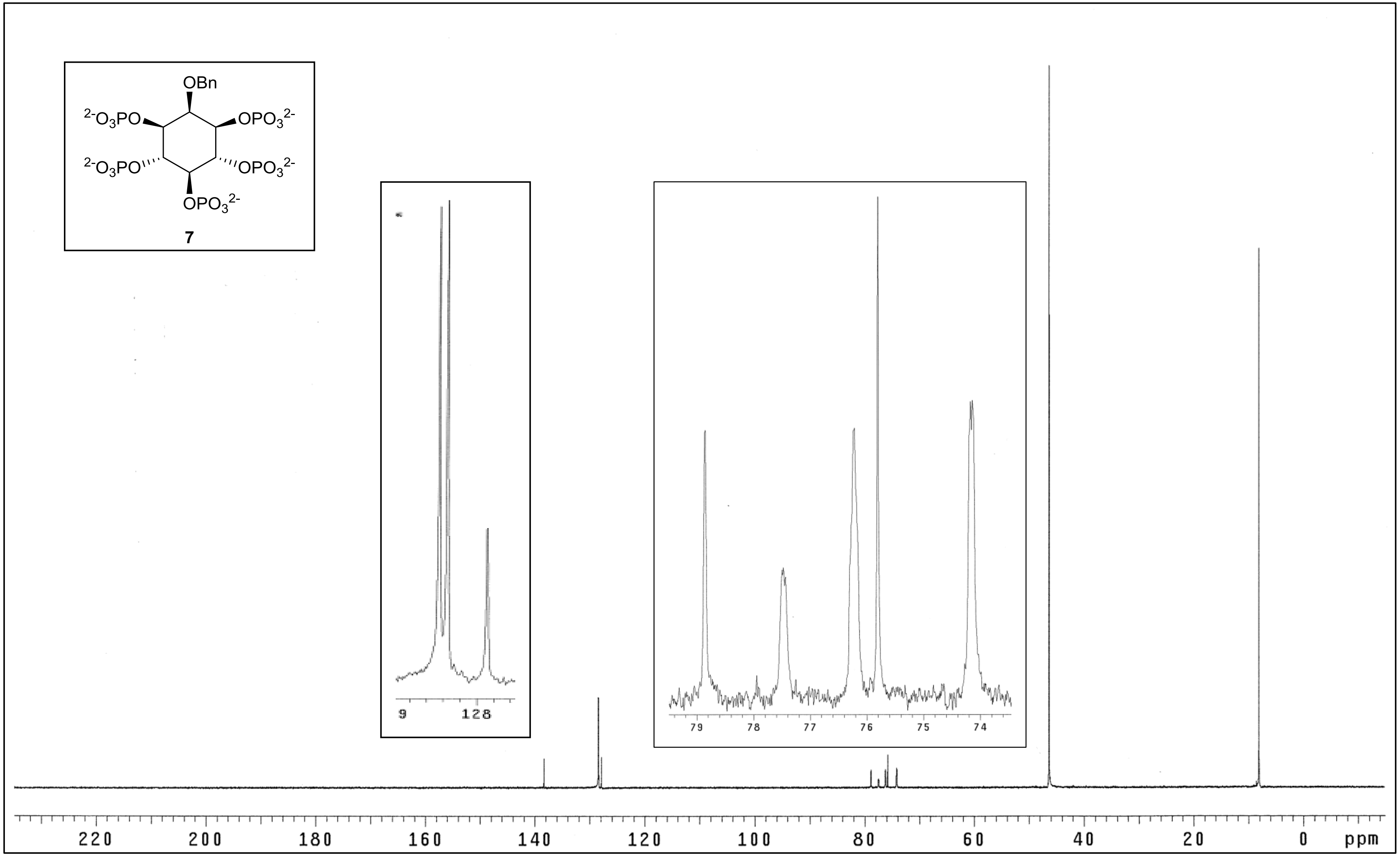

^{1}H NMR of Compound **21** (400 MHz, $CDCl_3$)

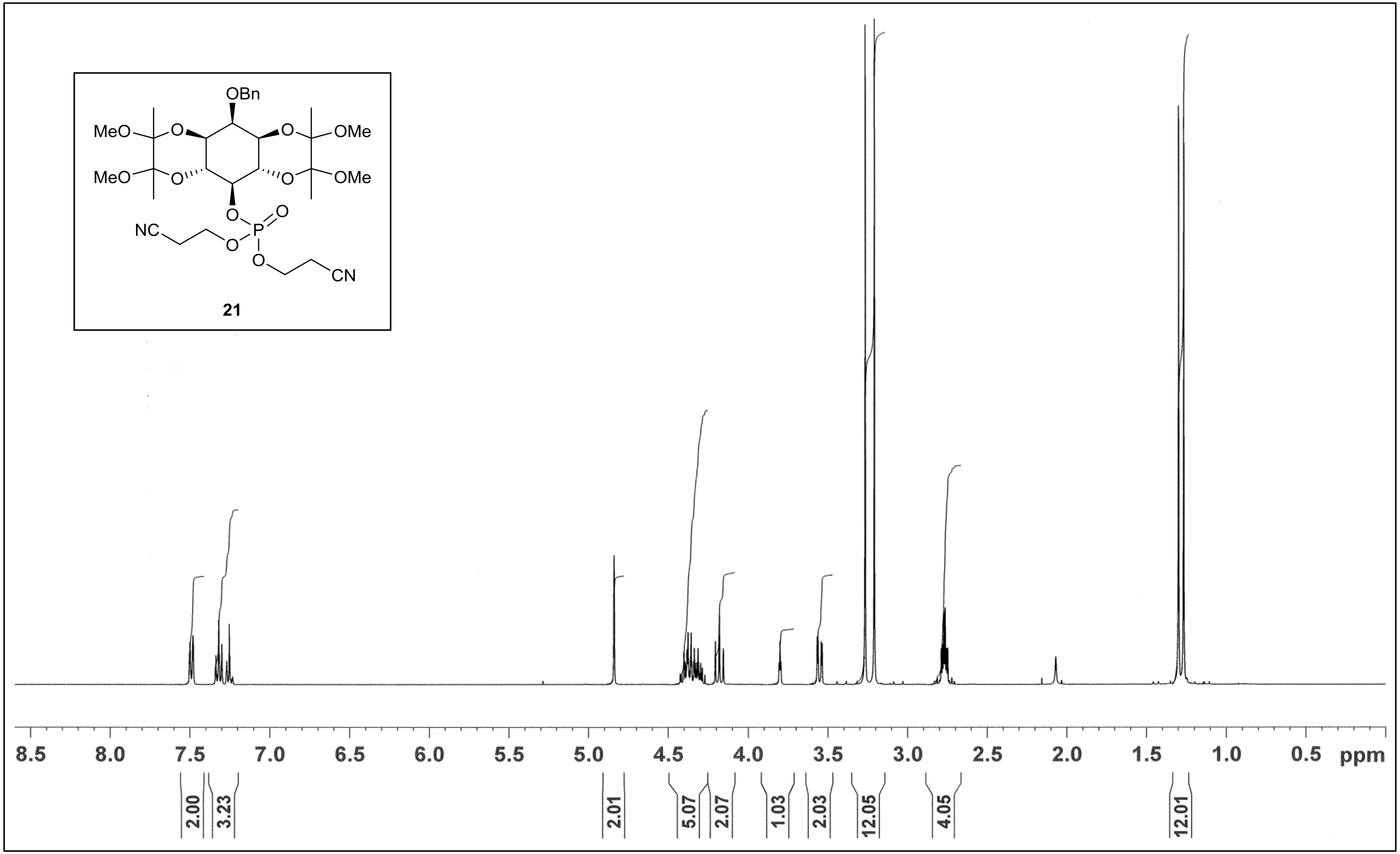

^{31}P NMR of Compound **21** (162 MHz, $CDCl_3$)

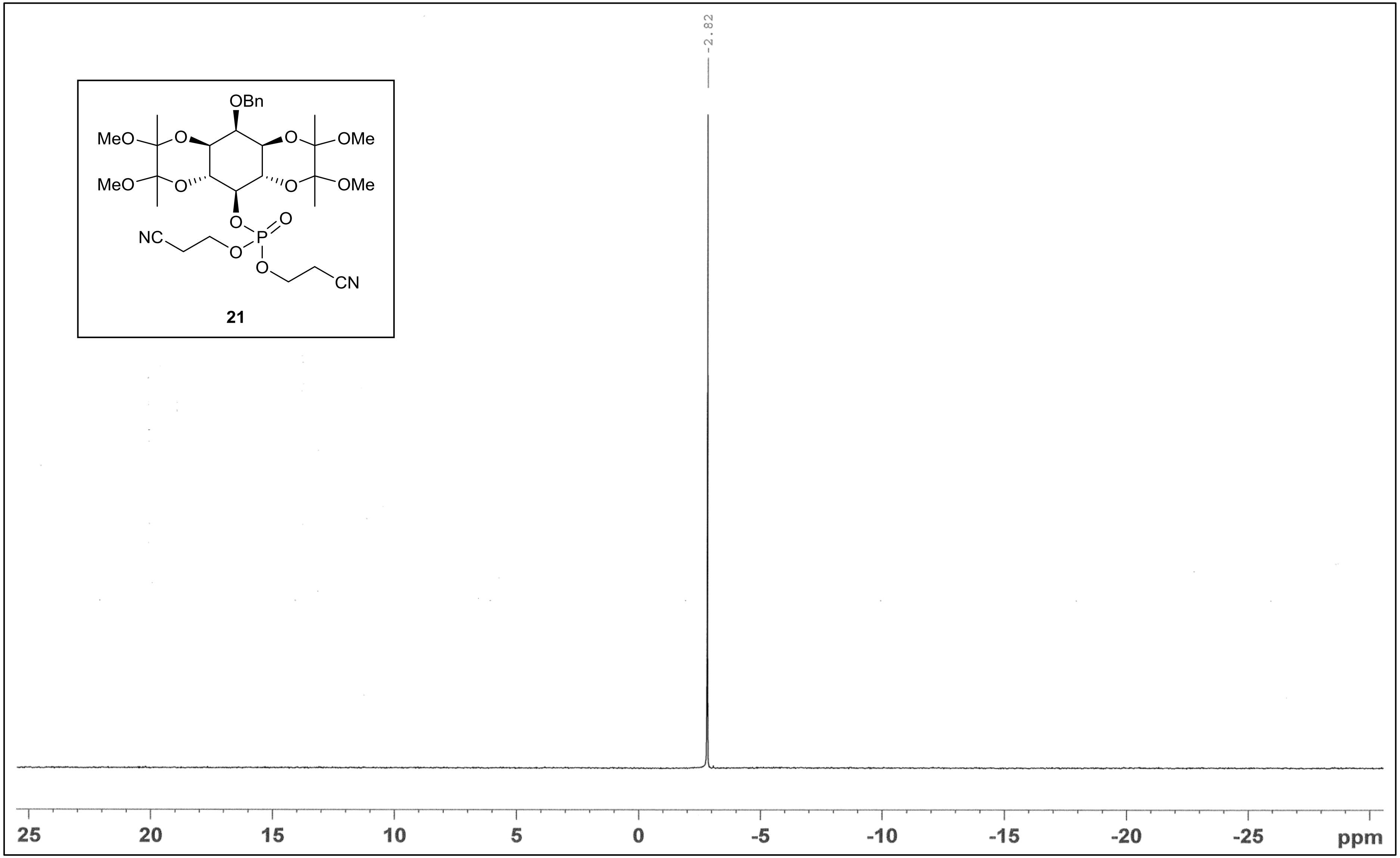

^{13}C NMR of Compound **21** (101 MHz, $CDCl_3$)

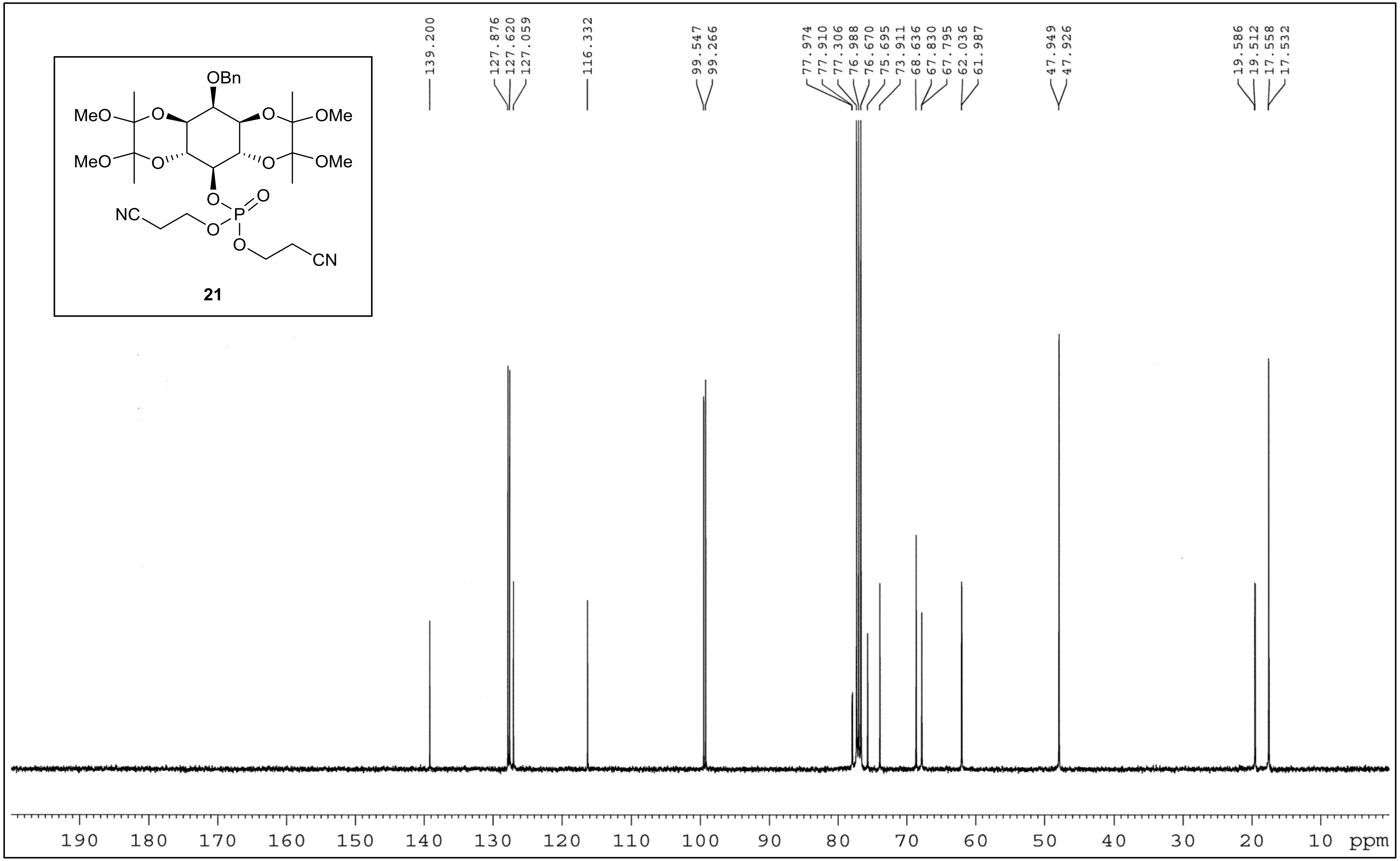

^{1}H NMR of Compound **22** (400 MHz, $CDCl_3$)

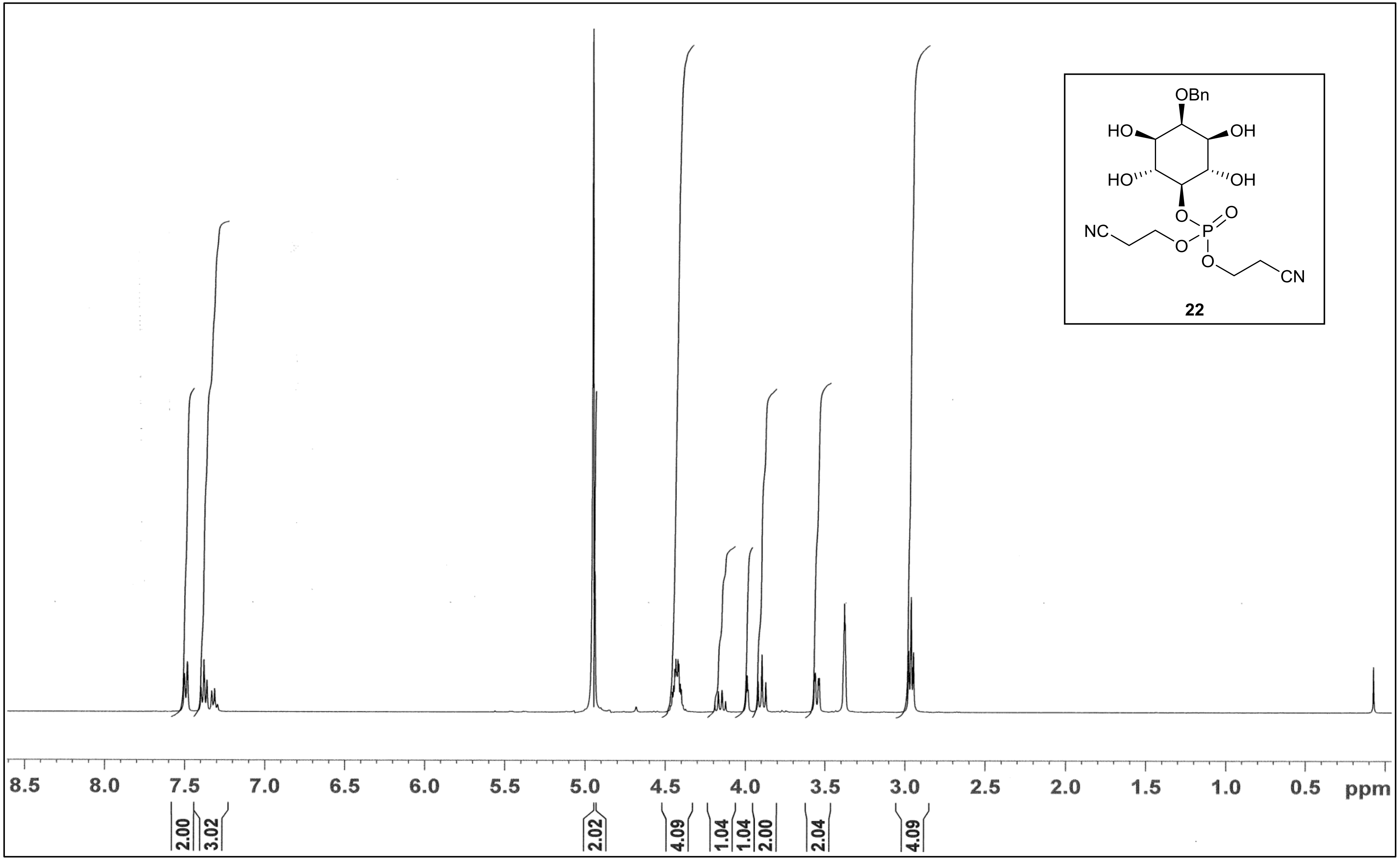

^{31}P NMR of Compound **22** (162 MHz, $CDCl_3$)

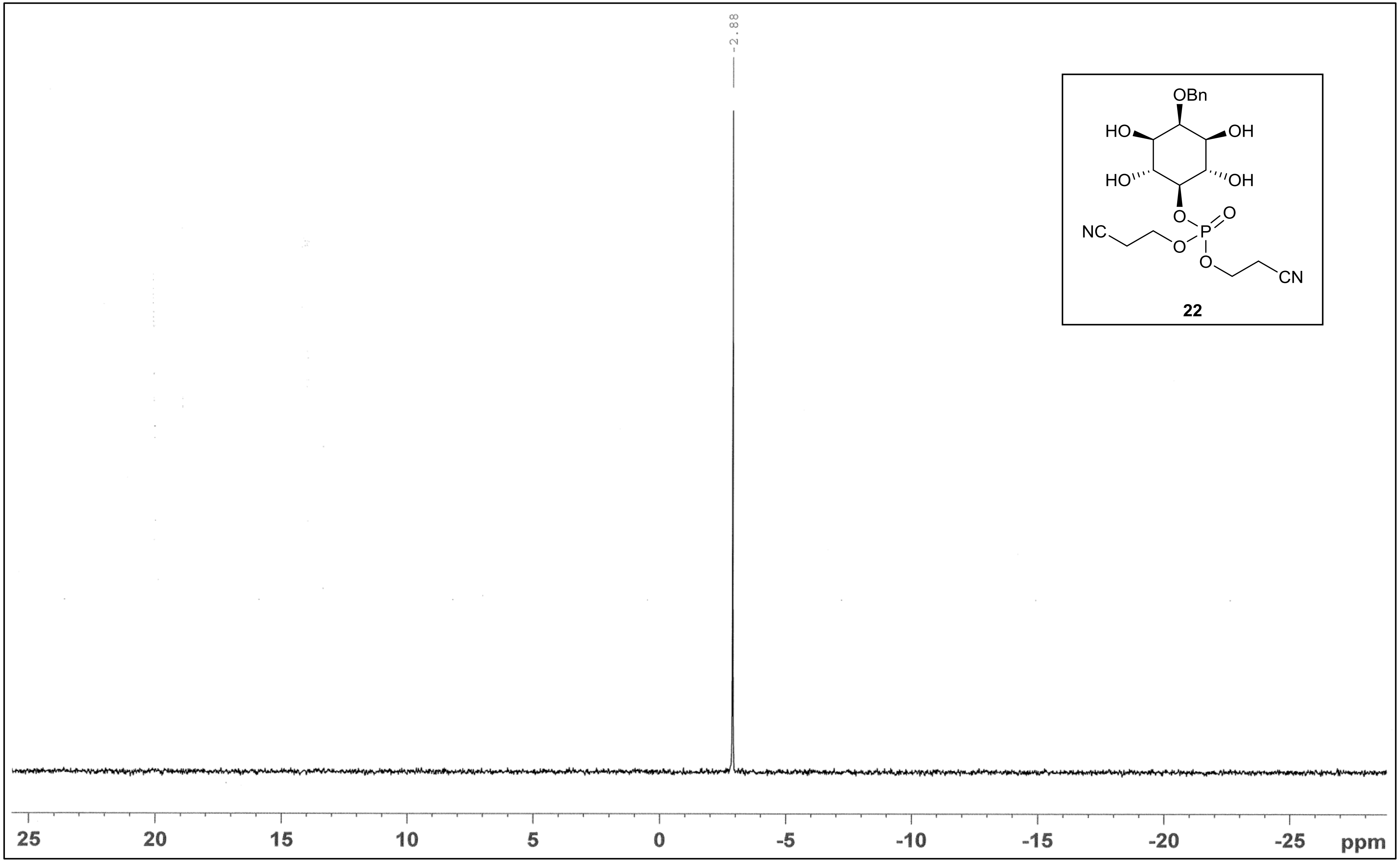

^{13}C NMR of Compound **22** (101 MHz, $CDCl_3$)

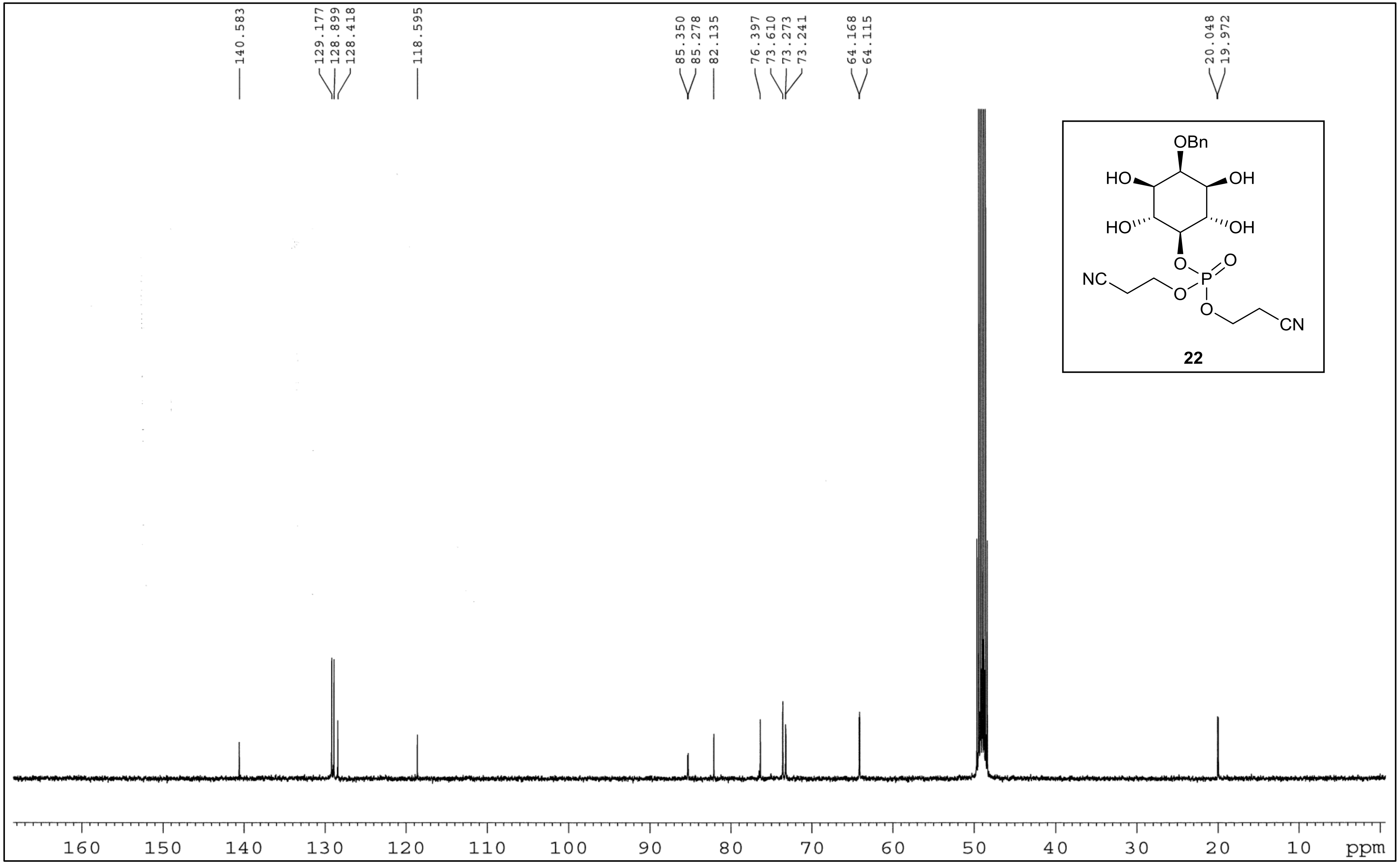

^{1}H NMR of Compound **23** (400 MHz, $CDCl_3$)

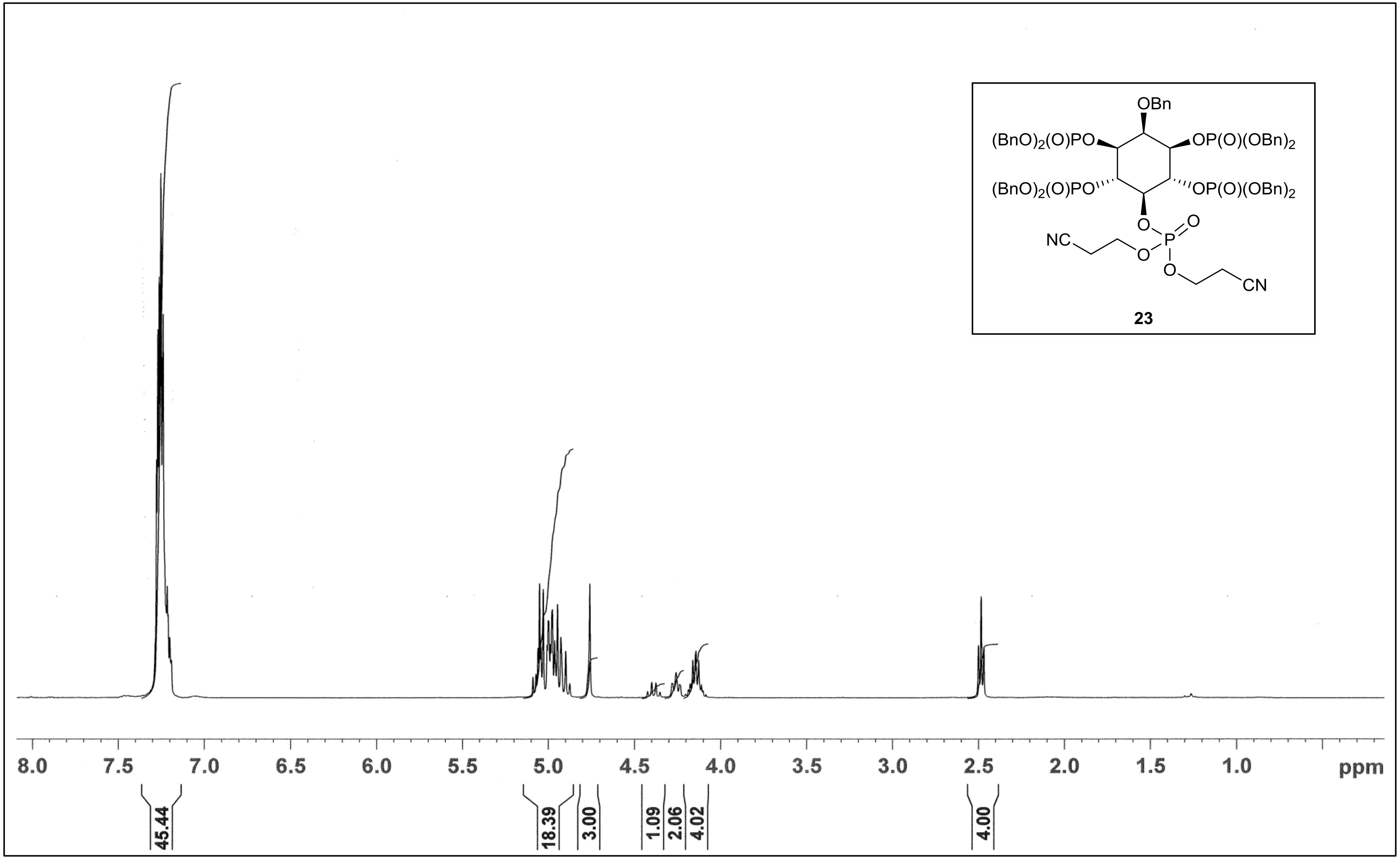

^{31}P NMR of Compound **23** (162 MHz, $CDCl_3$)

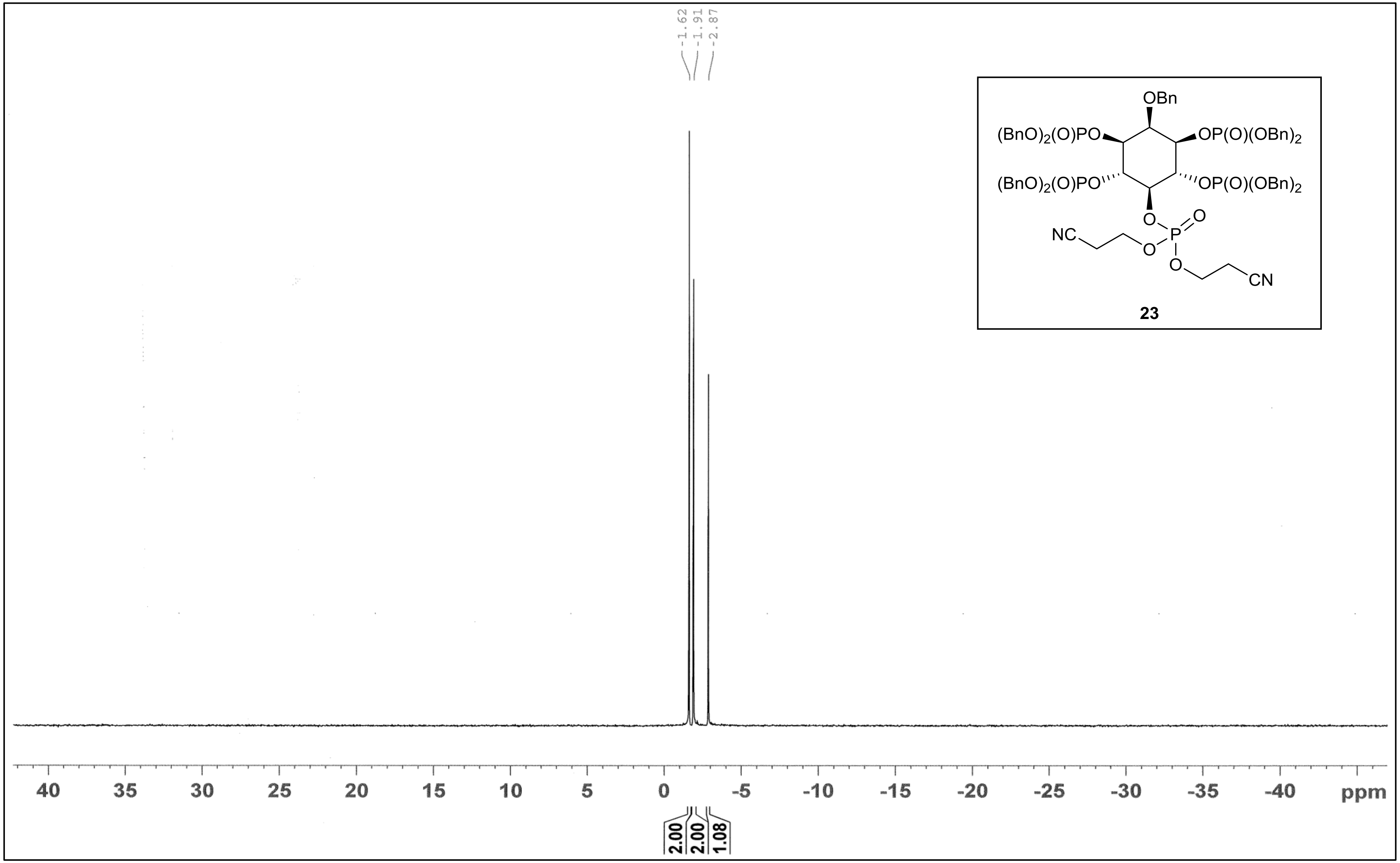

^{13}C NMR of Compound **23** (101 MHz, $CDCl_3$)

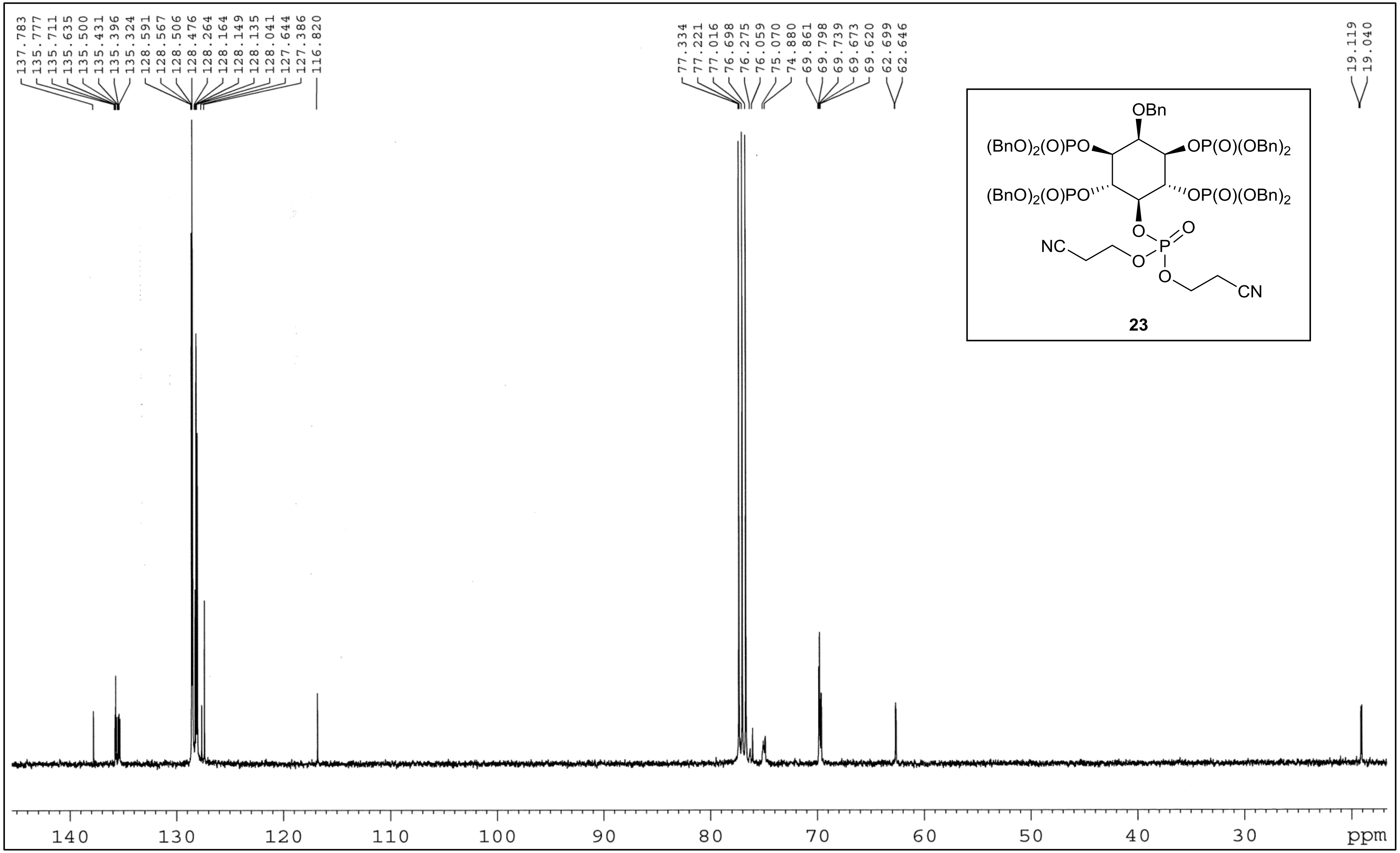

^{31}P NMR of reaction progression in the formation of Compound **23a** (162 MHz, $CDCl_3$)

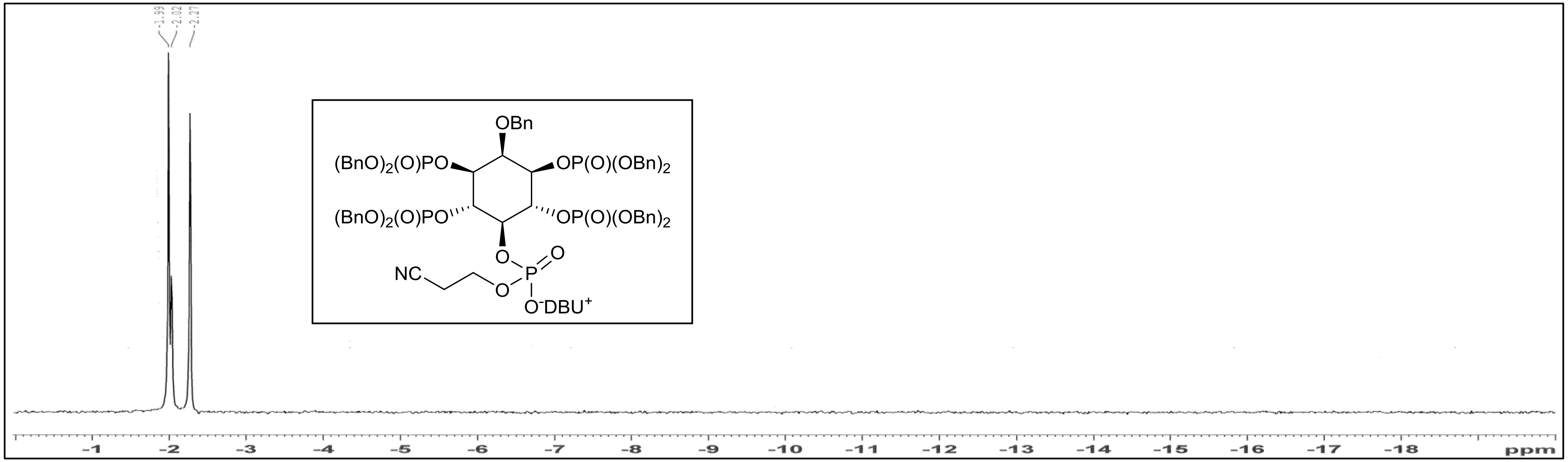

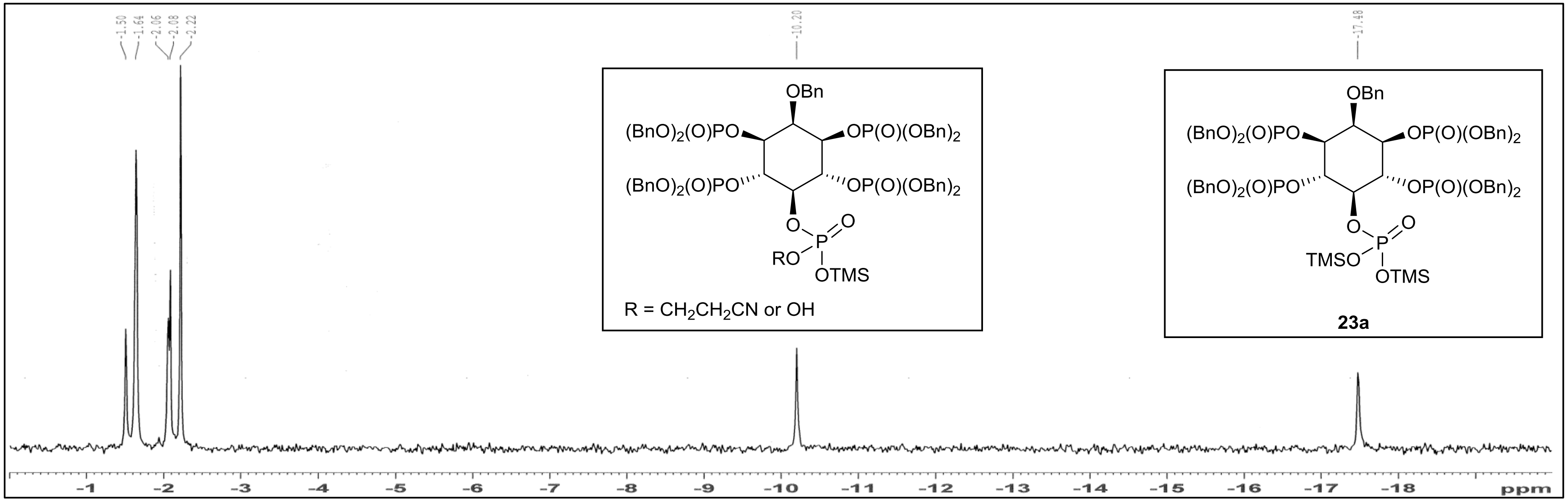

^{31}P NMR of Compound **23a** (162 MHz, $CDCl_3$)

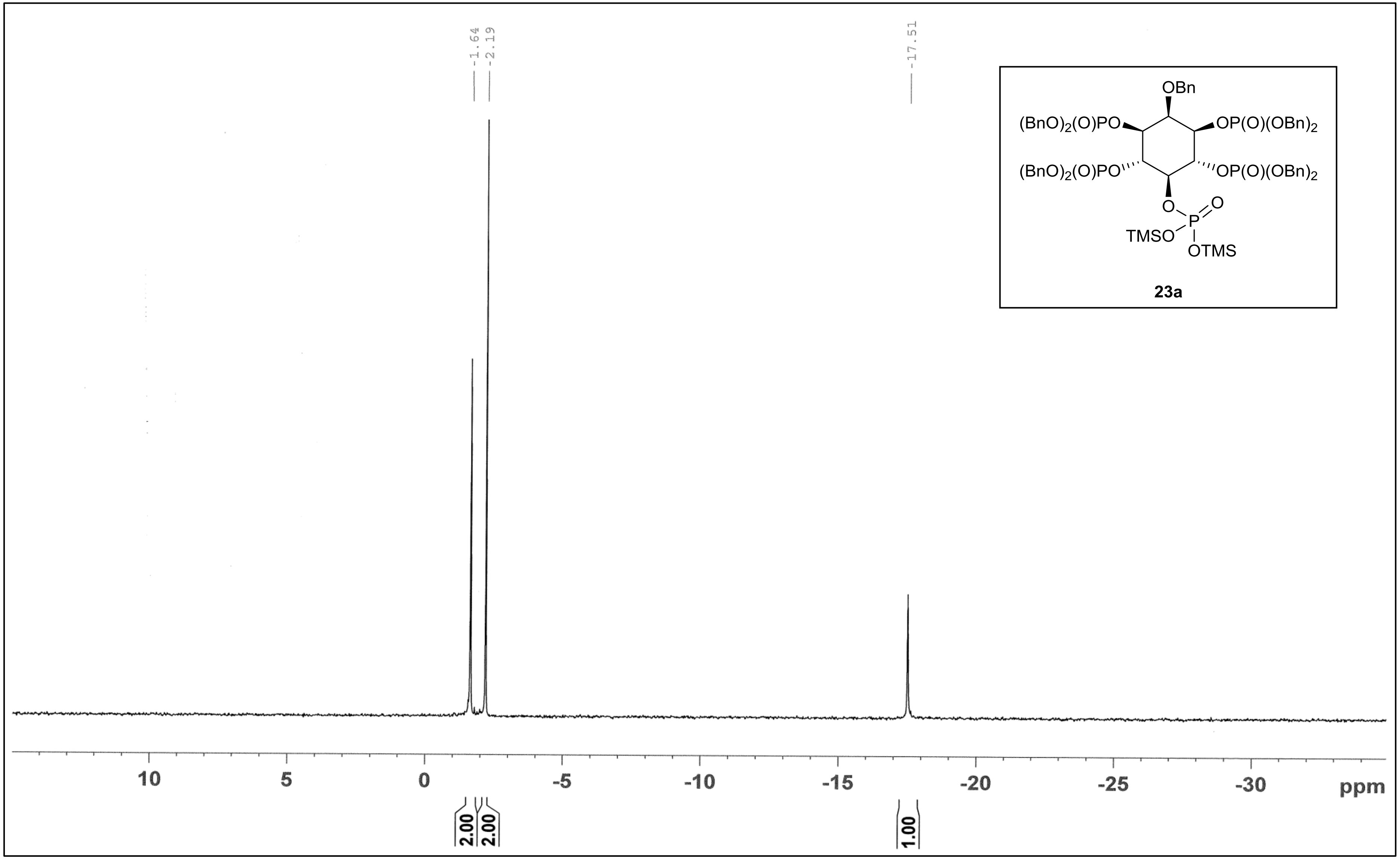

^{31}P NMR of Compound **23b** (162 MHz, $CDCl_3$)

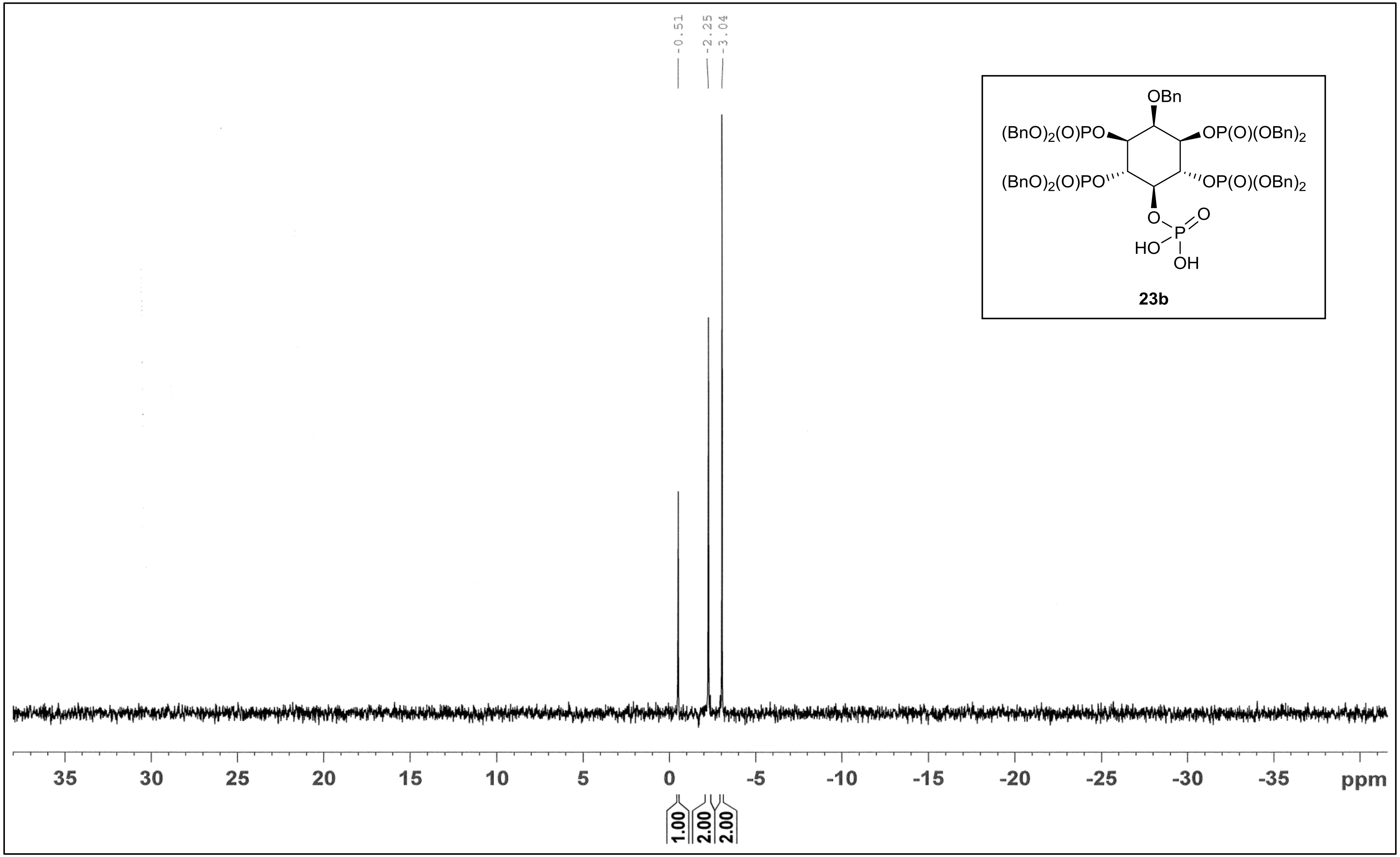

^{31}P NMR of reaction mixture in the formation of Compound **23c** (162 MHz, $CDCl_3$)

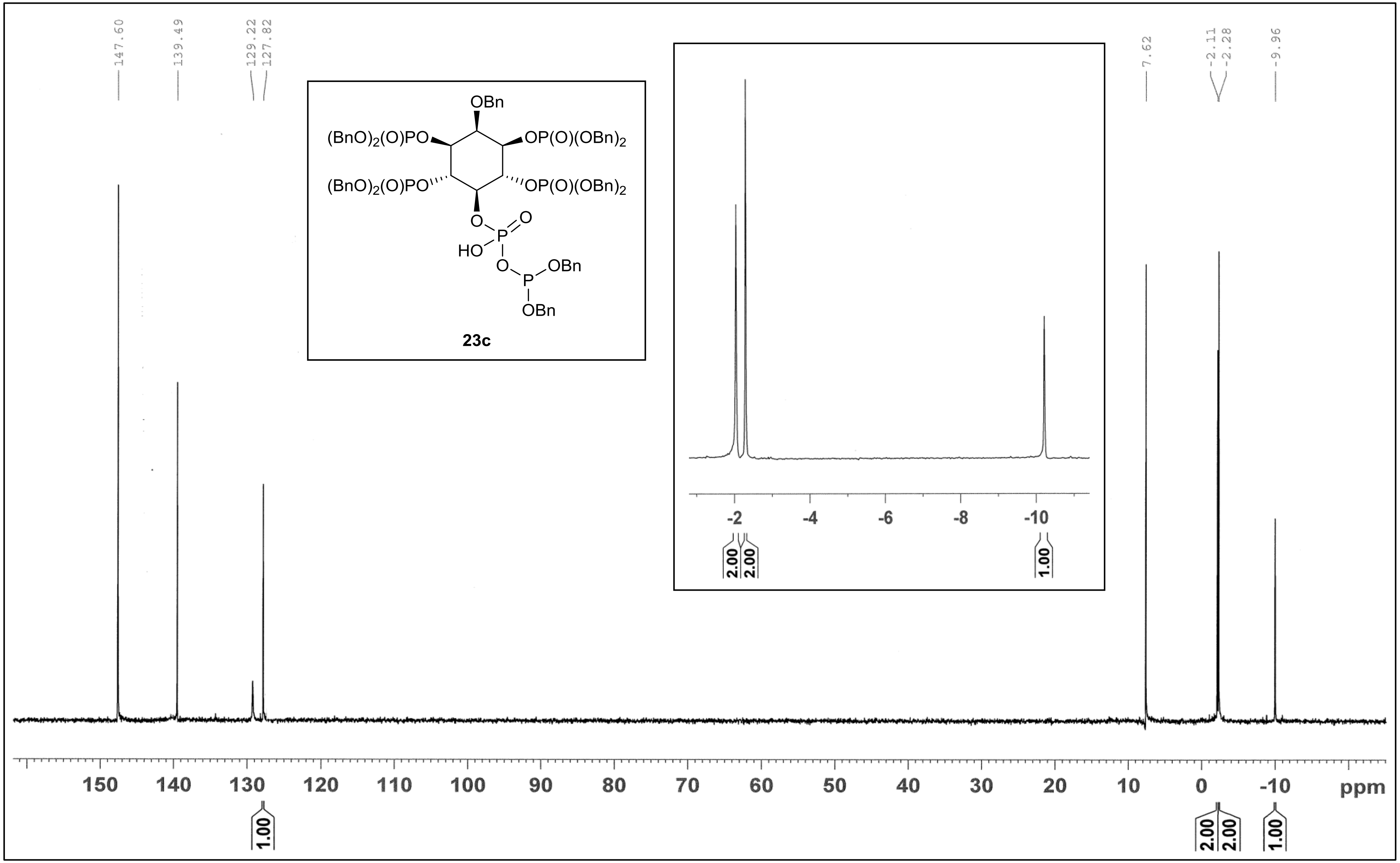

^{31}P NMR of reaction mixture in the formation of Compound **24** (162 MHz, $CDCl_3$)

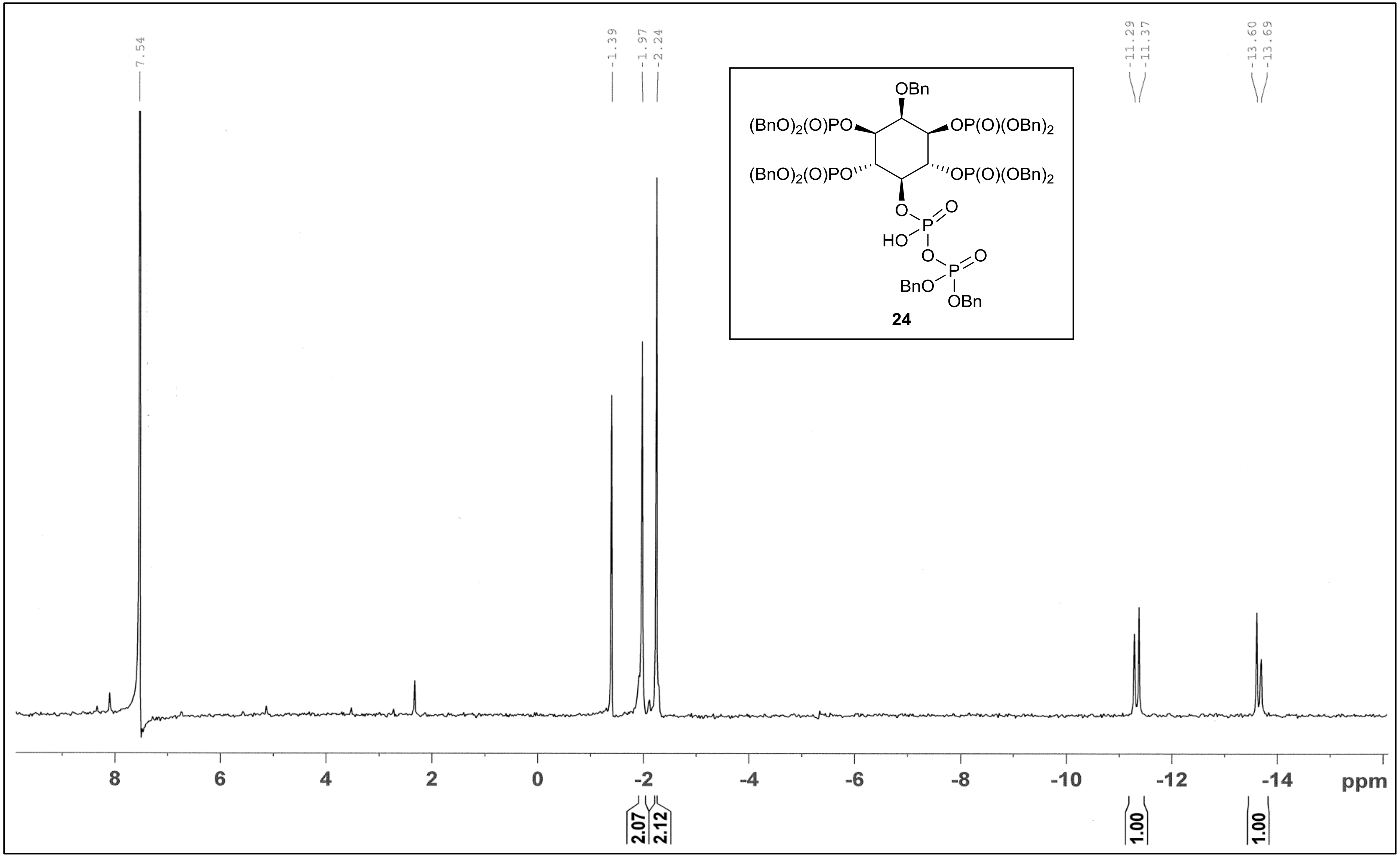

^{1}H NMR of Compound **24** (400 MHz, $CDCl_3$)

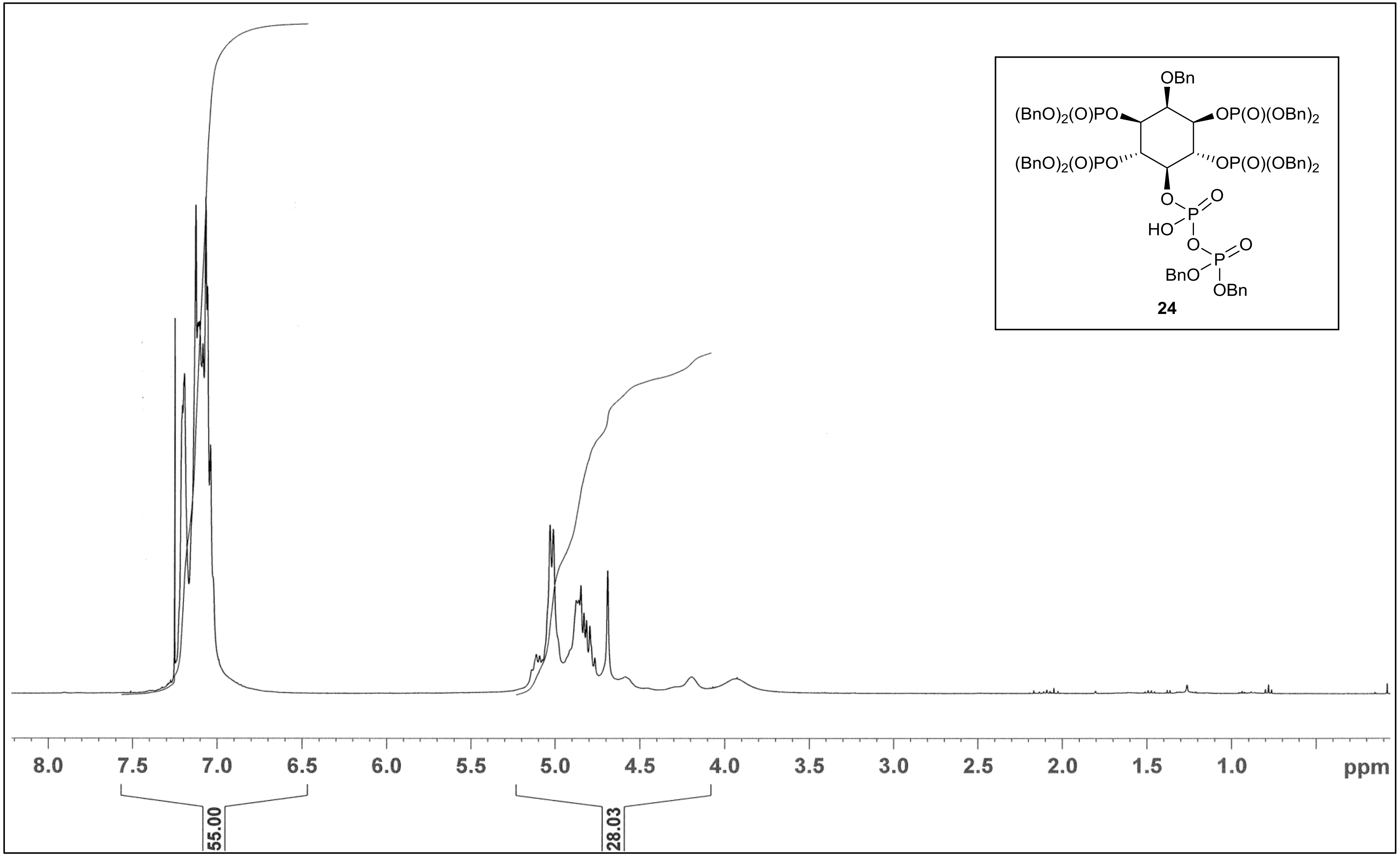

^{31}P NMR of Compound **24** (162 MHz, $CDCl_3$)

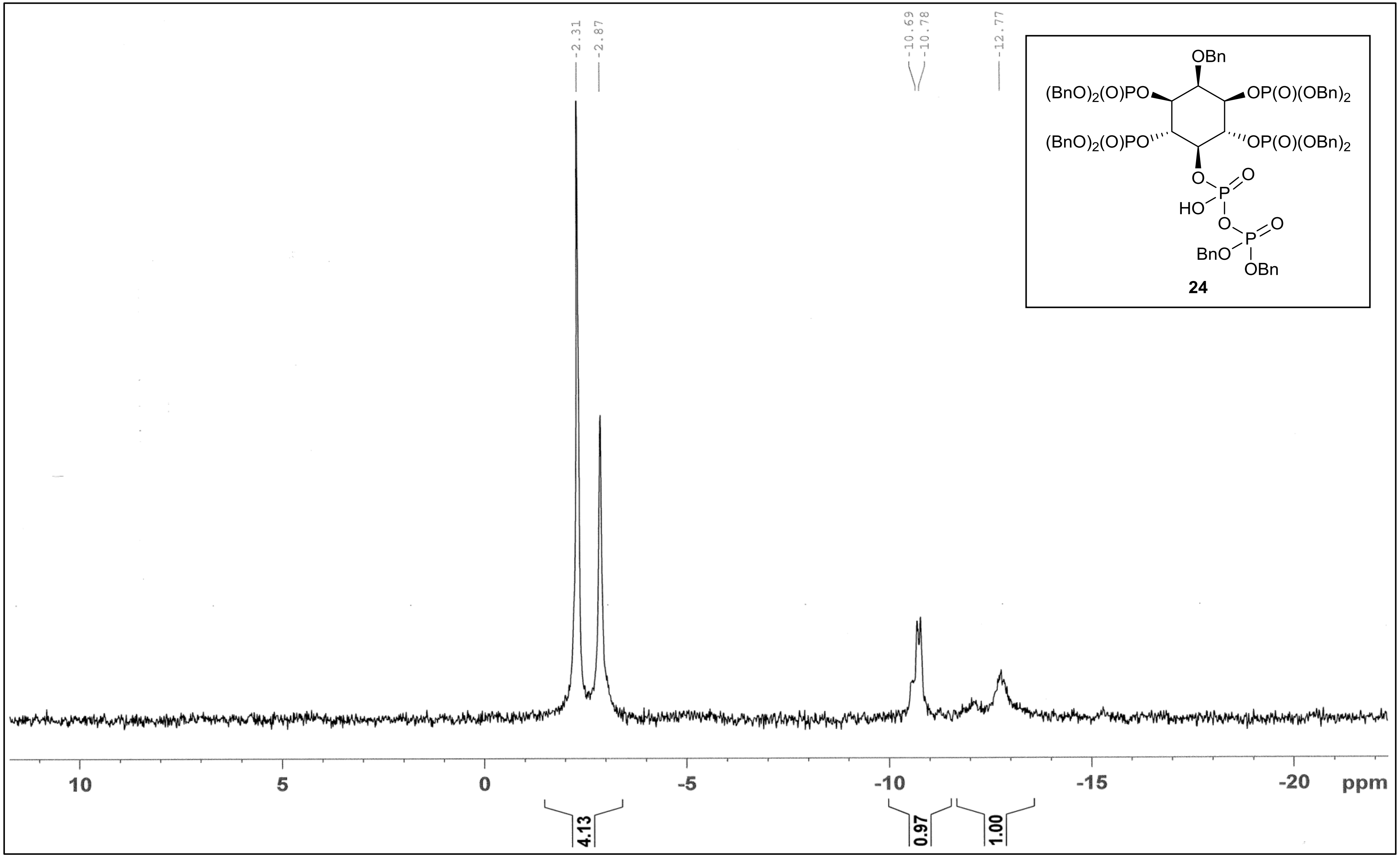

^{13}C NMR of Compound **24** (101 MHz, $CDCl_3$)

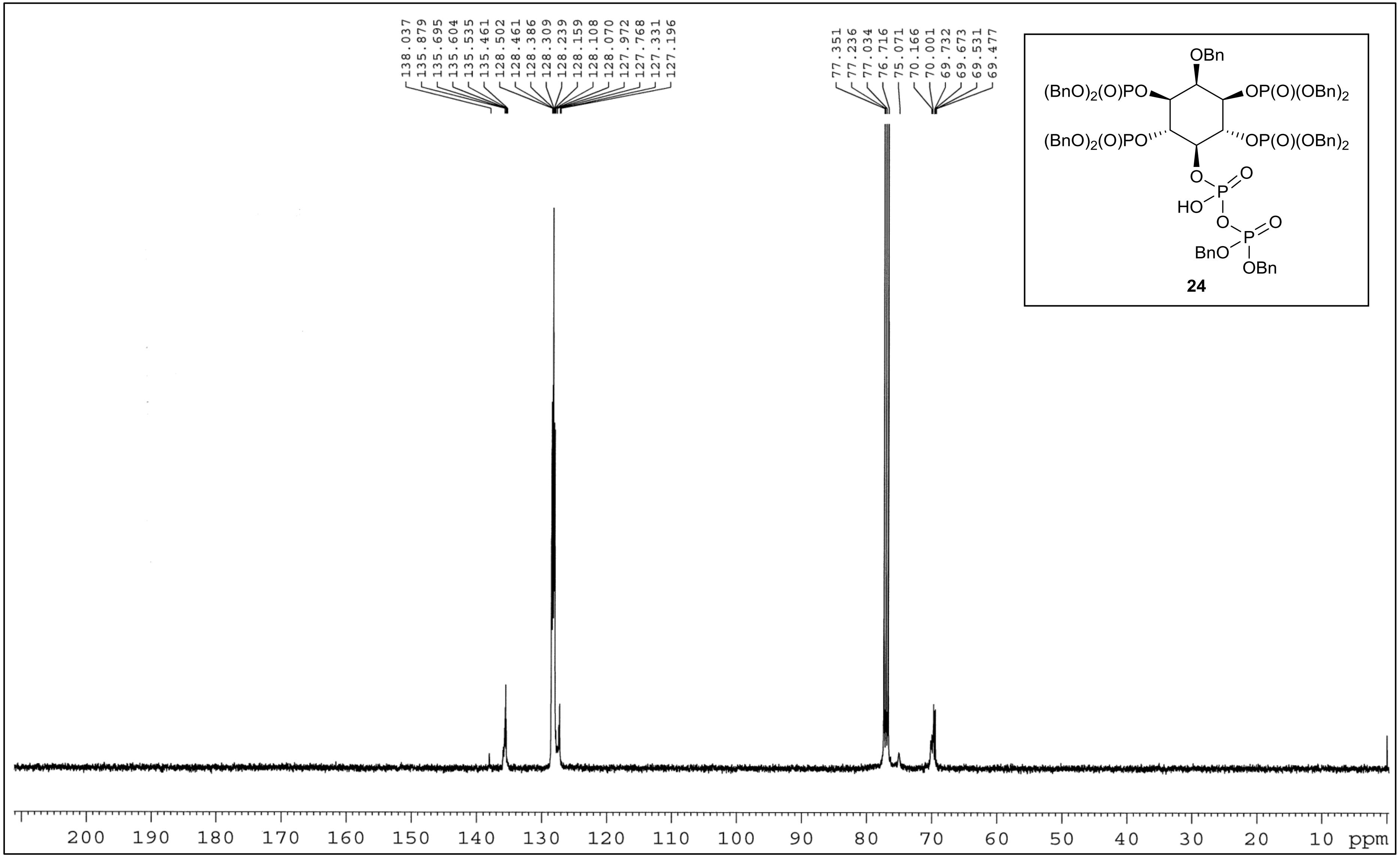

^{1}H NMR of Compound **8** (500 MHz, D_2O)

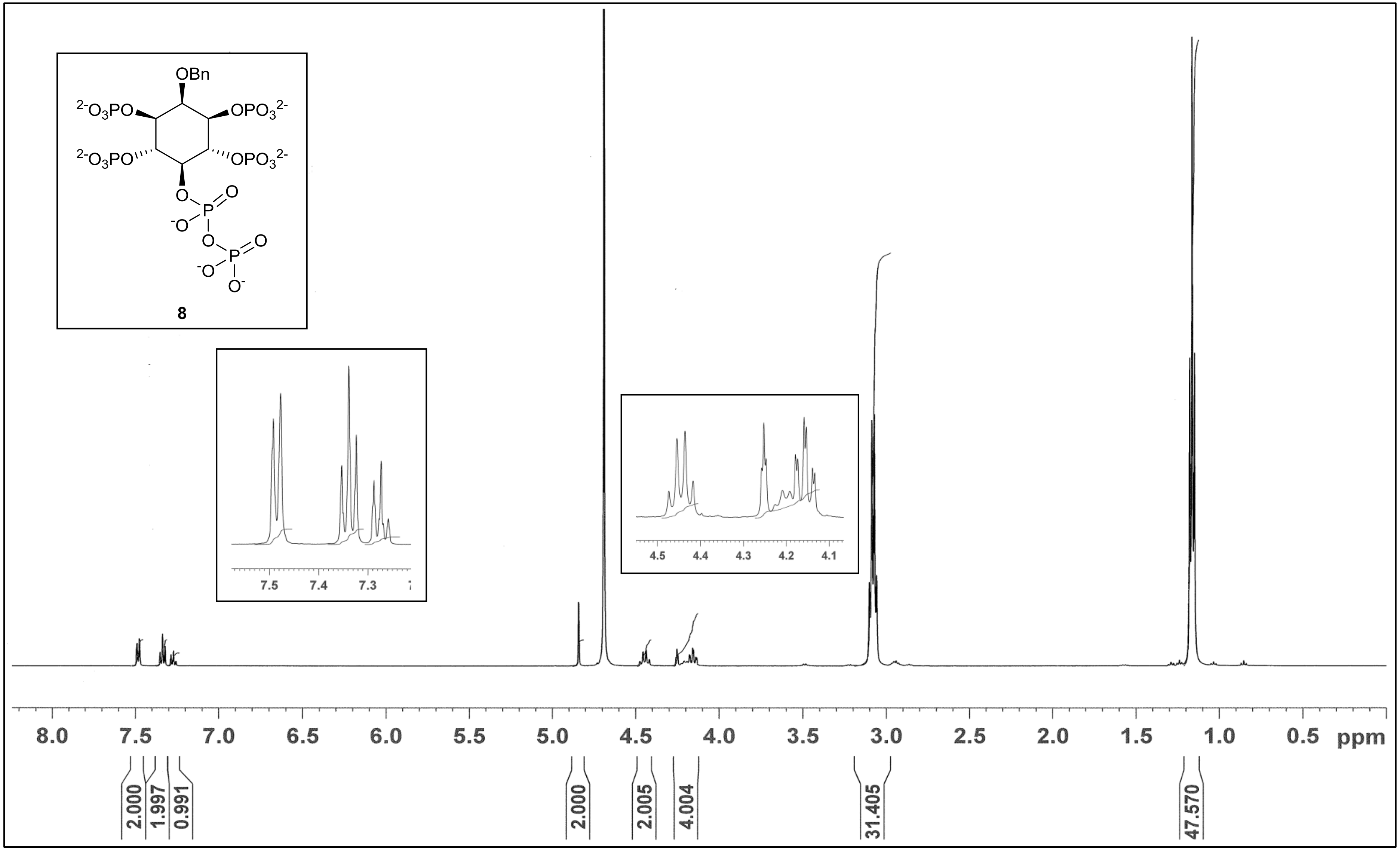

^{31}P NMR of Compound **8** (162 MHz, D_2O)

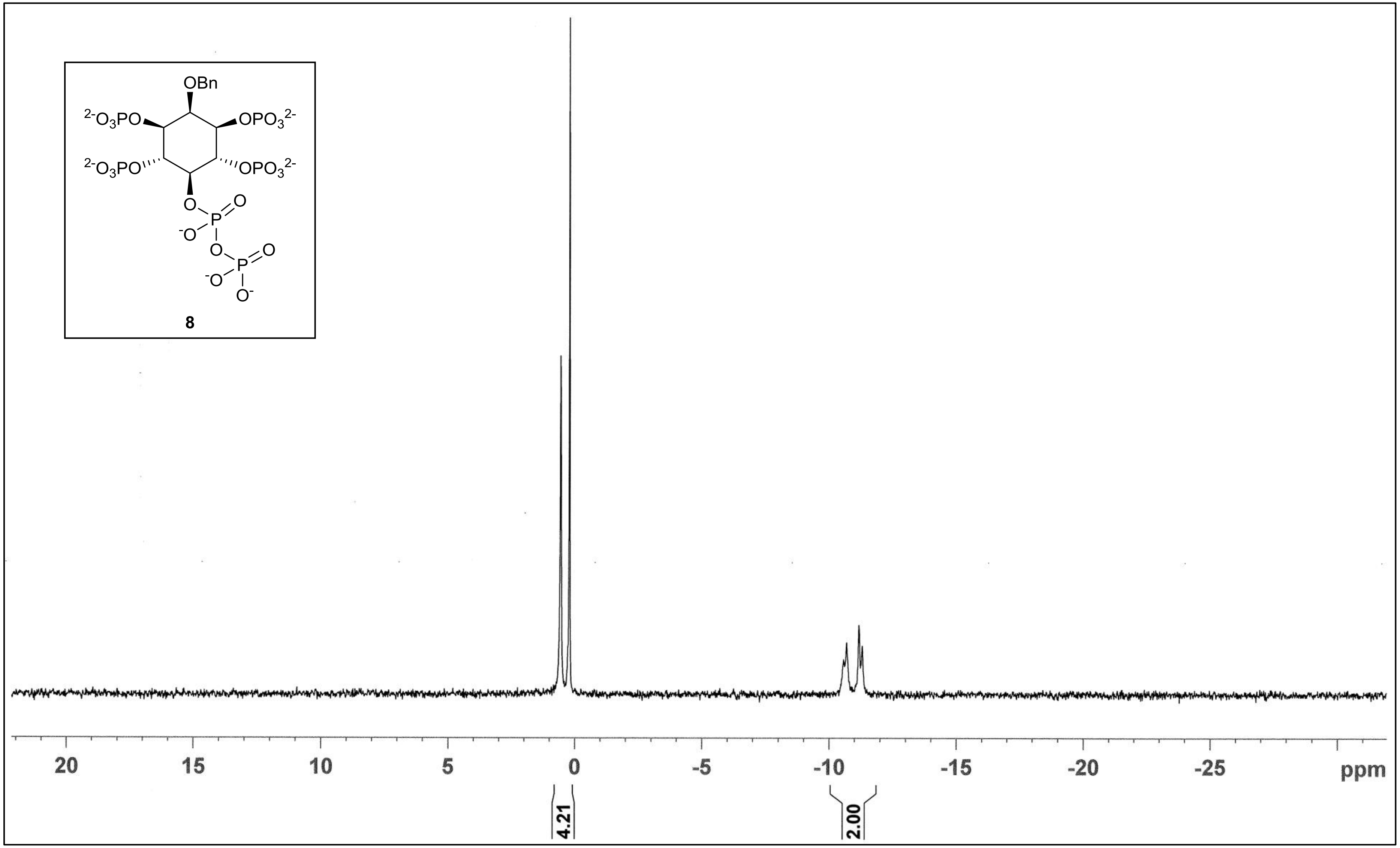

^{13}C NMR of Compound **8** (126 MHz, D_2O)

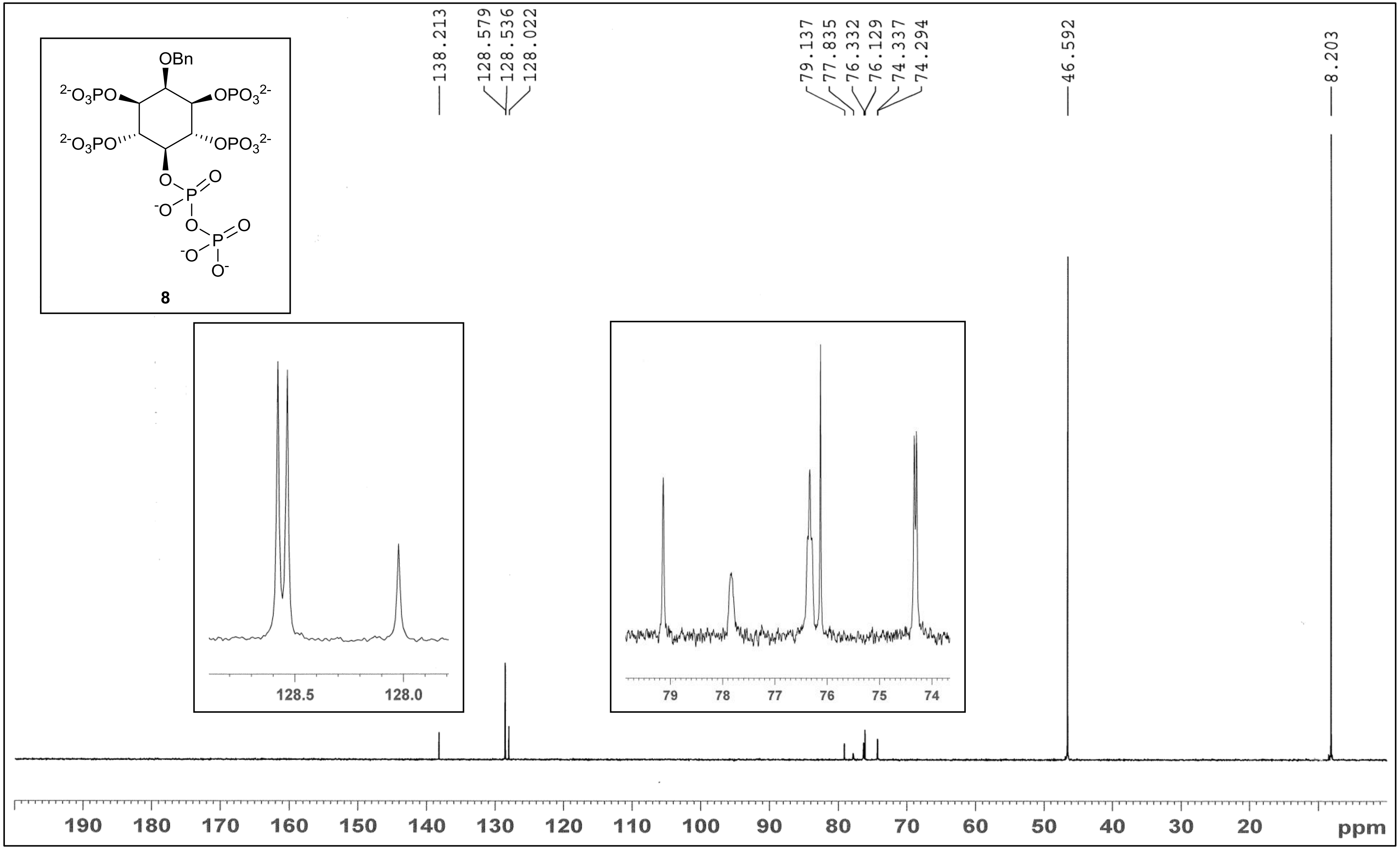

^{1}H NMR of Compound **9** (400 MHz, D_2O)

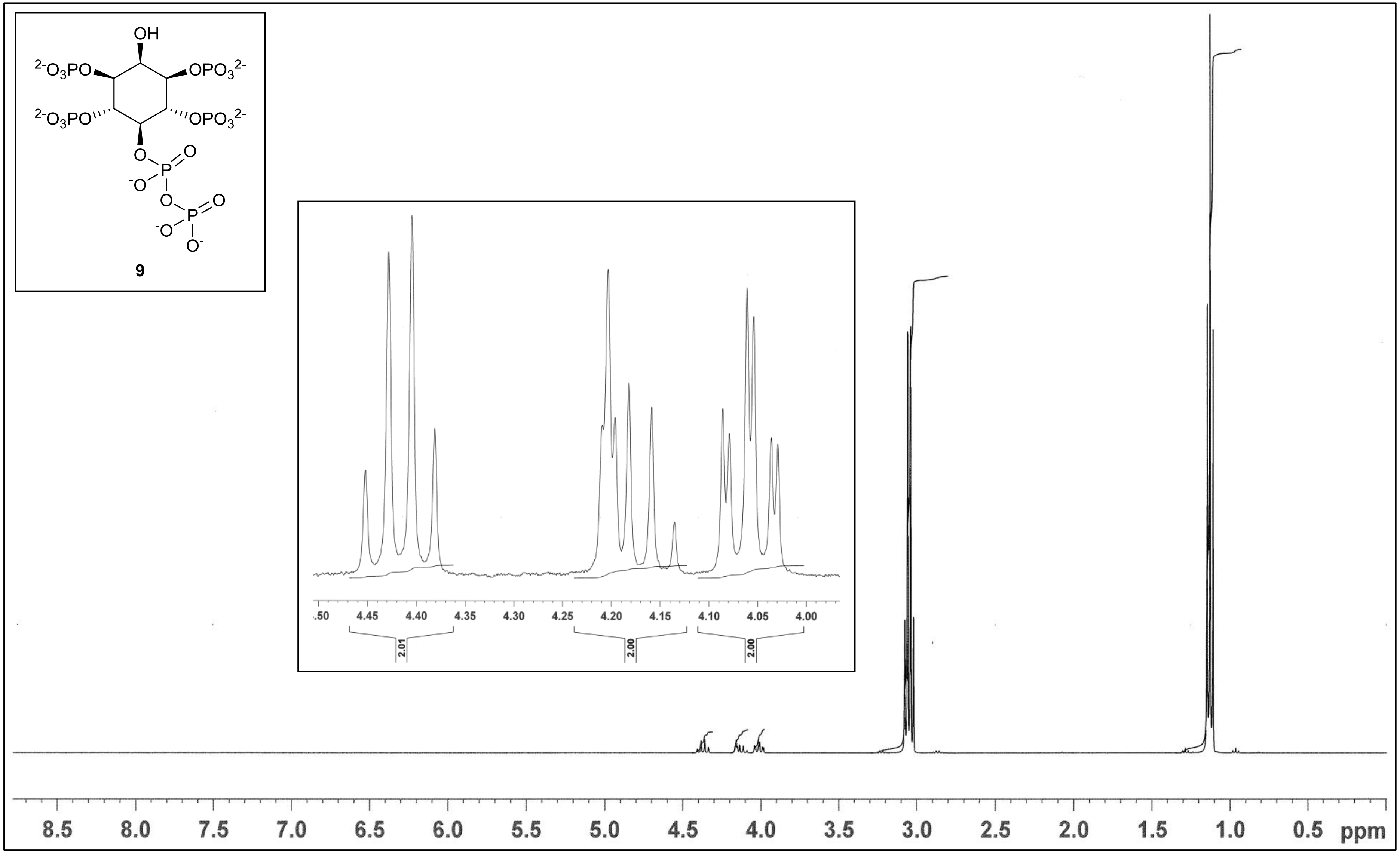

^{31}P NMR of Compound **9** (162 MHz, D_2O)

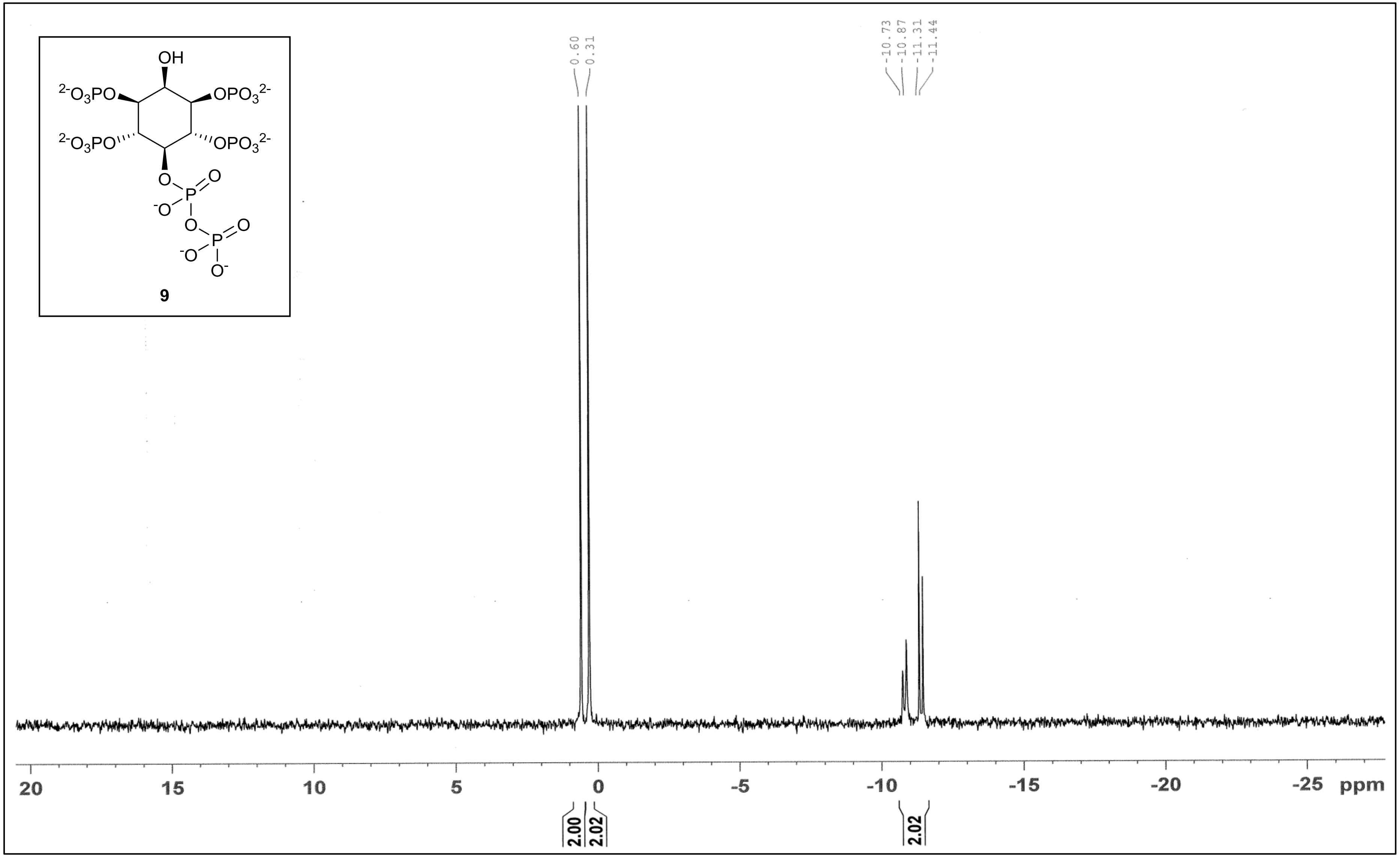

^{13}C NMR of Compound **9** (101 MHz, D_2O)

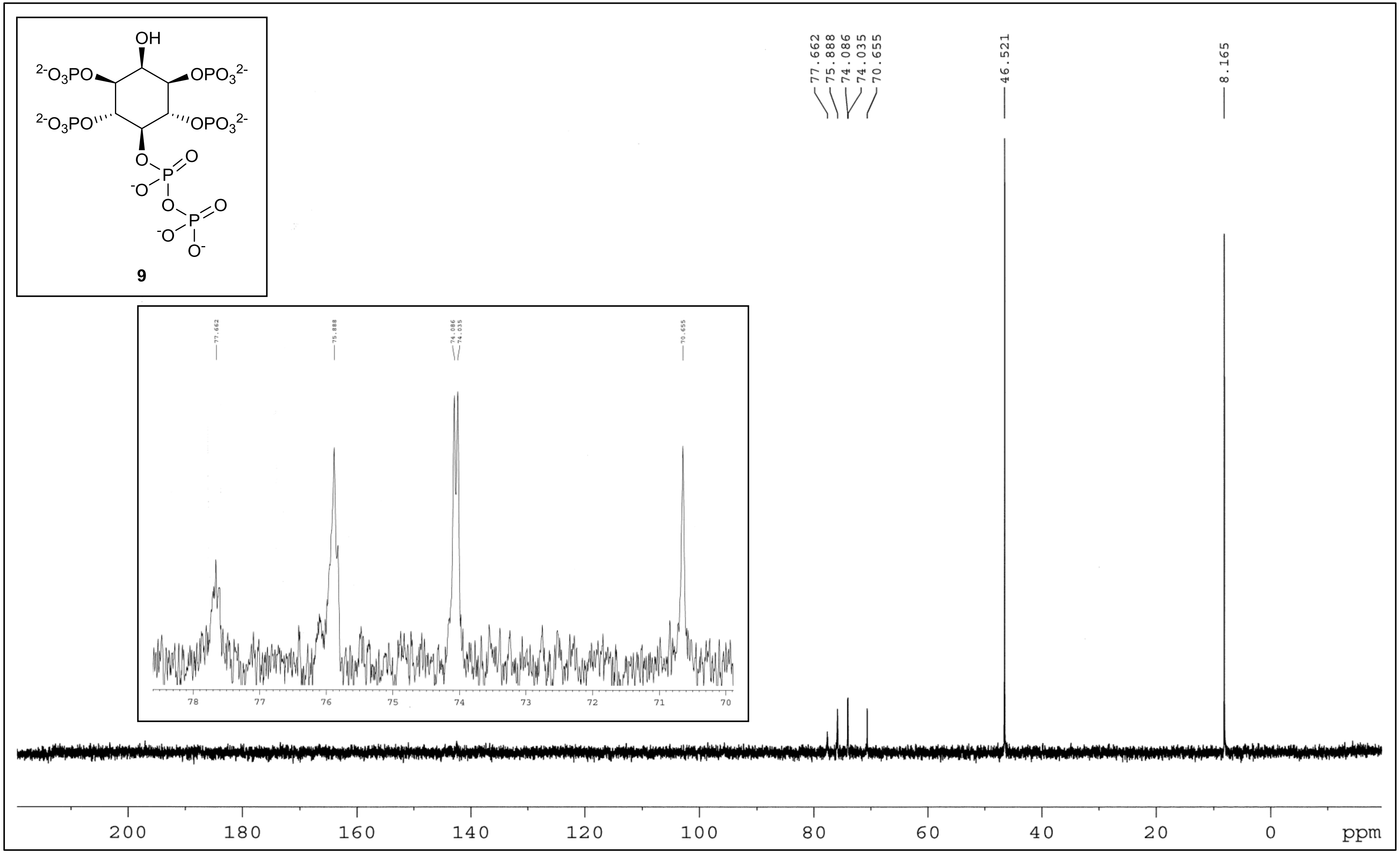

^{1}H NMR of Compound **25** (400 MHz, $CDCl_3$)

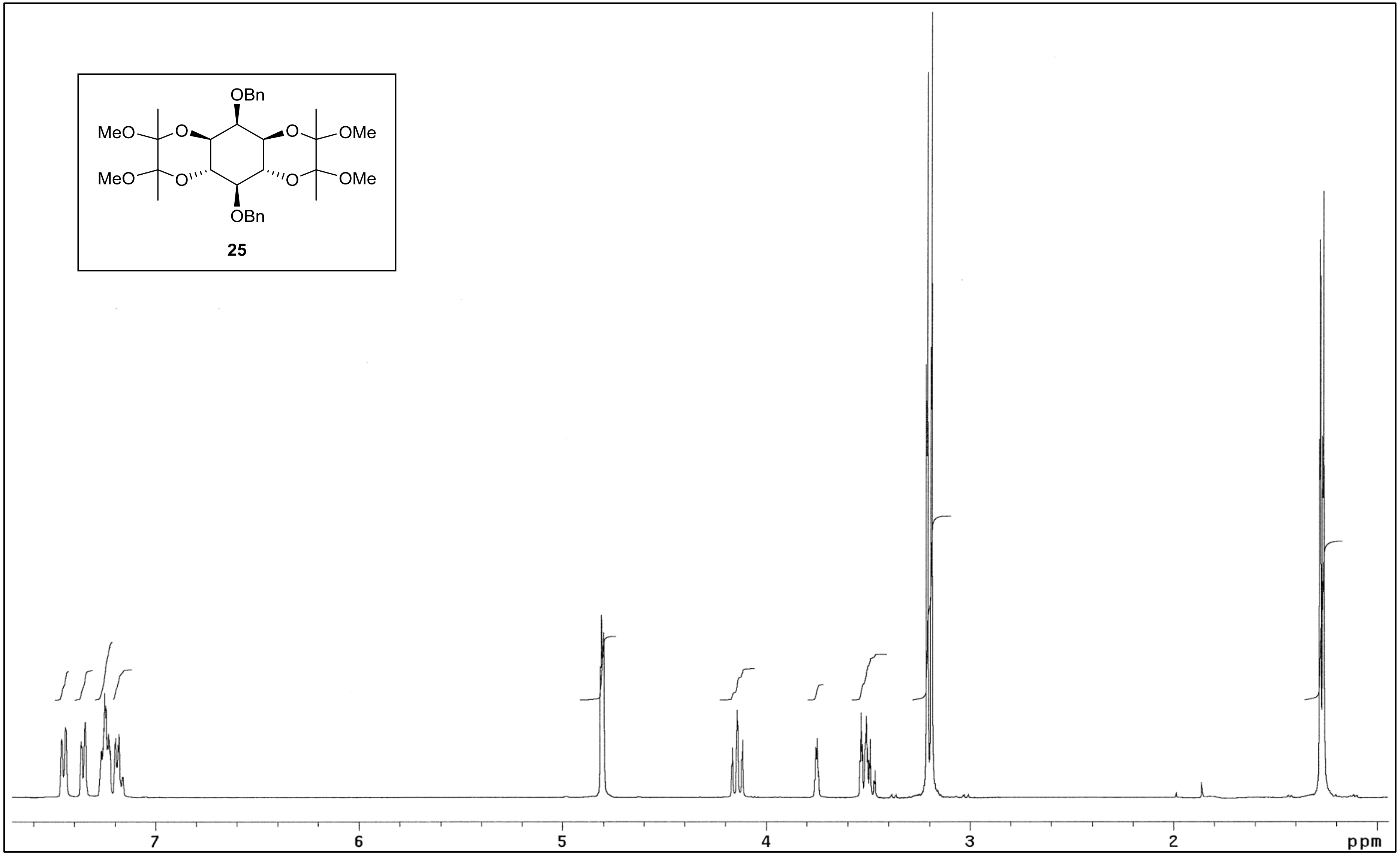

^{13}C NMR of Compound **25** (101 MHz, $CDCl_3$)

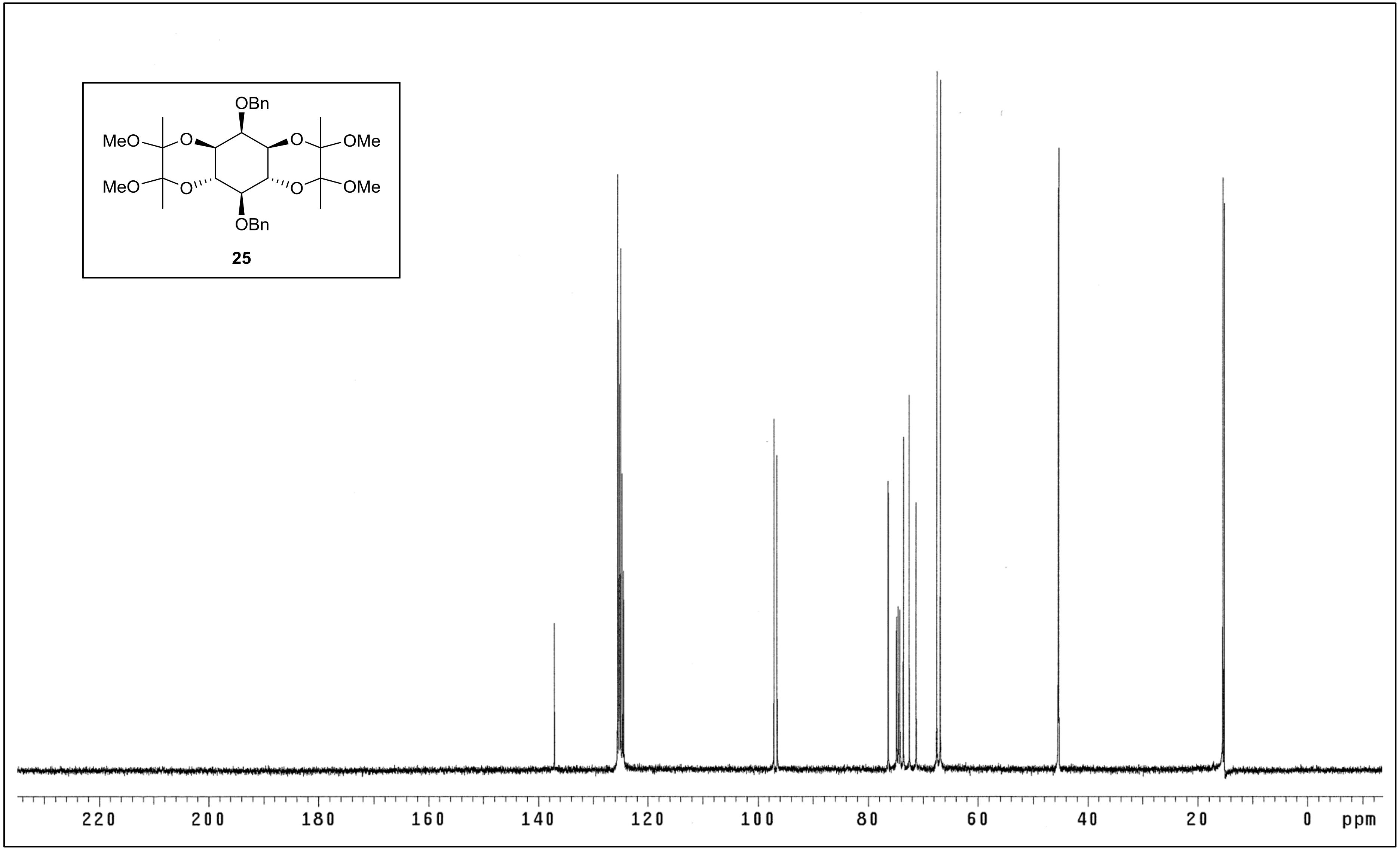

^{1}H NMR of Compound **26** (400 MHz, DMSO-d_6)

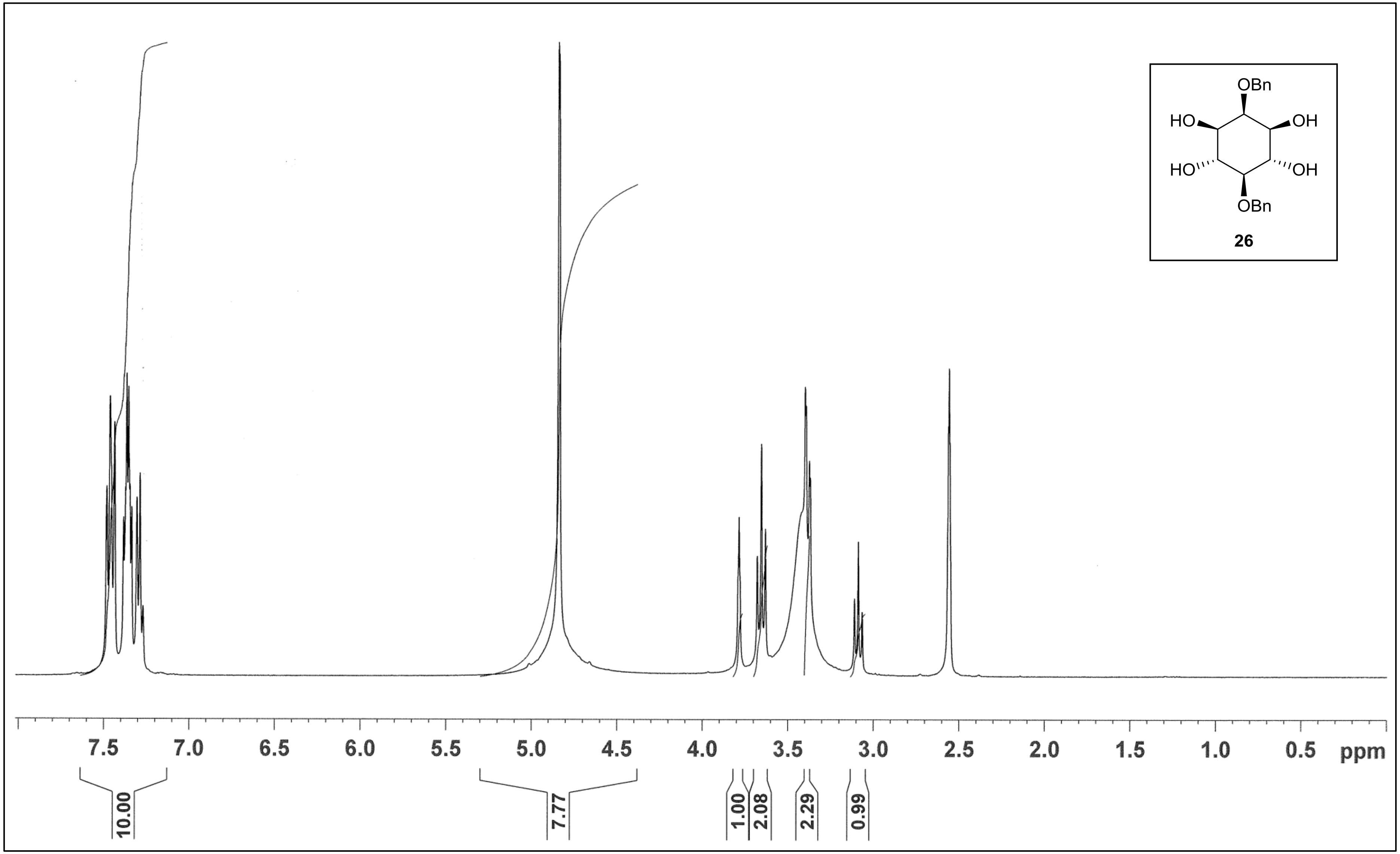

^{13}C NMR of Compound **26** (101 MHz, DMSO-d_6)

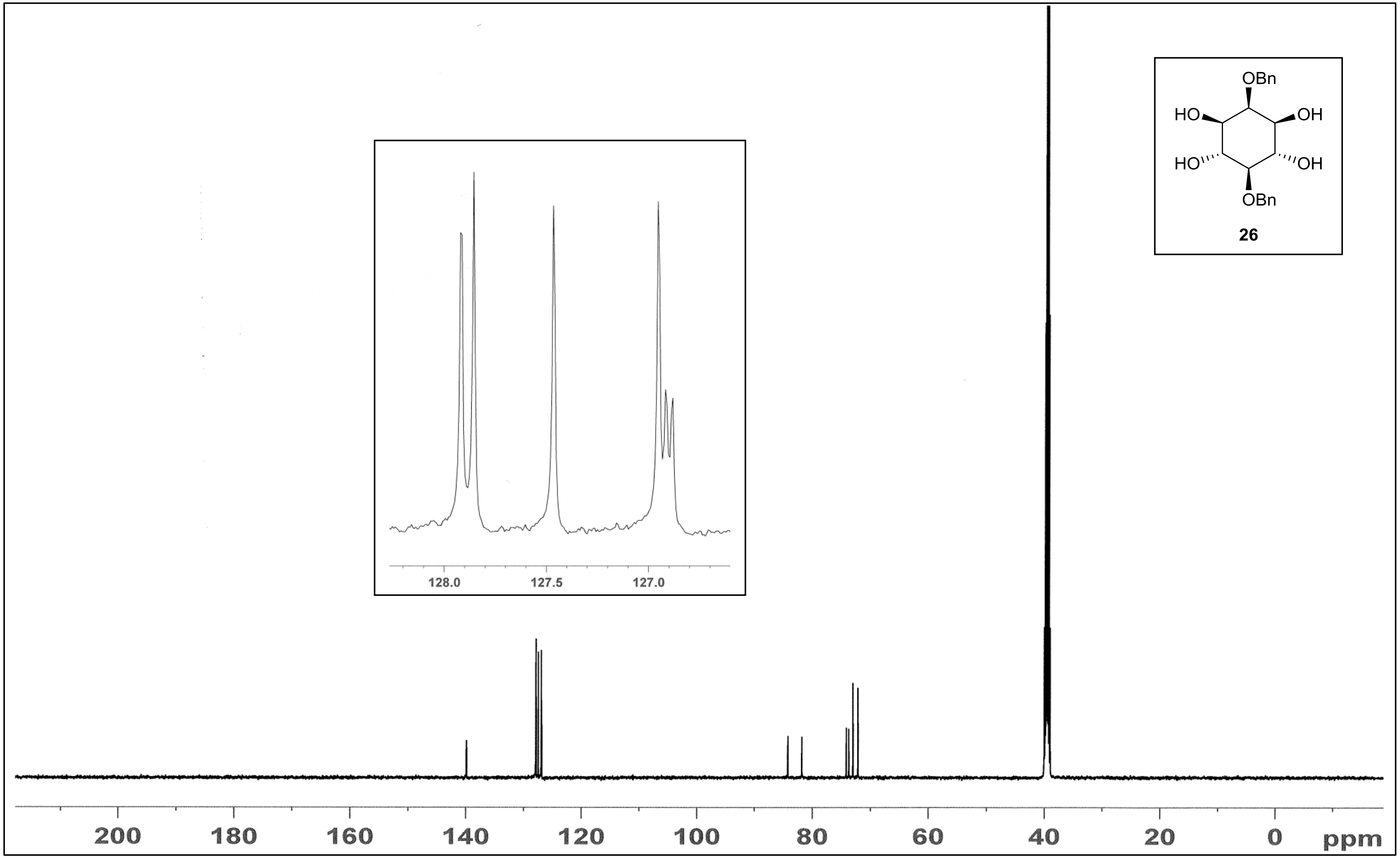

^{1}H NMR of Compound **10** (400 MHz, D_2O)

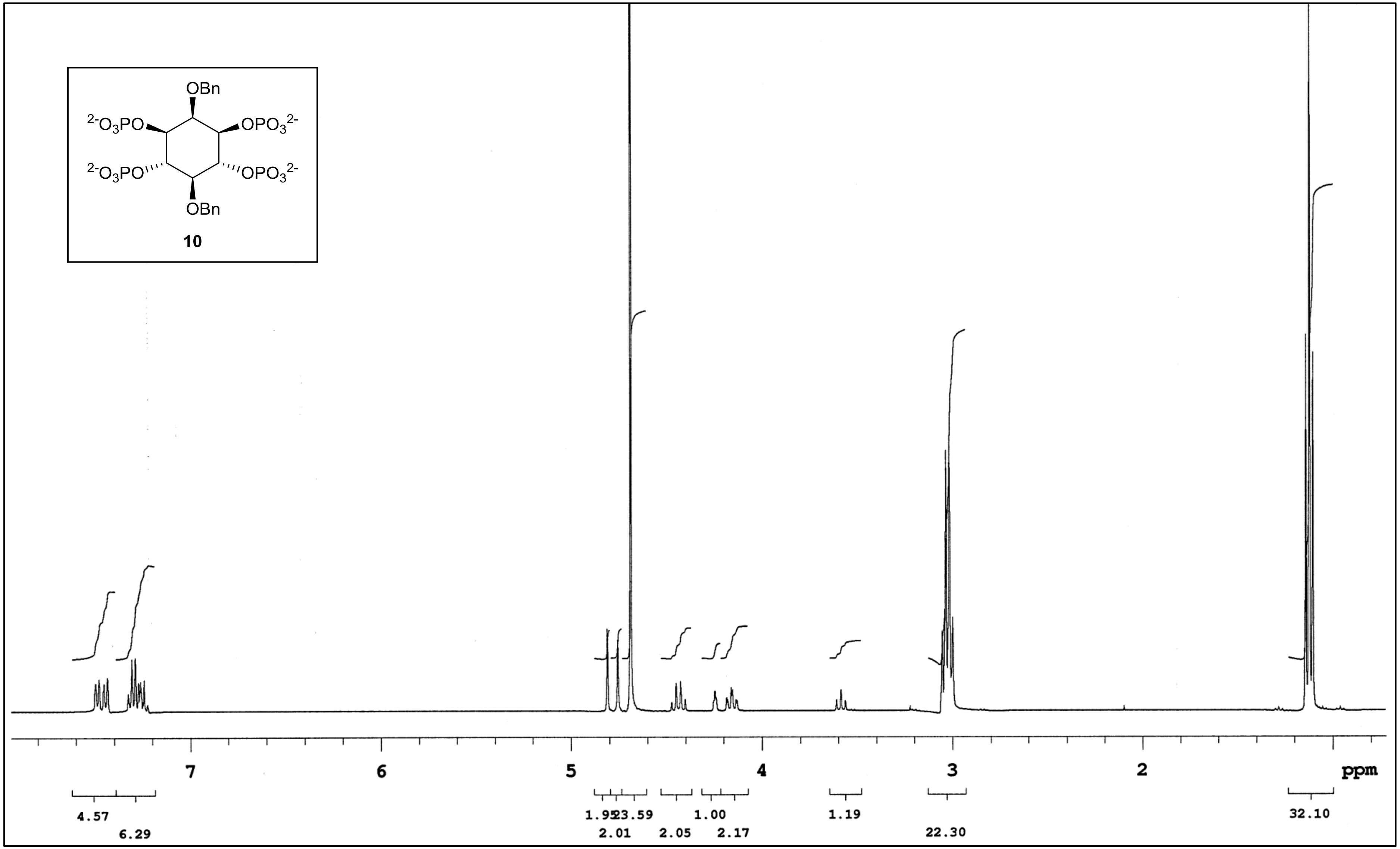

^{31}P NMR of Compound **10** (162 MHz, D_2O)

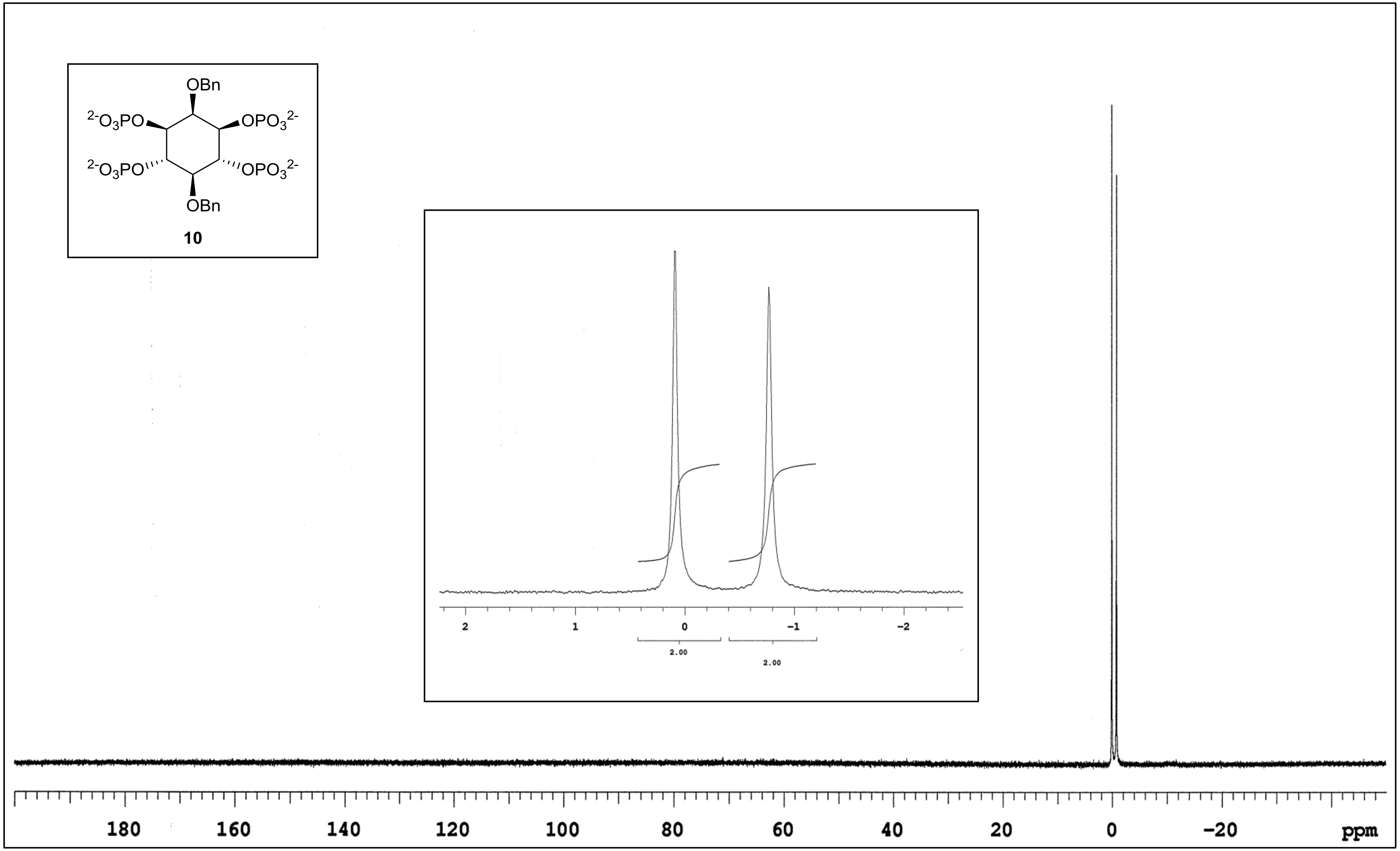

^{13}C NMR of Compound **10** (101 MHz, D_2O)

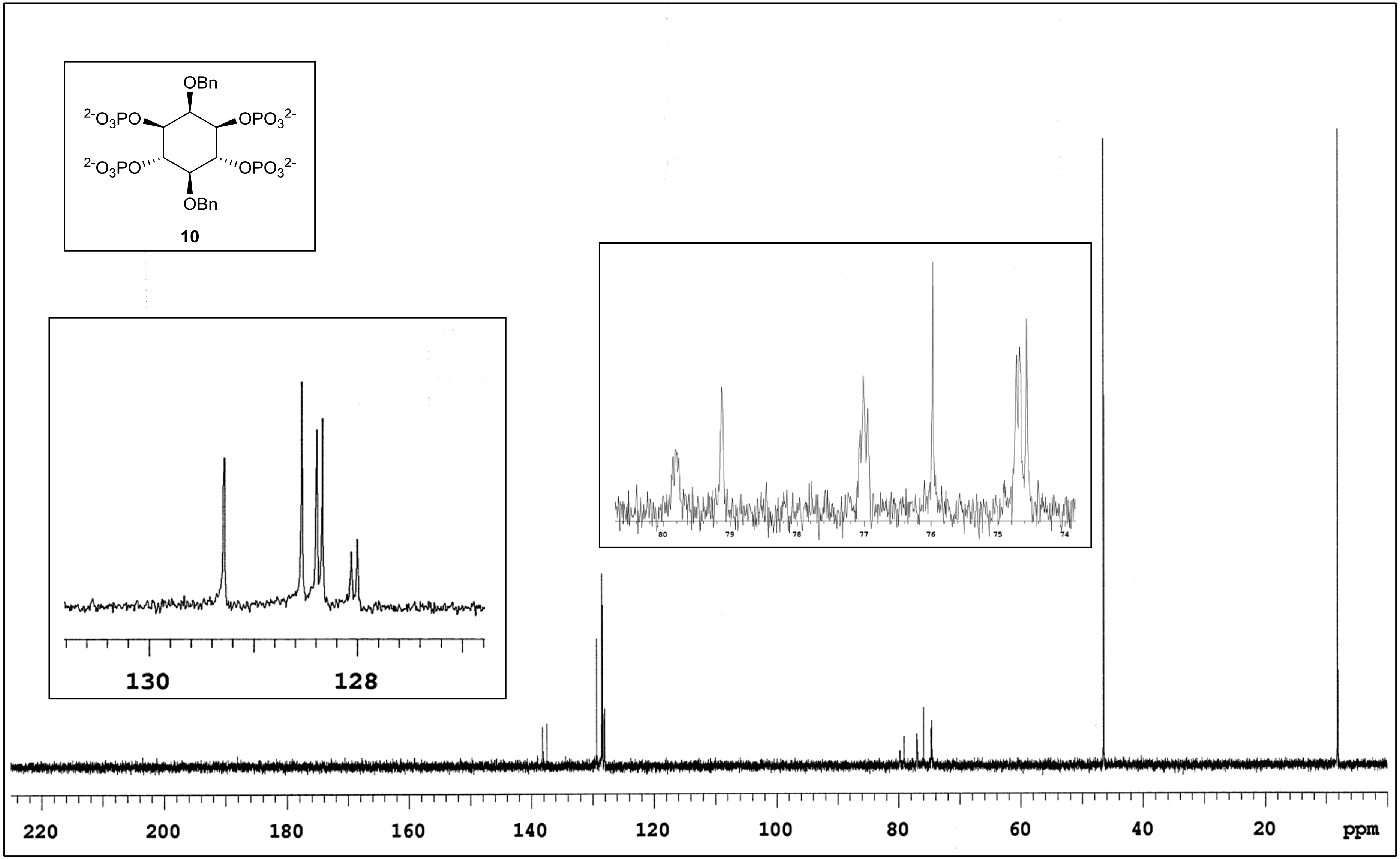

Supplement: Document S1. Supplemental Experimental Procedures, Figures S1–S3, and Table S1 [file mmc1.pdf]
